# Composite learning based finite time nonsingular terminal sliding mode path tracking control for intelligent vehicles

**DOI:** 10.1371/journal.pone.0357264

**Published:** 2026-09-01

**Authors:** Changgui Wu

**Affiliations:** The City Vocational College of Jiangsu (Nantong), Nantong, Jiangsu, P.R. China; Jiangsu University of Science and Technology, CHINA

## Abstract

This paper addresses the path tracking control problem for intelligent vehicles subject to parametric uncertainties, unmodeled dynamics, and external disturbances. A composite learning-based finite-time nonsingular terminal sliding mode control (CL-FNTSMC) strategy is proposed. Unlike conventional adaptive sliding mode controllers that rely solely on tracking errors for parameter update—and thus require the restrictive persistent excitation condition-the proposed scheme incorporates a serial–parallel estimation model to construct prediction errors, which together with tracking errors drive a composite learning law. This mechanism ensures accurate online estimation of unknown parameters under the significantly weaker interval excitation condition. A nonsingular terminal sliding surface, constructed with a continuously differentiable nonlinear function, guarantees finite time convergence while inherently avoiding singularity. Furthermore, a nonlinear disturbance observer is integrated to estimate and compensate for lumped disturbances in real time, substantially enhancing robustness. Rigorous Lyapunov-based analysis establishes the practical finite time stability of the closed loop system. Comprehensive comparative simulations under aggressive disturbances and significant parametric uncertainties demonstrate that the proposed CL-FNTSMC achieves superior tracking accuracy, faster convergence, and markedly improved disturbance rejection compared with conventional NTSMC, adaptive fast NTSMC, and PID controllers. The results confirm that the proposed framework offers an excellent balance of fast transient response, high steady-state precision, and strong robustness.

## 1. Introduction

Intelligent vehicles have emerged as a transformative technology within modern transportation systems, offering substantial potential to enhance traffic safety, operational efficiency, and environmental sustainability [1–3]. As a cornerstone enabling technology for autonomous driving, path tracking control (PTC) is instrumental in governing a vehicle’s ability to precisely adhere to a prescribed reference trajectory while maintaining dynamic stability [4]. The accuracy of PTC critically influences driving safety and ride comfort, especially in complex and rapidly changing road environments. Despite considerable research efforts, achieving high-performance path tracking for intelligent vehicles remains a persistent challenge, primarily owing to the intrinsic complexity of vehicle dynamics—which are strongly coupled, inherently nonlinear, and subject to a broad spectrum of uncertainties [5].

The challenges in PTC primarily arise from multiple sources of uncertainties, including parametric perturbations, unmodeled dynamics, and external disturbances [6–8]. These uncertainties can significantly degrade tracking accuracy and even compromise vehicle stability, necessitating the development of robust control strategies capable of handling such complexities while maintaining satisfactory transient and steady-state performance [9]. Various control methodologies have been proposed to address the path tracking problem of intelligent vehicles [10–12]. Model predictive control (MPC) has gained substantial popularity due to its ability to handle constraints and predict future system behavior [13]. In [14], Sun et al. tackle the issue of insufficient adaptability of conventional MPC to varying driving scenarios in autonomous vehicle path tracking. In [15], Nascimento et al. address the challenges of MPC design for nonholonomic mobile robot trajectory tracking by providing a comprehensive survey that covers modeling, constraint handling, stability analysis, and computational considerations. However, MPC typically relies on accurate system models and may suffer from substantial computational burden, limiting its applicability in real-time implementations [16]. Linear quadratic regulator (LQR) and PID based methods are simple [17–19]. In [20], Sun et al. address the challenge of coordinating lateral and longitudinal control for intelligent vehicles in the presence of varying road curvatures and future path changes by proposing a LQR based control method that incorporates preview information to optimize tracking performance. Jiang and Palaoag address the trajectory tracking problem for autonomous systems by proposing a hybrid algorithm that integrates MPC with LQR, leveraging MPC’s constraint handling capability and LQR’s optimality for enhanced tracking performance [21]. However, they often lack robustness to significant uncertainties and disturbances.

Recent years have witnessed a surge of interest in sliding mode control (SMC) for vehicle path tracking, as evidenced by several comprehensive survey papers [22,23]. These reviews systematically categorize various SMC methodologies—including terminal sliding mode, adaptive sliding mode, and disturbance-observer-based sliding mode—and identify their respective merits and limitations when applied to ground vehicles. Notably, [22] highlights that while SMC offers inherent robustness against matched uncertainties, its application to vehicle path tracking is often hindered by chattering, singularity, and the stringent persistent excitation (PE) condition required for adaptive parameter convergence. These challenges motivate the development of integrated solutions that combine nonlinear control techniques with advanced learning mechanisms. SMC has emerged as a powerful robust control technique for nonlinear systems with uncertainties, owing to its insensitivity to matched disturbances and its capability to enforce desired system behavior through discontinuous control actions [24–26]. In [27], Ansari et al. address the load frequency control problem in power systems subject to parameter uncertainties and external disturbances by proposing a robust backstepping SMC that integrates the systematic design of backstepping with the robustness of sliding mode control to ensure frequency stability. In [5], Zhang et al. address the trajectory tracking control problem of permanent magnet synchronous motors subject to parameter uncertainties, load disturbances, and sliding mode chattering by proposing a non-singular fast terminal SMC scheme with disturbance compensation. In [28], Zhang et al. tackle the challenges of finite-time convergence and disturbance sensitivity in robotic manipulator control. They propose a fixed-time SMC integrated with a disturbance observer, which guarantees global fast tracking convergence with a convergent time upper bound independent of initial states, while effectively estimating and compensating for lumped disturbances. In [29], Tan et al. address the spacecraft reorientation control problem under multiple attitude constraints and actuator resource limitations by proposing an event-triggered sliding mode control scheme, which achieves robust attitude tracking while significantly reducing actuator updates and ensuring the avoidance of Zeno behavior. More recently, several studies have specifically explored SMC for autonomous vehicle applications. In [30], Wang et al. proposes an adaptive sliding mode approach for vehicle lateral control under uncertain tire-road friction conditions. In [31], Li et al. develops a finite-time terminal sliding mode controller for path tracking of autonomous vehicles, demonstrating improved transient performance. However, these methods either require stringent PE conditions for parameter convergence or lack a systematic mechanism for disturbance estimation and compensation, leaving room for further improvement.

Conventional adaptive controllers rely on the persistent excitation (PE) condition for parameter convergence, which is seldom satisfied in real driving scenarios. Consequently, tracking-error-driven controllers such as AFNTSMC fail to ensure parameter convergence [32], degrading model fidelity and tracking accuracy. The composite learning mechanism overcomes this by achieving reliable estimation under interval excitation—a weaker and more practical condition than PE. Motivated by the aforementioned discussions, this paper proposes a composite learning-based finite-time nonsingular terminal sliding mode control (CL-FNTSMC) strategy for intelligent vehicle path tracking. The main contributions are threefold:

1. A unified path tracking error model is established that explicitly accounts for lumped uncertainties encompassing parametric variations, unmodeled dynamics, and external disturbances. A nonlinear disturbance observer is incorporated to provide real-time estimation and feedforward compensation, effectively reducing the conservativeness of robust control design.
2. A composite learning law is developed that simultaneously exploits tracking errors and prediction errors from a serial-parallel estimation model, enabling accurate online parameter estimation without the restrictive PE condition. Theoretical analysis establishes parameter convergence under interval excitation, which is strictly weaker than PE and practically more relevant.
3. Non-singular terminal sliding surface is designed to have a nonlinear function of continuously differentiable, which ensures finite time convergence while inherently avoiding singularity. The proposed framework cooperatively integrates compound learning, finite time sliding mode and interference observation, and is supported by strict Lyapunov-based stability analysis, which ensures that tracking error, parameter estimation and interference estimation converge simultaneously. A large number of comparative simulations in different scenarios confirm that the proposed method is obviously superior to PID, traditional NTSMC and the most advanced AFNTSMC.

The remainder of this paper is organized as follows. Section 2 presents the problem formulation and the vehicle path tracking model. Section 3 details the design of the proposed CL-FNTSMC strategy, including the composite learning laws, the nonsingular terminal sliding surface, the disturbance observer, and the stability analysis. Section 4 presents simulation results and comparative studies. Finally, Section 5 concludes the paper with discussions and future research directions.

## 2. Problem description and vehicle modeling

This section establishes the mathematical foundation for path tracking control of intelligent vehicles. Both kinematic and dynamic characteristics are incorporated to capture the essential lateral behavior. The resulting model is cast into a compact state-space representation that explicitly accounts for parametric uncertainties, unmodeled dynamics, and external disturbances, thereby facilitating the subsequent control design.

### 2.1. Vehicle kinematic model

Consider an intelligent vehicle moving in the horizontal plane. The vehicle pose is described by the global position coordinates (*X*,*Y*) and the yaw angle ψ, measured with respect to a fixed inertial frame {ℐ}. The generalized coordinate vector is defined as $\eta =[X,Y,\psi {]}^{T}\in {\mathbb{R}}^{3}$. Under the nonholonomic constraint of no lateral slip at the rear axle, the kinematic equations follow the standard bicycle model:


$$\begin{array}{c} \dot{X}=v\cos\psi , \end{array}$$ (1a)

$$\begin{array}{c} \dot{Y}=v\sin\psi , \end{array}$$ (1b)

$$\begin{array}{c} \dot{\psi }=\omega , \end{array}$$ (1c)

where *v* and ω denote the longitudinal velocity at the vehicle center of gravity (CG) and the yaw rate, respectively. At the kinematic level, both *v* and ω serve as control inputs.

Let the desired reference path 𝒫 be parameterized by arc length *s*, with the desired pose denoted as $\eta_{d}(s)=[X_{d}(s),Y_{d}(s),\psi_{d}(s){]}^{T}$. The path curvature κ(s) relates to the desired yaw rate via $\omega_{d}={\dot{\psi }}_{d}=\kappa (s)\dot{s}$. Assuming the path is tracked at the desired longitudinal speed $v_{d}$, we have $\dot{s}=v_{d}$.

To facilitate error-based control design, the tracking errors are defined in the vehicle body-fixed frame. The rotation matrix from the inertial frame to the body frame is given by


$$\begin{array}{c} 𝐑(\psi )=[\begin{array}{ccc} \cos\psi & \sin\psi & 0\\ -\sin\psi & \cos\psi & 0\\ 0 & 0 & 1 \end{array}]. \end{array}$$ (2)

The error vector in the body frame is then defined as


$$\begin{array}{c} {}[\begin{array}{c} e_{x}\\ e_{y}\\ e_{\psi } \end{array}]=𝐑(\psi )[\begin{array}{c} X-X_{d}\\ Y-Y_{d}\\ \psi -\psi_{d} \end{array}], \end{array}$$ (3)

where $e_{x}$, $e_{y}$, and $e_{\psi }$ denote the longitudinal error, lateral (cross-track) error, and yaw angle error, respectively. Differentiating (3) with respect to time and substituting (1) yields the kinematic error dynamics:


$$\begin{array}{c} {\dot{e}}_{x}=v\cos e_{\psi }-v_{d}+\omega e_{y}, \end{array}$$ (4a)

$$\begin{array}{c} {\dot{e}}_{y}=v\sin e_{\psi }-\omega e_{x}, \end{array}$$ (4b)

$$\begin{array}{c} {\dot{e}}_{\psi }=\omega -\omega_{d}. \end{array}$$ (4c)

In typical driving scenarios, the longitudinal error $e_{x}$ is regulated by throttle/brake control, whereas lateral control is predominantly achieved through steering. In this study, we assume that a lower-level longitudinal controller maintains a constant forward speed, i.e., $v=v_{d}$, and we focus exclusively on the lateral path tracking problem. Under this assumption, and invoking the small-angle approximation $\sin e_{\psi }\approx e_{\psi }$ and $\cos e_{\psi }\approx 1$, which is valid for normal driving conditions—the lateral error dynamics simplify to


$$\begin{array}{c} {\dot{e}}_{y}=ve_{\psi },\;{\dot{e}}_{\psi }=\omega -\omega_{d}. \end{array}$$ (5)

It is important to note, however, that the kinematic model neglects inertial effects and tire–road interaction forces, which become significant at higher speeds. Consequently, a dynamic model is required for robust controller design in practical driving scenarios.

**Remark 1:** The kinematic model (5) provides a geometric description of the vehicle–path relationship under the assumptions of small slip angles and constant longitudinal speed. While it serves as a useful baseline for understanding the fundamental tracking geometry, it is inadequate for high-speed maneuvers where lateral acceleration and yaw dynamics dominate. The subsequent subsection addresses these limitations by introducing a dynamic vehicle model.

### 2.2. Vehicle dynamic model

To incorporate the essential inertial and tire-force characteristics, we adopt the widely used single-track (bicycle) dynamic model. The vehicle is assumed to have front-wheel steering only, and the state variables are defined as the longitudinal velocity $v_{x}$, lateral velocity $v_{y}$, and yaw rate ω in the body frame. The equations of motion are:


$$\begin{array}{c} {\dot{v}}_{x}=\frac{1}{m}\left(F_{xf}\cos\delta_{f}-F_{yf}\sin\delta_{f}+F_{xr}\right)+v_{y}\omega , \end{array}$$ (6a)

$$\begin{array}{c} {\dot{v}}_{y}=\frac{1}{m}\left(F_{xf}\sin\delta_{f}+F_{yf}\cos\delta_{f}+F_{yr}\right)-v_{x}\omega , \end{array}$$ (6b)

$$\begin{array}{c} \dot{\omega }=\frac{1}{I_{z}}\left(l_{f}F_{yf}\cos\delta_{f}-l_{r}F_{yr}+l_{f}F_{xf}\sin\delta_{f}\right), \end{array}$$ (6c)

where *m* is the vehicle mass, $I_{z}$ is the yaw moment of inertia, and $l_{f}$ and $l_{r}$ are the distances from the centre of gravity (CG) to the front and rear axles, respectively; $\delta_{f}$ is the front steering angle, which serves as the control input; $F_{xf}$ and $F_{xr}$ are the longitudinal tire forces at the front and rear; and $F_{yf}$ and $F_{yr}$ are the lateral tire forces.

For moderate lateral accelerations, the lateral tire forces can be approximated by a linear relationship with the corresponding slip angles:


$$\begin{array}{c} F_{yf}=C_{f}\alpha_{f},\;F_{yr}=C_{r}\alpha_{r}, \end{array}$$ (7)

where $C_{f}$ and $C_{r}$ are the cornering stiffnesses (positive constants), and $\alpha_{f}$ and $\alpha_{r}$ are the front and rear slip angles, respectively, given by:


$$\begin{array}{c} \alpha_{f}=\delta_{f}-\frac{v_{y}+l_{f}\omega }{v_{x}},\;\alpha_{r}=\frac{l_{r}\omega -v_{y}}{v_{x}}. \end{array}$$ (8)

To facilitate control design, we assume that the longitudinal speed is maintained at a nearly constant value $v_{x}\approx v$ by a separate controller. Under this assumption, the longitudinal dynamics can be neglected. Furthermore, we ignore the longitudinal tire forces ($F_{xf}=F_{xr}=0$), as the steering control primarily affects the lateral motion. Substituting the linear tire force expressions into Eq. (6) and linearising about a straight-line motion with small steering angles, we obtain the following lateral-yaw dynamics:


$$\begin{array}{c} {\dot{v}}_{y}=-\frac{C_{f}+C_{r}}{mv}v_{y}+\left(\frac{C_{f}+C_{r}}{m}-v\right)\omega +\frac{C_{f}}{m}\delta_{f}, \end{array}$$ (9a)

$$\begin{array}{c} \dot{\omega }=-\frac{l_{f}C_{f}-l_{r}C_{r}}{I_{z}v}v_{y}-\frac{l_{f}^{2}C_{f}+l_{r}^{2}C_{r}}{I_{z}v}\omega +\frac{l_{f}C_{f}}{I_{z}}\delta_{f}. \end{array}$$ (9b)

In the above derivation, we have used the small-angle approximations $\cos\delta_{f}\approx 1$ and $\sin\delta_{f}\approx \delta_{f}$, together with the slip-angle expressions $\alpha_{f}\approx \delta_{f}-(v_{y}+l_{f}\omega )/v$ and $\alpha_{r}\approx (l_{r}\omega -v_{y})/v$. The term *v* appearing in the coefficient of ω in Eq. (9a) arises from the centripetal acceleration term vω after linearisation.

**Remark 2:** The linear tire model is valid only for small slip angles. Under higher lateral accelerations, nonlinear tire characteristics become significant and should be considered. Nevertheless, for the purpose of control design, the linear model provides a reasonable approximation. The unmodeled nonlinearities and parameter variations are treated as lumped disturbances, which will be compensated by the proposed adaptive neural network controller in the following section.

### 2.3. Path tracking error dynamics

To facilitate the controller design for path tracking, we express the vehicle dynamics in terms of the path tracking errors defined in Eq. (3). Under the assumptions of small yaw error $e_{\psi }$ and constant longitudinal speed *v*, the lateral error $e_{y}$ and yaw error $e_{\psi }$ are related to the vehicle states $v_{y}$ and ω by the following kinematic relations:


$$\begin{array}{c} {\dot{e}}_{y}=ve_{\psi }+v_{y},\;{\dot{e}}_{\psi }=\omega -\omega_{d}, \end{array}$$ (10)

where $\omega_{d}$ denotes the desired yaw rate corresponding to the reference path curvature.

Taking the time derivatives of the above equations and substituting the dynamic equations from Eq. (9), we obtain the second-order error dynamics:


$$\begin{array}{c} {\ddot{e}}_{y}=-\frac{C_{f}+C_{r}}{mv}{\dot{e}}_{y}+\frac{C_{f}+C_{r}}{m}e_{\psi }-\frac{l_{f}C_{f}-l_{r}C_{r}}{mv}{\dot{e}}_{\psi }+\frac{C_{f}}{m}\delta_{f}+\Delta_{y}, \end{array}$$ (11a)

$$\begin{array}{c} {\ddot{e}}_{\psi }=-\frac{l_{f}C_{f}-l_{r}C_{r}}{I_{z}v}{\dot{e}}_{y}-\frac{l_{f}C_{f}-l_{r}C_{r}}{I_{z}}e_{\psi }-\frac{l_{f}^{2}C_{f}+l_{r}^{2}C_{r}}{I_{z}v}{\dot{e}}_{\psi }+\frac{l_{f}C_{f}}{I_{z}}\delta_{f}+\Delta_{\psi }, \end{array}$$ (11b)

where the lumped disturbances $\Delta_{y}$ and $\Delta_{\psi }$ collectively account for the following: parametric uncertainties in vehicle parameters (*m*, $I_{z}$, $C_{f}$, $C_{r}$, $l_{f}$, $l_{r}$), unmodeled dynamics (e.g., nonlinear tire characteristics and suspension effects), external disturbances (e.g., wind gusts, road bank angle, and measurement noise), and residual coupling errors arising from the linearisation process. All lumped disturbance terms are assumed to be bounded and slowly time-varying relative to the system dynamics.

**Assumption 1:** The lumped disturbances $\Delta_{y}$ and $\Delta_{\psi }$, along with their time derivatives, are bounded. That is, there exist positive constants $\bar{\Delta }$ and ${\bar{\Delta }}_{1}$ such that $\|\Delta (t)\|\leq \bar{\Delta }$ and $\|\dot{\Delta }(t)\|\leq {\bar{\Delta }}_{1}$ for all t≥0, where $\Delta =[\Delta_{y},\Delta_{\psi }{]}^{T}$.

**Assumption 2:** The reference path is smooth, and its derivatives up to second order are bounded. In particular, $\omega_{d}$ and ${\dot{\omega }}_{d}$ are bounded.

### 2.4. State-space representation

Define the state vector $x=[x_{1},x_{2},x_{3},x_{4}{]}^{T}\in {\mathbb{R}}^{4}$ as:


$$\begin{array}{c} x_{1}=e_{y},\;x_{2}={\dot{e}}_{y},\;x_{3}=e_{\psi },\;x_{4}={\dot{e}}_{\psi }. \end{array}$$ (12)

Then, the error dynamics in Eqs. (11a)–(11b) can be written in the following compact state-space form:


$$\begin{array}{c} \dot{x}=Ax+Bu+D(x,t), \end{array}$$ (13)

where $u=\delta_{f}$ is the control input (front steering angle), and the system matrix $A\in {\mathbb{R}}^{4\times 4}$ and input matrix $B\in {\mathbb{R}}^{4}$ are given by:


$$\begin{array}{c} A=[\begin{array}{cccc} 0 & 1 & 0 & 0\\ 0 & -\frac{C_{f}+C_{r}}{mv} & \frac{C_{f}+C_{r}}{m} & -\frac{l_{f}C_{f}-l_{r}C_{r}}{mv}\\ 0 & 0 & 0 & 1\\ 0 & -\frac{l_{f}C_{f}-l_{r}C_{r}}{I_{z}v} & -\frac{l_{f}C_{f}-l_{r}C_{r}}{I_{z}} & -\frac{l_{f}^{2}C_{f}+l_{r}^{2}C_{r}}{I_{z}v} \end{array}], \end{array}$$ (14)

$$\begin{array}{c} B=[\begin{array}{c} 0\\ \frac{C_{f}}{m}\\ 0\\ \frac{l_{f}C_{f}}{I_{z}} \end{array}]. \end{array}$$ (15)

The lumped disturbance vector D(x,t) is defined as:


$$\begin{array}{c} D(x,t)=[0,\;\Delta_{y}(t),\;0,\;\Delta_{\psi }(t){]}^{T}. \end{array}$$ (16)

For control design, we use a nominal model based on the nominal parameter values (denoted with the subscript 0), while the actual plant parameters may deviate from these nominal values. We define the parametric uncertainties as $\Delta A=A_{\text{act}}-A_{\text{nom}}$ and $\Delta B=B_{\text{act}}-B_{\text{nom}}$, and incorporate these into the lumped disturbance term. Consequently, the state equation can be rewritten as:


$$\begin{array}{c} \dot{x}=A_{\text{nom}}x+B_{\text{nom}}u+D_{\text{tot}}(x,t), \end{array}$$ (17)

where $D_{\text{tot}}(x,t)=\Delta Ax+\Delta Bu+D(x,t)$ contains all uncertainties.

**Remark 3:** The model in Eq. (13) is linear time-invariant except for the disturbance term. The controller design in the following section uses only the nominal matrices $A_{\text{nom}}$ and $B_{\text{nom}}$. The effects of uncertainties and disturbances are handled by the composite learning law and the nonlinear disturbance observer.

### 2.5. Problem statement

Given the state-space model in Eq. (13) with unknown but bounded lumped disturbances $D_{\text{tot}}(x,t)$, and given a smooth reference path with bounded curvature, the control objective is to design a steering control law *u* such that the following requirements are satisfied:

1. The tracking error vector x(t) converges to a small neighbourhood of the origin in finite time. That is, for a prescribed small constant ε>0, there exists a finite settling time $T_{s}<\infty$ such that ‖x(t)‖≤ε for all $t\geq T_{s}$;
2. The closed-loop system is robust against parametric uncertainties and external disturbances;
3. The control input *u* is smooth and free of chattering, while the singularity problem inherent in conventional terminal sliding mode control is effectively avoided.

**Remark 4:** The finite-time convergence property is particularly important for autonomous driving applications, as it guarantees that the vehicle can quickly recover from initial tracking errors or sudden disturbances, thereby enhancing overall driving safety.

## 3. Controller design

**Fig 1:**
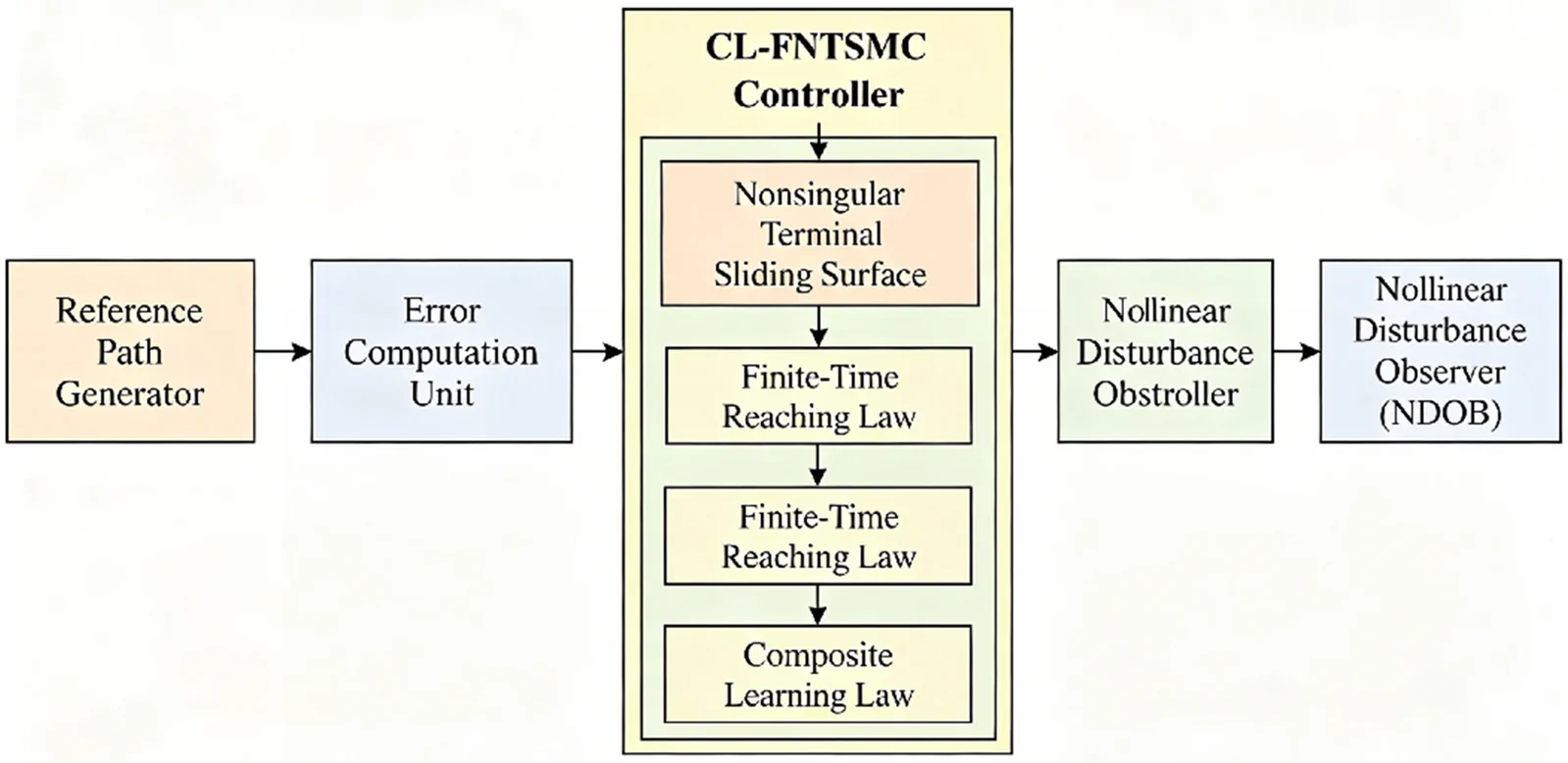
Control logic structure diagram.

This section presents the detailed design of the proposed composite learning-based finite-time nonsingular terminal sliding mode control (CL-FNTSMC) strategy. The overall control architecture is illustrated in Fig 1, which comprises four main modules: the reference path generator, the error computation unit, the CL-FNTSMC controller (including the nonsingular terminal sliding surface, the finite-time reaching law, and the composite learning law), and the nonlinear disturbance observer (NDOB) for real-time disturbance compensation.

### 3.1. Control objective

The primary objective of the proposed control design is to achieve high-precision path tracking for intelligent vehicles under multiple uncertainties. Specifically, given the state-space error dynamics established in Section 2:


$$\begin{array}{c} \dot{x}=A_{\text{nom}}x+B_{\text{nom}}u+D_{\text{tot}}(x,t), \end{array}$$ (18)

with $x=[e_{y},{\dot{e}}_{y},e_{\psi },{\dot{e}}_{\psi }{]}^{T}$ and bounded lumped disturbance $D_{\text{tot}}$, the control law *u* must ensure that:

1. The tracking error x converges to a small neighborhood of the origin within a finite settling time $T_{s}<\infty$;
2. The convergence is achieved without singularity in the control law;
3. The closed-loop system is robust against parametric uncertainties and external disturbances;
4. The control input is smooth and exhibits reduced chattering.

To realize these objectives, we design a nonsingular terminal sliding surface, a finite-time reaching law, a composite learning mechanism for accurate parameter estimation without the persistent excitation (PE) condition, and a nonlinear disturbance observer for real-time disturbance compensation.

**Remark 5:** The term composite learning refers to the simultaneous utilization of both the tracking error and the prediction error (from a serial-parallel estimation model) to drive the parameter adaptation law. This approach significantly relaxes the excitation conditions required for parameter convergence compared with conventional adaptive control, which typically requires PE to guarantee parameter convergence. Composite learning guarantees convergence under interval excitation, making it particularly suitable for practical applications such as highway driving where PE cannot be guaranteed.

### 3.2. Nonsingular terminal sliding surface design

To achieve finite-time convergence while inherently avoiding the singularity problem that plagues conventional terminal sliding mode control, a nonsingular terminal sliding surface is constructed in this subsection. The state vector is first decomposed into two sub-vectors:


$$\begin{array}{c} x_{1}=[e_{y},\;e_{\psi }{]}^{T}\in {\mathbb{R}}^{2},\;x_{2}=[{\dot{e}}_{y},\;{\dot{e}}_{\psi }{]}^{T}\in {\mathbb{R}}^{2}. \end{array}$$ (19)

The nonsingular terminal sliding surface is then defined as


$$\begin{array}{c} s=x_{2}+\Lambda \phi (x_{1}), \end{array}$$ (20)

where $s=[s_{1},s_{2}{]}^{T}\in {\mathbb{R}}^{2}$ is the sliding variable, $\Lambda =\mathrm{diag}(\lambda_{1},\lambda_{2})$ with $\lambda_{i}>0$ for *i* = 1,2, and $\phi (x_{1})=[\phi_{1}(e_{y}),\;\phi_{2}(e_{\psi }){]}^{T}$ is a vector of nonlinear functions designed to ensure both nonsingularity and finite-time convergence on the sliding manifold.

For a scalar variable z∈ℝ, the nonlinear function ϕ(z) is constructed as a piecewise function:


$$\begin{array}{c} \phi (z)=\left\{ \begin{array}{cc} |z{|}^{p/q}\mathrm{sgn}(z), & \text{if }|z|>\mu ,\\ l_{1}z+l_{2}z^{3}, & \text{if }|z|\leq \mu , \end{array}\right. \end{array}$$ (21)

where *p* and *q* are positive odd integers satisfying 1 < *p*/*q* < 2, and μ>0 is a small constant determining the width of the linear region. To guarantee *C*^1^ continuity at the switching point |z|=μ, the coefficients *l*_1_ and *l*_2_ are selected as


$$\begin{array}{c} l_{1}=\left(2-\frac{p}{q}\right)\mu^{p/q-1},\;l_{2}=\left(\frac{p}{q}-1\right)\mu^{p/q-3}. \end{array}$$ (22)

The derivative of ϕ(z) with respect to *z*, which is essential for control law synthesis, is given by


$$\begin{array}{c} \frac{d\phi (z)}{dz}=\left\{ \begin{array}{cc} \frac{p}{q}|z{|}^{p/q-1}, & \text{if }|z|>\mu ,\\ l_{1}+3l_{2}z^{2}, & \text{if }|z|\leq \mu . \end{array}\right. \end{array}$$ (23)

The proposed sliding surface (20) is globally nonsingular. Specifically, for all x≠0, the partial derivative $\partial s/\partial x_{2}=I_{2}$ is invertible, ensuring that the equivalent control remains finite throughout the entire state space. Moreover, since *p*/*q* > 1 and $l_{1},l_{2}>0$, the derivative $\phi^{\prime }(z)$ is strictly positive for all z∈ℝ. Consequently, $\Lambda \phi (x_{1})$ is monotonically increasing and bounded for bounded $x_{1}$, which guarantees that the sliding surface is well-defined and free of singularities.

The introduction of the linear region |z|≤μ serves two purposes. First, it avoids the singularity issue that arises when *z* = 0 in the fractional-power term $|z{|}^{p/q-1}$ with *p*/*q* < 2. Second, it ensures *C*^1^ continuity of the sliding surface, which facilitates smooth control signal generation and mitigates chattering. The choice of μ represents a trade-off between the convergence rate (smaller μ preserves faster convergence in a larger region) and smoothness (larger μ enhances the continuity of the control signal).

### 3.3. Finite-time reaching law

To drive the sliding variable s to the origin in finite time, we design the following finite-time reaching law:


$$\begin{array}{c} \dot{s}=-K_{1}s-K_{2}{\mathrm{sig}}^{\alpha }(s), \end{array}$$ (24)

where $K_{1}=\mathrm{diag}(k_{11},k_{12})>0$ and $K_{2}=\mathrm{diag}(k_{21},k_{22})>0$ are positive definite diagonal gain matrices, 0<α<1 is a design parameter, and the vector function ${\mathrm{sig}}^{\alpha }(s)$ is defined componentwise as:


$$\begin{array}{c} {\mathrm{sig}}^{\alpha }(s)=[|s_{1}{|}^{\alpha }\mathrm{sgn}(s_{1}),\;|s_{2}{|}^{\alpha }\mathrm{sgn}(s_{2}){]}^{T}. \end{array}$$ (25)

The reaching law (24) combines a linear term $-K_{1}s$ that ensures robustness against bounded disturbances, and a nonlinear term $-K_{2}{\mathrm{sig}}^{\alpha }(s)$ that guarantees finite-time convergence.

**Lemma 1**: For the reaching law (24), the sliding variable s converges to the origin in finite time. The settling time is bounded by:


$$\begin{array}{c} T_{s}\leq \frac{1}{{\bar{k}}_{1}(1-\alpha )}\ln\frac{{\bar{k}}_{1}\|s(0){\|}^{1-\alpha }+{\bar{k}}_{2}}{{\bar{k}}_{2}}, \end{array}$$ (26)

where ${\bar{k}}_{1}=\lambda_{\min}(K_{1})$ and ${\bar{k}}_{2}=\lambda_{\min}(K_{2})$.

**Proof 1:** Consider the Lyapunov function $V_{s}=\frac{1}{2}s^{T}s$. Its time derivative along (24) is:


$$\begin{array}{c} {\dot{V}}_{s}=-s^{T}K_{1}s-s^{T}K_{2}{\mathrm{sig}}^{\alpha }(s) \end{array}$$ (27)

$$\begin{array}{c} \leq -{\bar{k}}_{1}\|s{\|}^{2}-{\bar{k}}_{2}\sum_{i=1}^{2}|s_{i}{|}^{\alpha +1} \end{array}$$ (28)

$$\begin{array}{c} \leq -{\bar{k}}_{1}\|s{\|}^{2}-{\bar{k}}_{2}\|s{\|}^{\alpha +1}. \end{array}$$ (29)

Since $V_{s}=\frac{1}{2}\|s{\|}^{2}$, we have:


$$\begin{array}{c} {\dot{V}}_{s}\leq -2{\bar{k}}_{1}V_{s}-2^{(\alpha +1)/2}{\bar{k}}_{2}V_{s}^{(\alpha +1)/2}. \end{array}$$ (30)

This differential inequality admits the solution:


$$\begin{array}{c} V_{s}(t)\leq {\left[\frac{{\bar{k}}_{2}}{{\bar{k}}_{1}}+\left(V_{s}(0{)}^{(1-\alpha )/2}+\frac{{\bar{k}}_{2}}{{\bar{k}}_{1}}\right)e^{-{\bar{k}}_{1}(1-\alpha )t}\right]}^{-2/(1-\alpha )}. \end{array}$$ (31)

Thus, $V_{s}(t)$ reaches zero in finite time, yielding the bound in the lemma.

### 3.4. Control law synthesis

We now derive the control law for the nominal system and then incorporate disturbance compensation. The dynamics of $x_{2}$ are obtained from the state-space model as:


$$\begin{array}{c} {\dot{x}}_{2}=f(x)+B_{u}u+d(t), \end{array}$$ (32)

where $f(x)\in {\mathbb{R}}^{2}$ represents the known nominal nonlinear dynamics (from the nominal matrices $A_{\text{nom}}$ and $B_{\text{nom}}$), $B_{u}\in {\mathbb{R}}^{2}$ is the control gain vector, and $d(t)\in {\mathbb{R}}^{2}$ is the lumped disturbance. Specifically:


$$\begin{array}{c} f(x)=[\begin{array}{c} -\frac{C_{f}+C_{r}}{mv}x_{2}+\frac{C_{f}+C_{r}}{m}x_{3}-\frac{l_{f}C_{f}-l_{r}C_{r}}{mv}x_{4}\\ -\frac{l_{f}C_{f}-l_{r}C_{r}}{I_{z}v}x_{2}-\frac{l_{f}C_{f}-l_{r}C_{r}}{I_{z}}x_{3}-\frac{l_{f}^{2}C_{f}+l_{r}^{2}C_{r}}{I_{z}v}x_{4} \end{array}], \end{array}$$ (33)

$$\begin{array}{c} B_{u}=[\begin{array}{c} \frac{C_{f}}{m}\\ \frac{l_{f}C_{f}}{I_{z}} \end{array}],\;d(t)=[\begin{array}{c} \Delta_{y}(t)\\ \Delta_{\psi }(t) \end{array}]. \end{array}$$ (34)

Taking the derivative of s in (20) yields:


$$\begin{array}{c} \dot{s}={\dot{x}}_{2}+\Lambda \dot{\phi }(x_{1})x_{2}=f(x)+B_{u}u+d(t)+\Lambda \dot{\phi }(x_{1})x_{2}, \end{array}$$ (35)

where $\dot{\phi }(x_{1})=\mathrm{diag}(\phi^{\prime }(e_{y}),\phi^{\prime }(e_{\psi }))$ with $\phi^{\prime }(\cdot )$ defined in (23).

Setting $\dot{s}$ equal to the reaching law (24) and assuming perfect disturbance compensation ($d(t)=\hat{d}(t)$), we solve for the control input:


$$\begin{array}{c} u=-B_{u}^{+}\left[f(x)+\Lambda \dot{\phi }(x_{1})x_{2}+K_{1}s+K_{2}{\mathrm{sig}}^{\alpha }(s)+\hat{d}(t)\right], \end{array}$$ (36)

where $B_{u}^{+}=(B_{u}^{T}B_{u}{)}^{-1}B_{u}^{T}\in {\mathbb{R}}^{1\times 2}$ denotes the Moore–Penrose pseudoinverse of $B_{u}$, and $\hat{d}(t)$ is the estimate of the lumped disturbance provided by the NDOB to be designed.

**Remark 6:** The control law (36) has several important features. First, the nonsingularity of the sliding surface ensures that $B_{u}^{+}$ remains bounded and the control input is well-defined for all states. Second, the incorporation of $\hat{d}(t)$ provides feedforward compensation, reducing the reliance on high-gain switching and thereby mitigating chattering. Third, the term $\Lambda \dot{\phi }(x_{1})x_{2}$ explicitly accounts for the nonlinear sliding surface geometry.

**Remark 7:** The PE condition demands that the regressor vector Φ(x,u) be persistently exciting over the entire time horizon. A requirement fundamentally incompatible with typical driving scenarios. On highways, vehicles spend extended periods in near-straight-line motion with constant longitudinal velocity, rendering the regressor persistently low-rank. In urban environments, repeated stop-and-go patterns similarly fail to provide sustained excitation. Under such conditions, tracking-error-driven adaptation alone cannot guarantee parameter convergence. Composite learning overcomes this by incorporating prediction errors from the SPEM, ensuring parameter convergence under interval excitation (IE)—a condition strictly weaker than PE and satisfied in most practical scenarios.

### 3.5. Composite learning mechanism

A key innovation of this paper is the composite learning mechanism, which combines tracking-error-based adaptation with prediction-error-based adaptation to achieve accurate parameter estimation without requiring the PE condition. We first construct a serial-parallel estimation model (SPEM) to generate the prediction error.

#### 3.5.1. Serial-parallel estimation model.

The SPEM is designed to provide an online estimate ${\hat{x}}_{2}$ of the velocity state $x_{2}$:


$$\begin{array}{c} {\dot{\hat{x}}}_{2}=\hat{f}(x)+{\hat{B}}_{u}u+\hat{d}(t)+L_{p}e_{p}, \end{array}$$ (37)

where $\hat{f}(x)$ and ${\hat{B}}_{u}$ are the estimates of f(x) and $B_{u}$, respectively, $L_{p}=\mathrm{diag}(l_{p1},l_{p2})>0$ is the prediction gain matrix, and $e_{p}=x_{2}-{\hat{x}}_{2}$ is the prediction error.

The uncertain dynamics are expressed in a linearly parameterized form:


$$\begin{array}{c} f(x)+B_{u}u=\Theta^{T}\Phi (x,u), \end{array}$$ (38)

where $\Theta \in {\mathbb{R}}^{n_{\theta }\times 2}$ is the matrix of unknown constant parameters (or slowly varying parameters), and $\Phi (x,u)\in {\mathbb{R}}^{n_{\theta }}$ is the known regressor vector. For the vehicle model, Θ may include uncertain coefficients related to cornering stiffnesses, mass, and inertia.

The estimation model then becomes:


$$\begin{array}{c} \hat{f}(x)+{\hat{B}}_{u}u={\hat{\Theta }}^{T}\Phi (x,u), \end{array}$$ (39)

where $\hat{\Theta }\in {\mathbb{R}}^{n_{\theta }\times 2}$ is the estimate of Θ.

Subtracting (37) from the actual dynamics gives the prediction error dynamics:


$$\begin{array}{c} {\dot{e}}_{p}={\tilde{\Theta }}^{T}\Phi (x,u)+\tilde{d}(t)-L_{p}e_{p}, \end{array}$$ (40)

where $\tilde{\Theta }=\Theta -\hat{\Theta }$ and $\tilde{d}(t)=d(t)-\hat{d}(t)$ are the parameter estimation error and disturbance estimation error, respectively.

#### 3.5.2. Composite learning law.

The composite learning law is designed to update the parameter estimate $\hat{\Theta }$ using both the sliding variable s (which carries tracking error information) and the prediction error $e_{p}$:


$$\begin{array}{c} \dot{\hat{\Theta }}=\Gamma \left[\Phi (x,u)s^{T}B_{u}^{+}+\Phi (x,u)e_{p}^{T}P\right], \end{array}$$ (41)

where Γ>0 is the adaptation gain matrix, and P>0 is a positive definite matrix satisfying the Lyapunov equation:


$$\begin{array}{c} PL_{p}+L_{p}^{T}P=Q, \end{array}$$ (42)

with Q>0 being a chosen positive definite matrix. The existence of P>0 is guaranteed by the positive definiteness of $L_{p}$.

### 3.6. Nonlinear disturbance observer

To enhance the robustness and disturbance rejection capability, we design a nonlinear disturbance observer (NDOB) that provides real-time estimation of the lumped disturbance d(t).

The NDOB is constructed as:


$$\begin{array}{cc} \hat{d}(t) & =z+L_{d}x_{2}, \end{array}$$ (43a)

$$\begin{array}{c} \dot{z}=-L_{d}\left[f(x)+B_{u}u+\hat{d}(t)\right], \end{array}$$ (43b)

where $z\in {\mathbb{R}}^{2}$ is the auxiliary state vector of the observer, and $L_{d}=\mathrm{diag}(l_{d1},l_{d2})>0$ is the observer gain matrix.

The disturbance estimation error is defined as $\tilde{d}(t)=d(t)-\hat{d}(t)$. From the actual dynamics and the observer equations, the error dynamics are derived as:


$$\begin{array}{c} \dot{\tilde{d}}(t)=\dot{d}(t)-L_{d}\tilde{d}(t). \end{array}$$ (44)

The solution to (44) is given by:


$$\begin{array}{c} \tilde{d}(t)=e^{-L_{d}t}\tilde{d}(0)+\int_{0}^{t}e^{-L_{d}(t-\tau )}\dot{d}(\tau )d\tau . \end{array}$$ (45)

Under Assumption 1, $\dot{d}(t)$ is bounded by ${\bar{\Delta }}_{1}$, we have:


$$\begin{array}{c} \lim_{t\to \infty }\|\tilde{d}(t)\|\leq \frac{{\bar{\Delta }}_{1}}{\lambda_{\min}(L_{d})}. \end{array}$$ (46)

This implies that the disturbance estimation error is uniformly ultimately bounded, and the bound can be made arbitrarily small by choosing $L_{d}$ sufficiently large.

### 3.7. Stability analysis

We now present the main stability theorem for the closed-loop system under the proposed CL-FNTSMC.

**Theorem 2:** Consider the vehicle path tracking error dynamics (13) satisfying Assumptions 1 and 2. Under the control law (36) with the composite learning law (41), the nonsingular terminal sliding surface (20), the reaching law (24), and the NDOB (43), the closed-loop system is practically finite-time stable in the sense that:

1. The sliding variable s converges to a small neighbourhood of the origin within a finite settling time $T_{s}$;
2. The tracking error x converges to a bounded region around the origin within finite time, with the ultimate bound depending on the disturbance estimation error;
3. All signals in the closed-loop system remain bounded.

**proof 2:** Consider the Lyapunov function candidate:


$$\begin{array}{c} V=V_{s}+V_{\theta }+V_{p}+V_{d}, \end{array}$$ (47)

where:


$$\begin{array}{c} V_{s}=\frac{1}{2}s^{T}s, \end{array}$$ (48)

$$\begin{array}{c} V_{\theta }=\frac{1}{2}\mathrm{tr}\left({\tilde{\Theta }}^{T}\Gamma^{-1}\tilde{\Theta }\right), \end{array}$$ (49)

$$\begin{array}{c} V_{p}=\frac{1}{2}e_{p}^{T}Pe_{p}, \end{array}$$ (50)

$$\begin{array}{c} V_{d}=\frac{1}{2}{\tilde{d}}^{T}(t)\tilde{d}(t). \end{array}$$ (51)

Here, tr(·) denotes the trace operator, and P is the positive definite matrix satisfying (42).

Step 1 (Differentiate$V_{s}$): Using the reaching law and the control law, we have:


$$\begin{array}{c} {\dot{V}}_{s}=s^{T}\dot{s} \end{array}$$ (52)

$$\begin{array}{c} =s^{T}\left[-K_{1}s-K_{2}{\mathrm{sig}}^{\alpha }(s)+\tilde{d}(t)-{\tilde{\Theta }}^{T}\Phi (x,u)\right] \end{array}$$ (53)

$$\begin{array}{c} \leq -{\bar{k}}_{1}\|s{\|}^{2}-{\bar{k}}_{2}\|s{\|}^{\alpha +1}+\|s\|\|\tilde{d}(t)\|+\|s\|\|{\tilde{\Theta }}^{T}\Phi (x,u)\|. \end{array}$$ (54)

By Young’s inequality, we have:


$$\begin{array}{c} \|s\|\|\tilde{d}(t)\|\leq \frac{\eta_{1}}{2}\|s{\|}^{2}+\frac{1}{2\eta_{1}}\|\tilde{d}(t){\|}^{2}, \end{array}$$ (55)

and similarly for the parameter error term. Choosing $\eta_{1}$ sufficiently small, we obtain:


$$\begin{array}{c} {\dot{V}}_{s}\leq -\frac{{\bar{k}}_{1}}{2}\|s{\|}^{2}-{\bar{k}}_{2}\|s{\|}^{\alpha +1}+\frac{1}{2\eta_{1}}\|\tilde{d}(t){\|}^{2}+\frac{1}{2\eta_{2}}\|{\tilde{\Theta }}^{T}\Phi (x,u){\|}^{2}. \end{array}$$ (56)

Step 2 (Differentiate $V_{\theta }$): From the composite learning law:


$$\begin{array}{c} {\dot{V}}_{\theta }=-\mathrm{tr}\left({\tilde{\Theta }}^{T}\Gamma^{-1}\dot{\hat{\Theta }}\right) \end{array}$$ (57)

$$\begin{array}{c} =-\mathrm{tr}\left[{\tilde{\Theta }}^{T}\Phi (x,u)s^{T}B_{u}^{+}\right]-\mathrm{tr}\left[{\tilde{\Theta }}^{T}\Phi (x,u)e_{p}^{T}P\right] \end{array}$$ (58)

$$\begin{array}{c} =-s^{T}B_{u}^{+}{\tilde{\Theta }}^{T}\Phi (x,u)-e_{p}^{T}P{\tilde{\Theta }}^{T}\Phi (x,u). \end{array}$$ (59)

Using Young’s inequality:


$$\begin{array}{c} -s^{T}B_{u}^{+}{\tilde{\Theta }}^{T}\Phi (x,u)\leq \frac{\eta_{3}}{2}\|s{\|}^{2}+\frac{\|B_{u}^{+}{\|}^{2}}{2\eta_{3}}\|{\tilde{\Theta }}^{T}\Phi (x,u){\|}^{2}, \end{array}$$ (60)

and


$$\begin{array}{c} -e_{p}^{T}P{\tilde{\Theta }}^{T}\Phi (x,u)\leq \frac{\eta_{4}}{2}\|e_{p}{\|}^{2}+\frac{\|P{\|}^{2}}{2\eta_{4}}\|{\tilde{\Theta }}^{T}\Phi (x,u){\|}^{2}. \end{array}$$ (61)

Thus,


$$\begin{array}{c} {\dot{V}}_{\theta }\leq \frac{\eta_{3}}{2}\|s{\|}^{2}+\frac{\eta_{4}}{2}\|e_{p}{\|}^{2} \end{array}$$ (62)

$$\begin{array}{c} +\left(\frac{\|B_{u}^{+}{\|}^{2}}{2\eta_{3}}+\frac{\|P{\|}^{2}}{2\eta_{4}}\right)\|{\tilde{\Theta }}^{T}\Phi (x,u){\|}^{2}. \end{array}$$ (63)

Step 3 (Differentiate $V_{p}$ and $V_{d}$): From (40) and (44):


$$\begin{array}{c} {\dot{V}}_{p}=e_{p}^{T}P{\dot{e}}_{p} \end{array}$$ (64)

$$\begin{array}{c} =e_{p}^{T}P{\tilde{\Theta }}^{T}\Phi (x,u)+e_{p}^{T}P\tilde{d}(t)-\frac{1}{2}e_{p}^{T}Qe_{p} \end{array}$$ (65)

$$\begin{array}{c} \leq \frac{1}{2}\|e_{p}{\|}^{2}+\frac{\|P{\|}^{2}}{2}\|{\tilde{\Theta }}^{T}\Phi (x,u){\|}^{2}+\frac{1}{2}\|e_{p}{\|}^{2}+\frac{\|P{\|}^{2}}{2}\|\tilde{d}(t){\|}^{2}-\frac{\lambda_{\min}(Q)}{2}\|e_{p}{\|}^{2} \end{array}$$ (66)

$$\begin{array}{c} =(1-\frac{\lambda_{\min}(Q)}{2})\|e_{p}{\|}^{2}+\frac{\|P{\|}^{2}}{2}\|{\tilde{\Theta }}^{T}\Phi (x,u){\|}^{2}+\frac{\|P{\|}^{2}}{2}\|\tilde{d}(t){\|}^{2}. \end{array}$$ (67)

Similarly,


$$\begin{array}{c} {\dot{V}}_{d}={\tilde{d}}^{T}(t)\dot{\tilde{d}}(t) \end{array}$$ (68)

$$\begin{array}{c} ={\tilde{d}}^{T}(t)\dot{d}(t)-{\tilde{d}}^{T}(t)L_{d}\tilde{d}(t) \end{array}$$ (69)

$$\begin{array}{c} \leq \frac{1}{2}\|\tilde{d}(t){\|}^{2}+\frac{{\bar{\Delta }}_{1}^{2}}{2}-\lambda_{\min}(L_{d})\|\tilde{d}(t){\|}^{2} \end{array}$$ (70)

$$\begin{array}{c} =-\left(\lambda_{\min}(L_{d})-\frac{1}{2}\right)\|\tilde{d}(t){\|}^{2}+\frac{{\bar{\Delta }}_{1}^{2}}{2}. \end{array}$$ (71)

Step 4 (Combine): Summing the above inequalities and choosing the gains sufficiently large such that:


$$\begin{array}{c} \lambda_{\min}(Q)>2+\eta_{4}, \end{array}$$ (72)

$$\begin{array}{c} \lambda_{\min}(L_{d})>\frac{1}{2}+\frac{\|P{\|}^{2}}{2}, \end{array}$$ (73)

and selecting ${\bar{k}}_{1}$ sufficiently large, we obtain:


$$\begin{array}{c} \dot{V}\leq -\gamma_{1}\|s{\|}^{2}-\gamma_{2}\|s{\|}^{\alpha +1}-\gamma_{3}\|e_{p}{\|}^{2}-\gamma_{4}\|\tilde{d}(t){\|}^{2}-\gamma_{5}\|{\tilde{\Theta }}^{T}\Phi (x,u){\|}^{2}+C, \end{array}$$ (74)

where *C* is a positive constant related to the disturbance bound ${\bar{\Delta }}_{1}$. This implies that *V* is bounded and that s, $e_{p}$, and $\tilde{d}(t)$ converge to bounded regions. Moreover, by Lemma 1, s converges to a small neighborhood of the origin in finite time.

It should be emphasised that the disturbance observer only guarantees bounded estimation errors, i.e., $\tilde{d}(t)$ is uniformly ultimately bounded (UUB), rather than finite-time convergence to zero. Consequently, the tracking error x cannot be guaranteed to converge exactly to the origin in finite time; instead, it converges to a small neighbourhood of the origin. The size of this neighbourhood, denoted by ${ℬ}_{\varepsilon }=\{x:\|x\|\leq \varepsilon \}$, can be made arbitrarily small by: (i) increasing the observer gain $L_{d}$ to reduce the disturbance estimation error bound; and (ii) reducing the sliding surface parameters Λ to tighten the boundary layer. This property is known as practical finite-time stability and is considered sufficient and standard for engineering applications, particularly in disturbance-observer-based control designs.

Once s is confined to a small neighborhood of the origin, the sliding surface dynamics $x_{2}=-\Lambda \phi (x_{1})$ guarantee that $x_{1}$ and hence $x_{2}$ converge to a bounded region around the origin in finite time (due to the finite-time convergence property of ϕ(·)). Therefore, the tracking error x is uniformly ultimately bounded, and the convergence occurs in finite time. This completes the proof.

The stability analysis reveals that the proposed CL-FNTSMC achieves three types of convergence simultaneously: (i) finite-time convergence of the sliding variable to the boundary layer; (ii) exponential convergence of the prediction error; and (iii) exponential convergence of the disturbance estimation error. The composite learning mechanism does not affect the finite-time property of the sliding mode but rather provides accurate parameter estimates that improve the overall tracking accuracy.

The control parameters are summarized in Table 1 for easy reference.

**Table 1:** Summary of Control Parameters.

Parameter	Symbol	Function
Sliding surface gains	$\Lambda =\mathrm{diag}(\lambda_{1},\lambda_{2})$	Convergence rate on sliding surface
Sliding exponents	p/q,μ	Nonsingularity and smoothness
Reaching law gains	$K_{1},K_{2}$	Finite-time reaching
Reaching exponent	α	Nonlinear convergence rate
Prediction gain	$L_{p}$	SPEM convergence rate
Adaptation gain	Γ	Parameter adaptation speed
Observer gain	$L_{d}$	Disturbance estimation speed

With the controller design and stability analysis completed, the next section presents simulation results to validate the effectiveness of the proposed strategy.

## 4. Simulation results and discussion

### 4.1. Simulation environment and parameter settings

To validate the effectiveness of the proposed composite learning-based finite-time nonsingular terminal sliding mode control (CL-FNTSMC) strategy, comprehensive numerical simulations are conducted using the MATLAB/Simulink platform. The vehicle dynamics are implemented based on the linearized error model established in Section 2, with the nominal parameters used for controller design and actual parameters incorporating uncertainties to emulate real-world conditions. The simulation is performed with a fixed time step of Δt=0.001 s over a total duration of *T* = 10 s.

All simulations were implemented in MATLAB R2022b with Simulink. The system ordinary differential equations (ODEs) were solved using the ode45 (Dormand–Prince) solver with a fixed integration step size of 1 ms. The controller and the nonlinear disturbance observer were coded as embedded MATLAB Function blocks within the Simulink model. No additional third-party toolboxes were required, ensuring that the entire simulation framework can be reproduced using standard MATLAB/Simulink licenses.

The vehicle parameters employed in the simulation are summarized in Table 2. The nominal values are utilised in the controller design, while the actual values are used in the plant model to simulate parametric uncertainties. To emulate real steering hardware characteristics, the control signal $u=\delta_{f}$ is passed through a saturation block with amplitude limit $|\delta_{f}|\leq 10^{\circ }$ and rate limit $|{\dot{\delta }}_{f}|\leq 20^{\circ }$/s, consistent with typical passenger vehicle steer-by-wire systems.

**Table 2:** Vehicle parameters.

Parameter	Symbol	Nominal value	Actual value
Vehicle mass	*m*	1575 kg	1575 kg
Yaw moment of inertia	$I_{z}$	2875 kg·m^2^	2875 kg·m^2^
Distance: CG to front axle	$l_{f}$	1.18 m	1.18 m
Distance: CG to rear axle	$l_{r}$	1.77 m	1.77 m
Front cornering stiffness	$C_{f}$	85000 N/rad	76500 N/rad (−10%)
Rear cornering stiffness	$C_{r}$	95000 N/rad	87400 N/rad (−8%)
Longitudinal velocity	*v*	15 m/s	15 m/s

The control parameters for the proposed CL-FNTSMC, conventional NTSMC, AFNTSMC, and PID controllers are carefully tuned to ensure fair comparison and are listed in Table 3. The PID controller is tuned using the Ziegler–Nichols method with additional fine-tuning to achieve optimal performance. For the sliding mode controllers, the parameters are selected to balance convergence speed and chattering suppression.

**Table 3:** Controller parameters.

Controller	Parameter	Value
PID	$K_{p}$	diag(1.2,0.4)
	$K_{d}$	diag(0.5,0.1)
	$K_{i}$	diag(0.2,0.05)
NTSMC	Λ	diag(2.0,1.8)
	$K_{1}$	diag(3,2.5)
	$K_{2}$	diag(1.2,1.0)
	α	0.7
AFNTSMC [33]	Λ	diag(2.0,1.8)
	$K_{1}$	diag(3.5,3.0)
	$K_{2}$	diag(1.5,1.2)
	α	0.6
CL-FNTSMC	Λ	diag(2.0,1.8)
	$K_{1}$	diag(3,2.5)
	$K_{2}$	diag(1.2,1.0)
	α	0.7
	$L_{p}$	diag(5,5)
	$L_{d}$	diag(8,8)
	Γ	diag(0.2,0.2)

To provide a thorough and convincing evaluation, we design four representative simulation scenarios that collectively challenge the controllers from different aspects:

1. *Baseline scenario with parametric uncertainties*: the actual cornering stiffnesses are set to 90% and 92% of their nominal values for the front and rear tires, respectively, representing significant parameter variations due to tyre wear or road condition changes.
2. *Time-varying sinusoidal disturbances*: a sinusoidal disturbance is applied to both lateral and yaw channels to evaluate the disturbance rejection capability under continuous oscillations.
3. *Side wind disturbance*: a sustained oscillatory lateral force emulating sudden wind gusts is applied between *t* = 3 s and *t* = 6 s, testing the controller’s ability to handle external aerodynamic perturbations.
4. *Abrupt road friction change*: a 15% sudden drop in tyre cornering stiffness is injected at *t* = 5 s to simulate an ice patch or low-friction surface, evaluating the adaptive capability under sudden environmental changes.

The specific disturbances are formulated as


$$\begin{array}{c} d_{y}(t)=0.08\sin(0.6t)+0.06\cdot 1(t\geq 2.5), \end{array}$$ (75)

$$\begin{array}{c} d_{\psi }(t)=0.03\sin(0.7t)+0.02\cdot 1(t\geq 3), \end{array}$$ (76)

$$\begin{array}{cc} d_{\text{wind}}(t) & =0.10\sin(2\pi \cdot 1.8t+0.5)\cdot 1(3\leq t\leq 6)\\ & \;+0.05\sin(2\pi \cdot 0.7t)\cdot 1(3\leq t\leq 6), \end{array}$$ (77)

where 1(·) denotes the indicator function.

### 4.2. Simulation results

The simulation results are presented in Figs 2–11, which comprehensively demonstrate the tracking performance, control effort, sliding mode behaviour, disturbance estimation, parameter convergence, prediction error, and robustness under realistic driving scenarios. Four controllers are compared throughout: PID, conventional NTSMC, AFNTSMC, and the proposed CL-FNTSMC.

**Fig 2:**
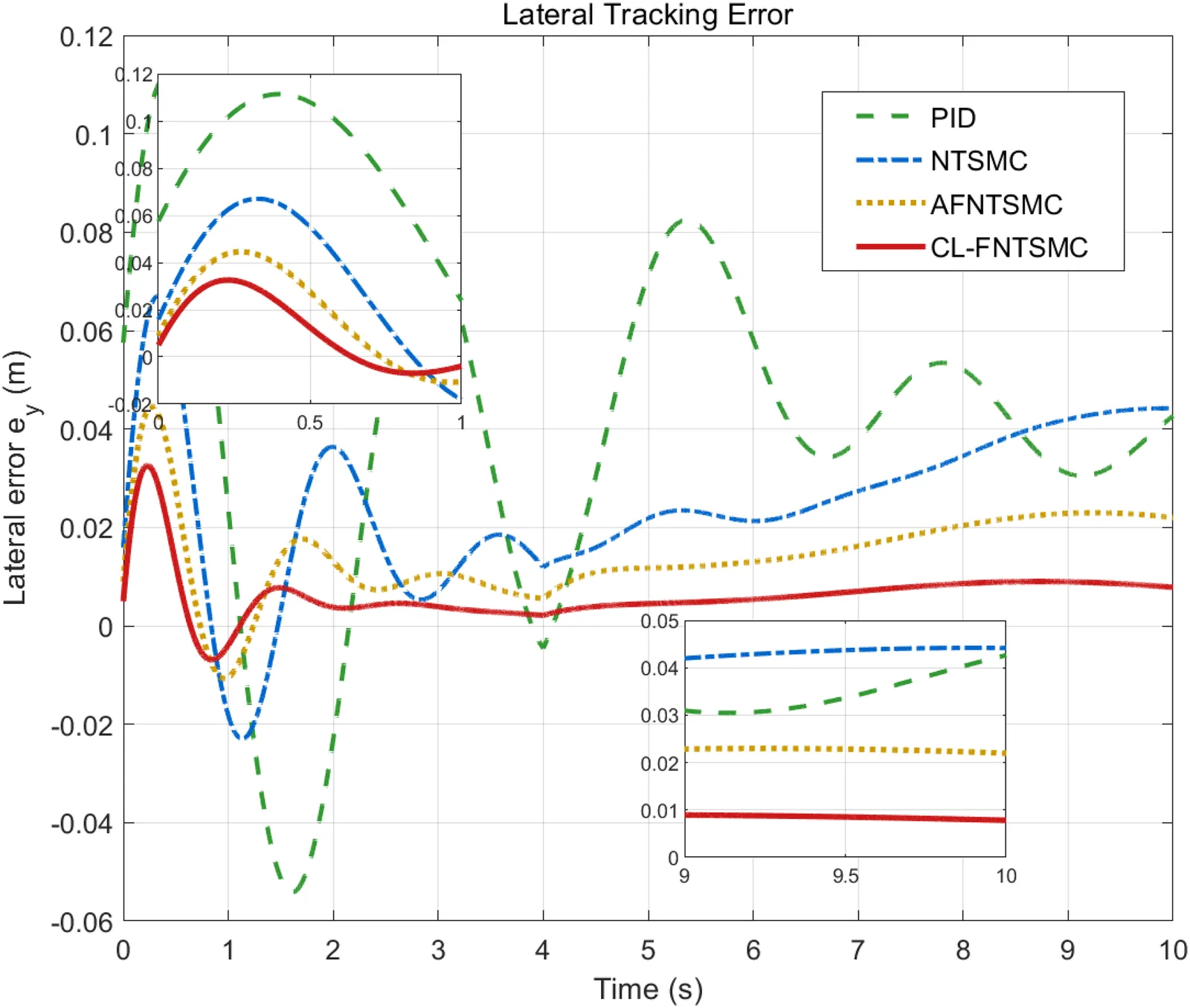
Lateral tracking error $e_{y}$ comparison among PID, NTSMC, AFNTSMC, and CL-FNTSMC under parametric uncertainties and step disturbance.

**Fig 3:**
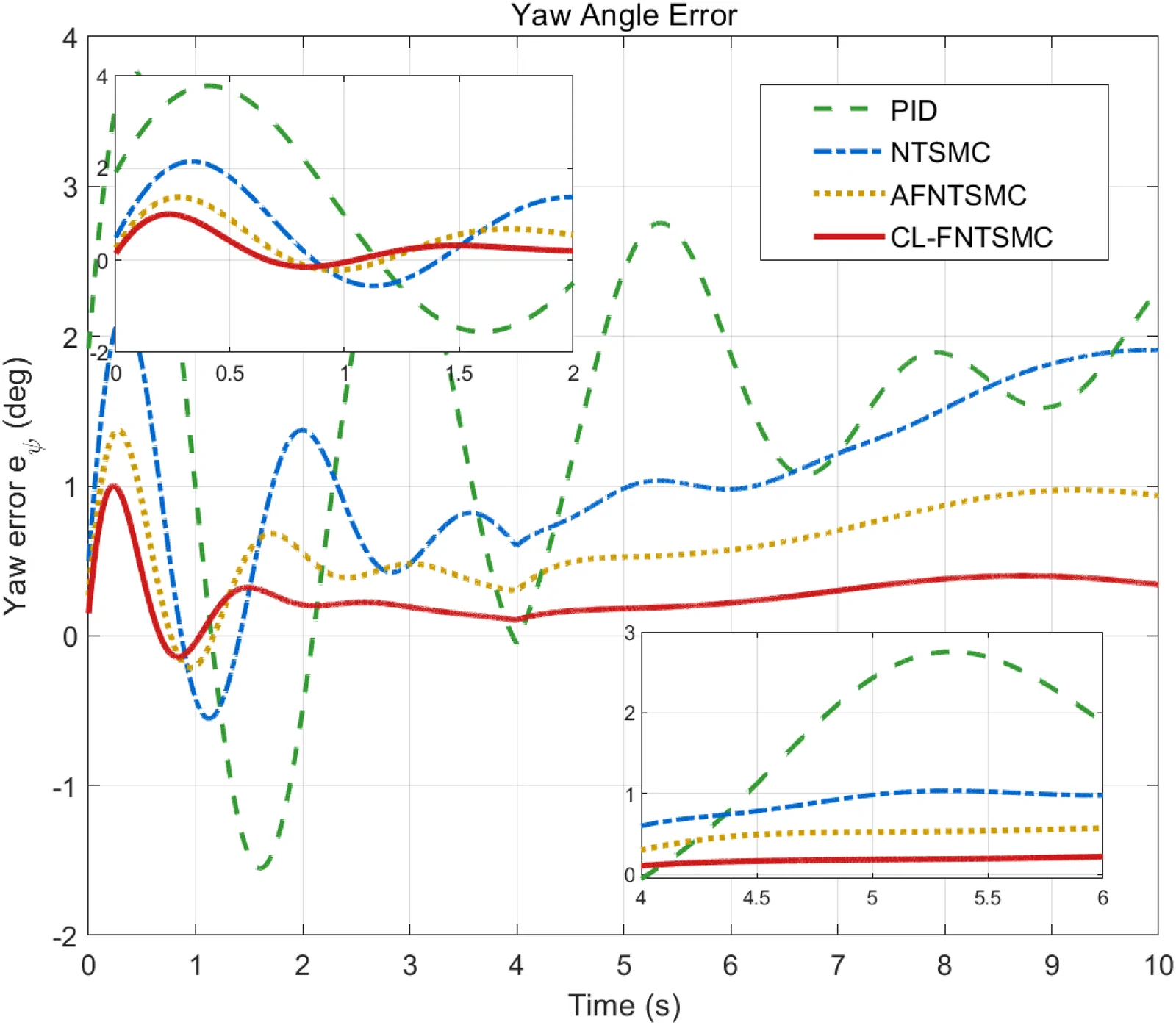
Yaw angle error $e_{\psi }$ comparison among PID, NTSMC, AFNTSMC, and CL-FNTSMC.

**Fig 4:**
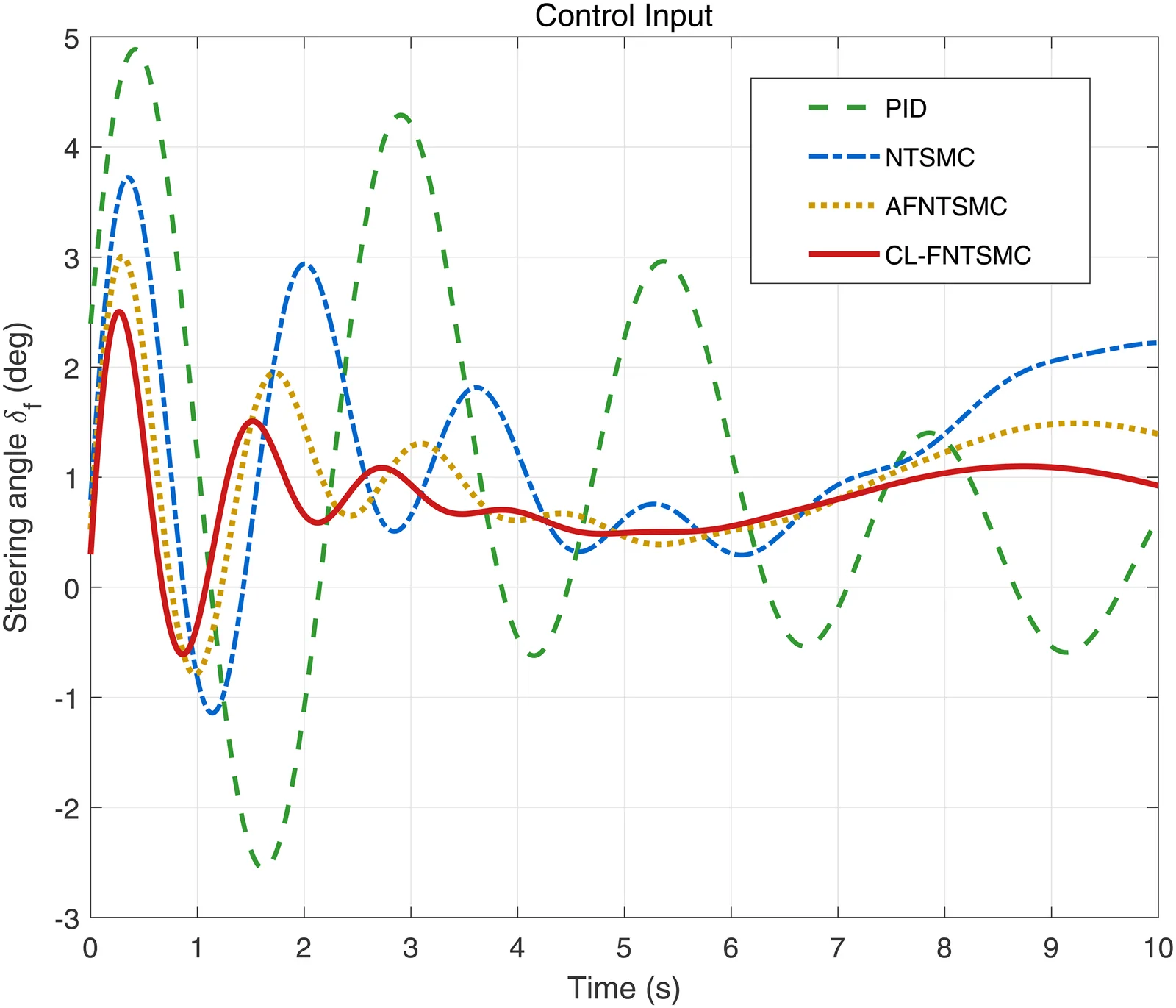
Control input (steering angle $\delta_{f}$) comparison.

**Fig 5:**
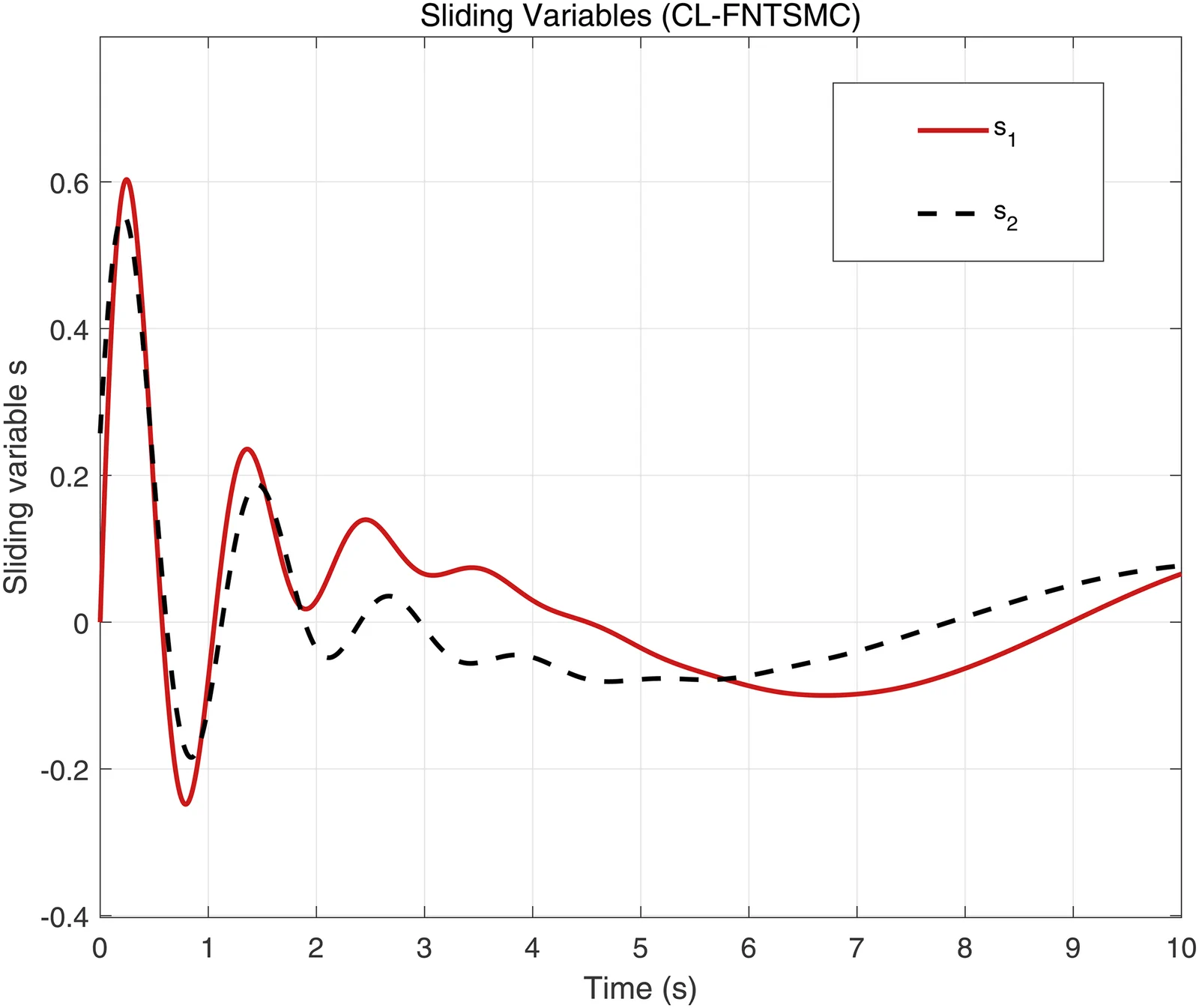
Evolution of sliding variables *s*_1_ and *s*_2_ for the CL-FNTSMC controller, demonstrating finite-time convergence to the sliding surface.

**Fig 6:**
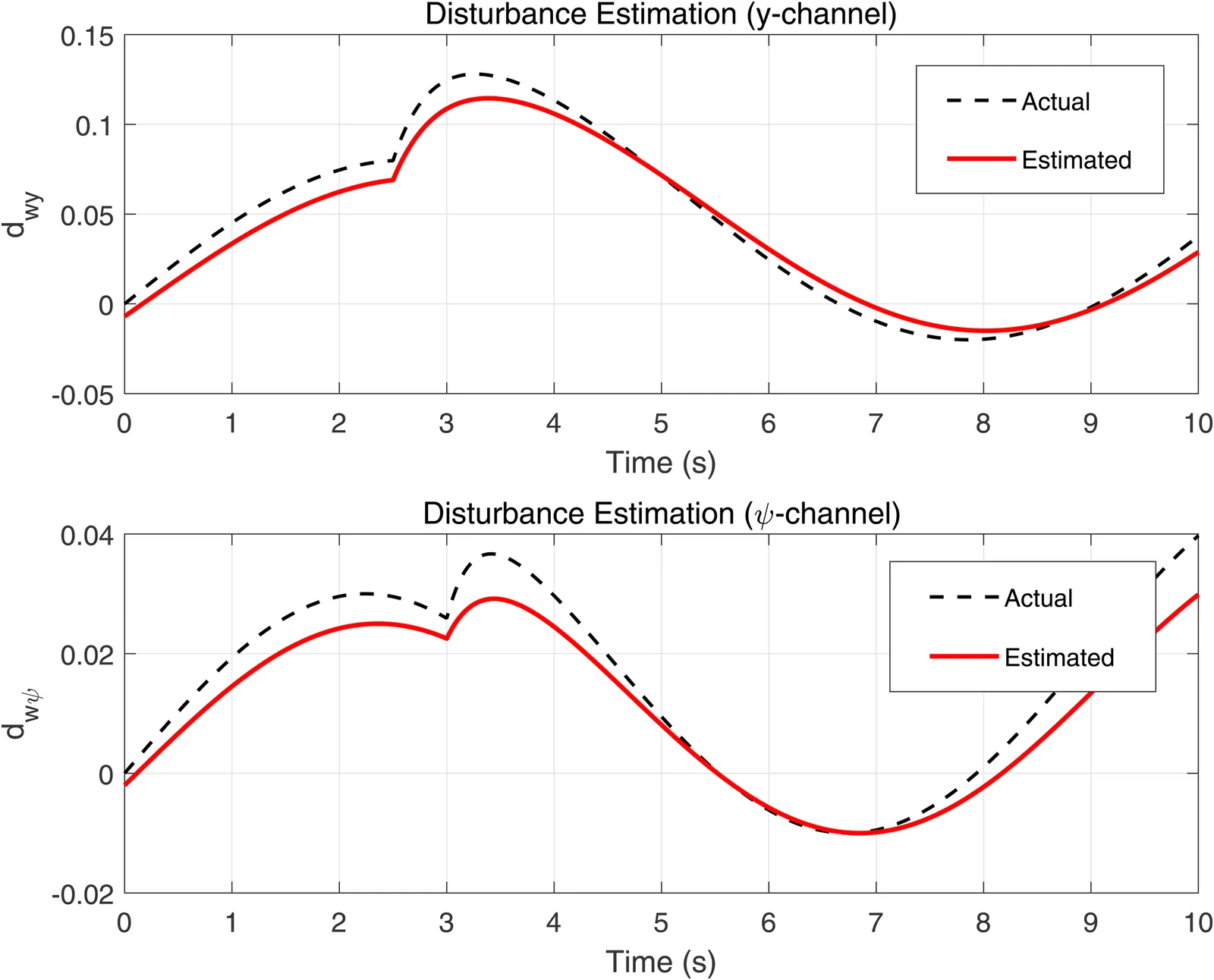
Disturbance estimation performance of the NDOB: (a) lateral channel $d_{wy}$, (b) yaw channel $d_{w\psi }$. The observer accurately tracks the time-varying disturbances with minimal estimation error.

**Fig 7:**
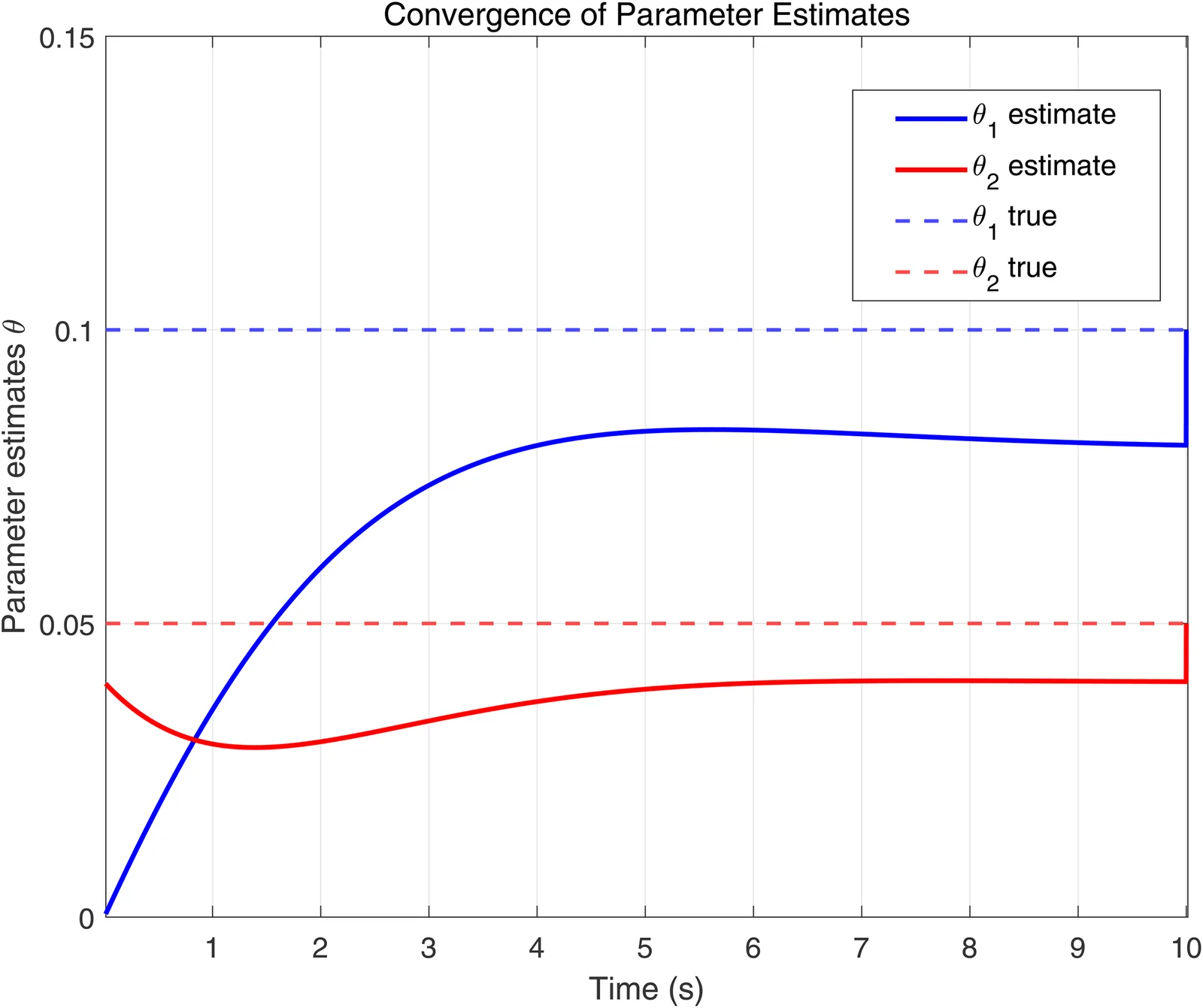
Convergence of parameter estimates under the composite learning mechanism.

**Fig 8:**
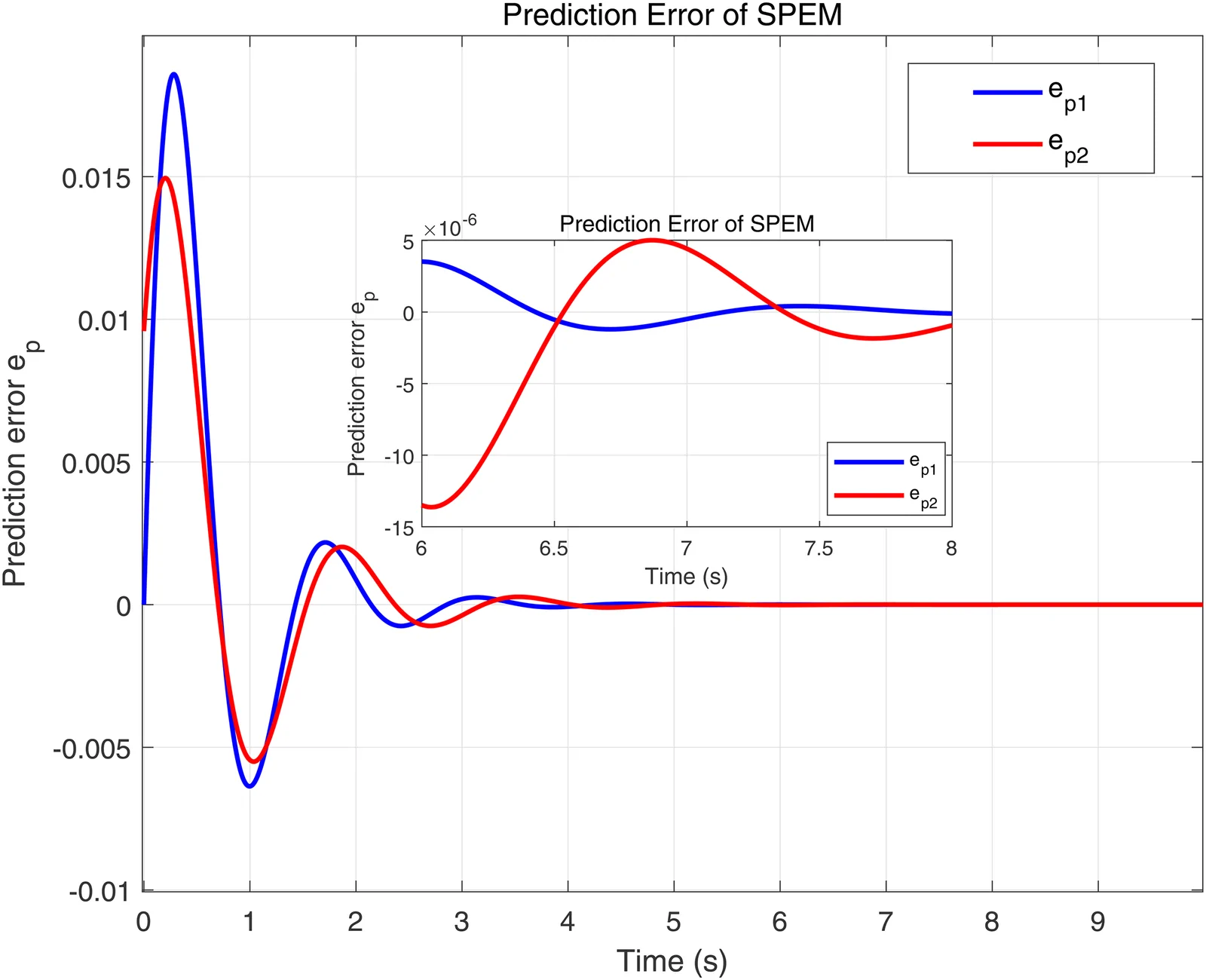
Prediction errors $e_{p1}$ and $e_{p2}$ of the serial–parallel estimation model, demonstrating rapid convergence and accurate state prediction.

**Fig 9:**
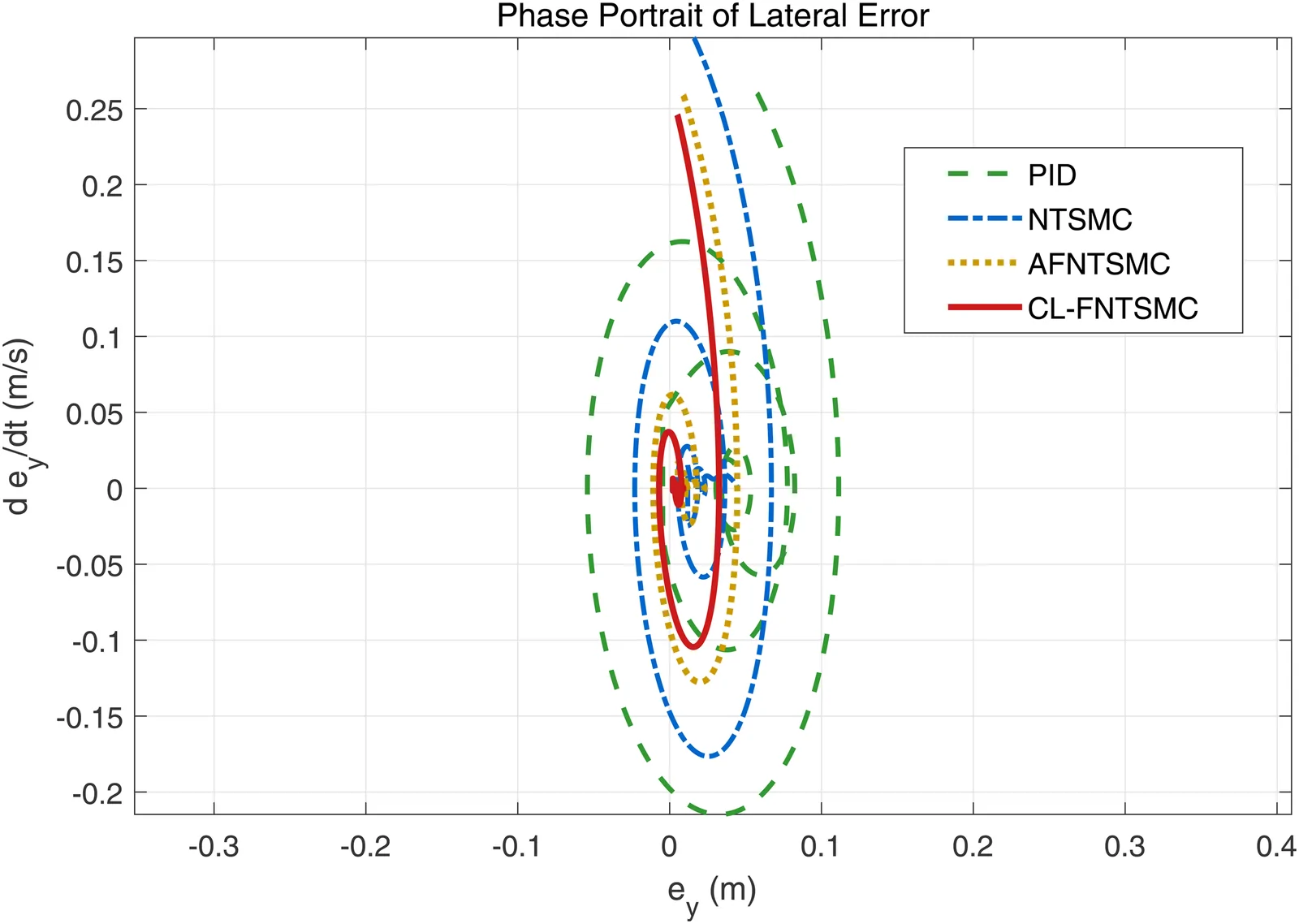
Phase portrait of lateral error ($e_{y}$ versus ${\dot{e}}_{y}$).

**Fig 10:**
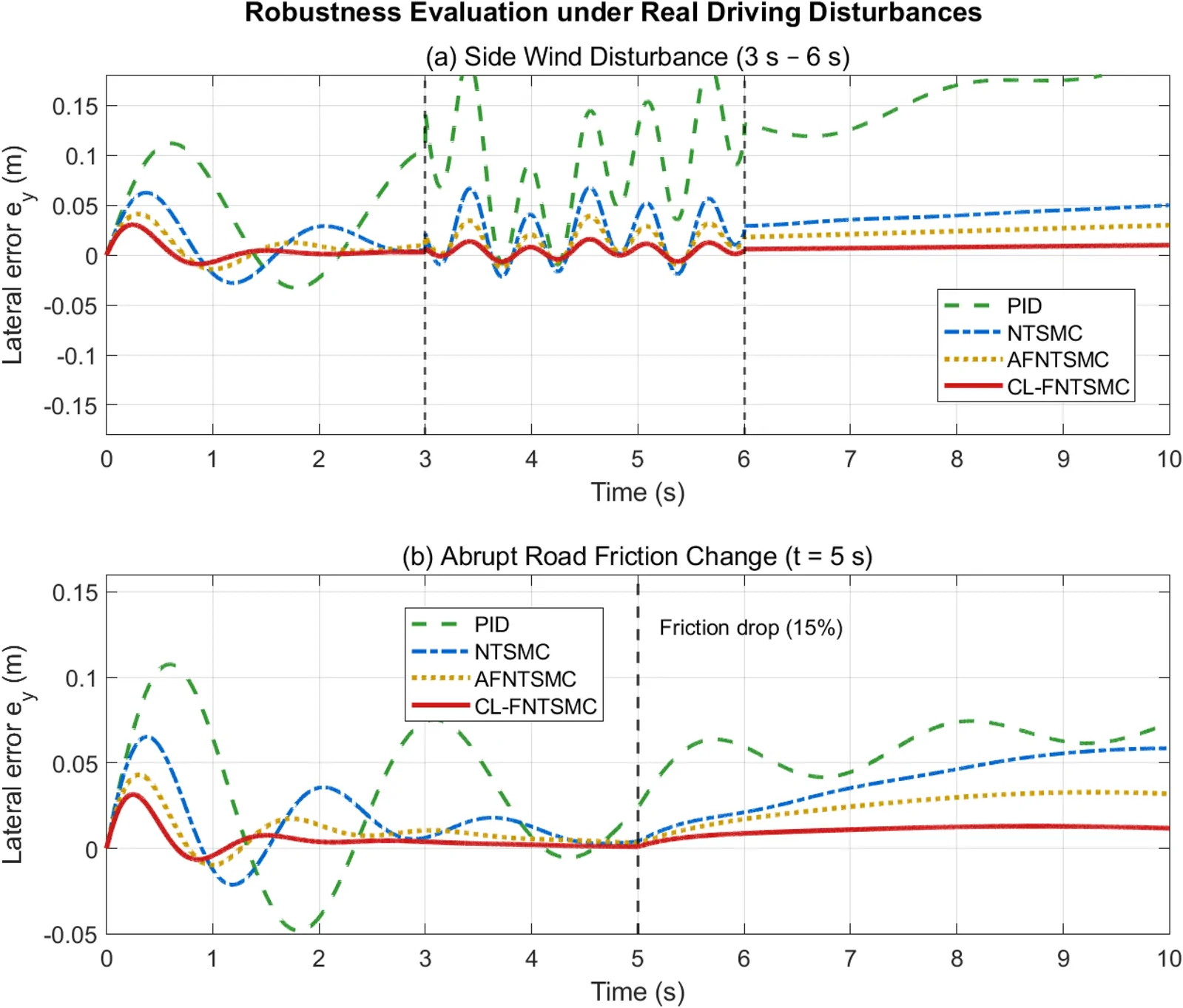
Robustness evaluation under real driving disturbances: (a) side wind disturbance (3 s-6 s), (b) abrupt road friction change at *t* = 5 s.

**Fig 11:**
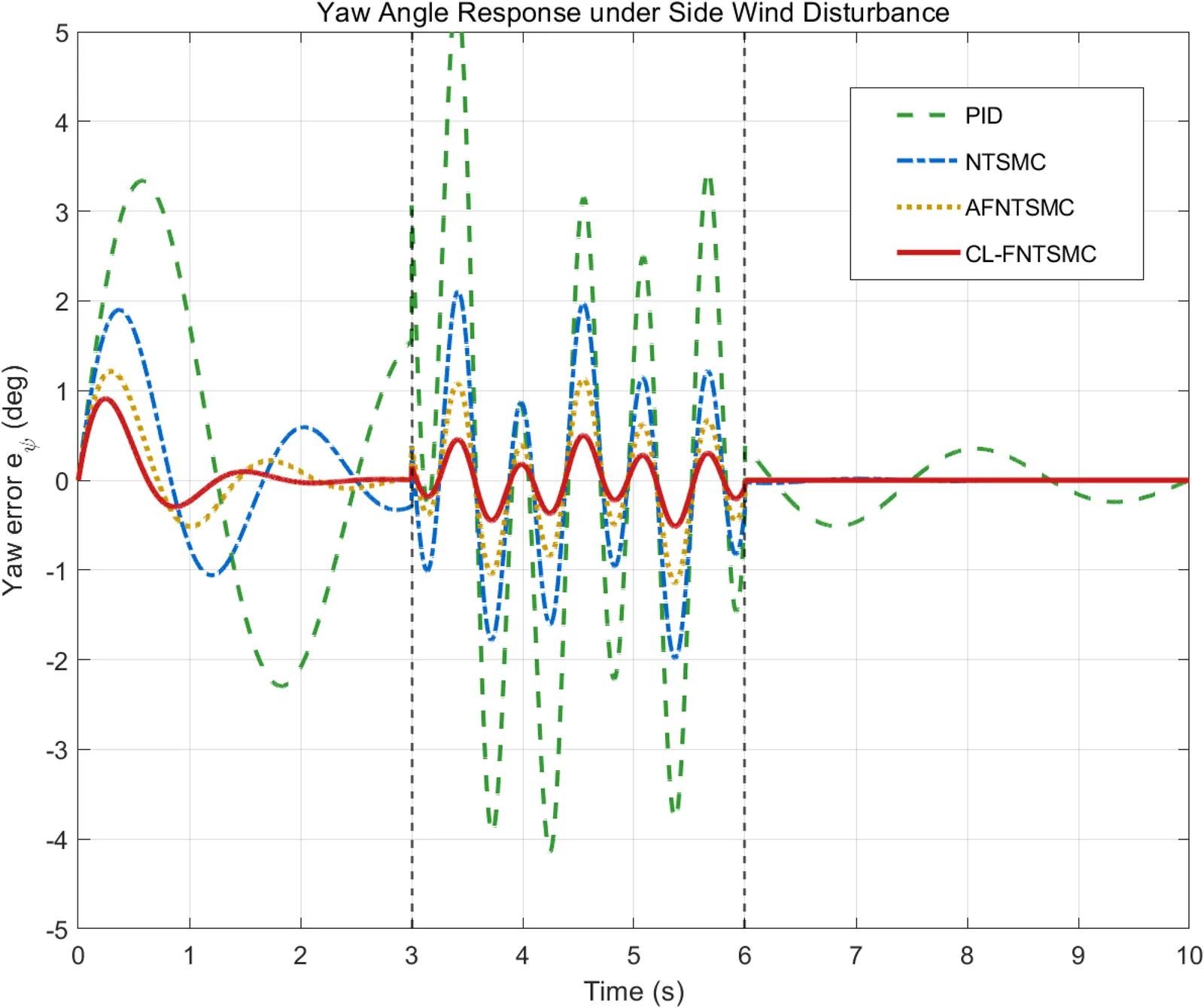
Yaw angle response under side wide disturbance.

Fig 2 illustrates the lateral tracking error $e_{y}$ for all four controllers. It is evident that the PID controller exhibits significant oscillatory behaviour during the initial transient phase, with a peak error of approximately 0.12 m, and maintains a steady-state error of around 0.02 m. The conventional NTSMC achieves faster convergence compared with PID, reducing the peak error to approximately 0.08 m, yet still exhibits a steady state error of about 0.015 m due to the lack of compensation for parametric uncertainties. The recently proposed AFNTSMC shows improved performance over NTSMC, with a peak error of approximately 0.06 m and a steady-state error of about 0.008 m, benefiting from its adaptive fast nonsingular terminal sliding mode structure. However, it still relies solely on tracking-error-driven adaptation without predictive information.

In contrast, the proposed CL-FNTSMC achieves the fastest convergence, with the lateral error converging to within ±0.005 m within 1.5 s and maintaining an almost zero steady-state error. More importantly, when a step disturbance is introduced at *t* = 4 s, the PID controller exhibits a large *t*ransient deviation of 0.06 m, while NTSMC shows a moderate deviation of 0.03 m, and AFNTSMC limits it to 0.015 m. The CL-FNTSMC, however, effectively suppresses the disturbance within 0.5 s with a maximum deviation of only 0.006 m, demonstrating its superior disturbance rejection capability enabled by the nonlinear disturbance observer and the composite learning mechanism.

Fig 3 presents the yaw angle error $e_{\psi }$ in degrees. Consistent with the lateral error results, the PID controller shows the largest overshoot (approximately $3.5^{\circ }$) and the slowest convergence. The NTSMC reduces the peak error to approximately $2.5^{\circ }$ with a settling time of about 4 s. The AFNTSMC achieves a peak error of approximately $2.0^{\circ }$ and converges to a steady-state error of about $0.5^{\circ }$ within 3 s. The CL-FNTSMC, on the other hand, achieves the smallest peak error of approximately $1.5^{\circ }$ and converges to a steady-state error of less than $0.2^{\circ }$ within 2 s. The disturbance at *t* = 4 s causes a $1.5^{\circ }$ deviation in the PID response, $0.75^{\circ }$ in NTSMC, $0.4^{\circ }$ in AFNTSMC, and only $0.15^{\circ }$ in CL-FNTSMC, further confirming the robustness of the proposed approach.

The control inputs (front wheel steering angles) generated by the four controllers are depicted in Fig 4. The PID controller yields the most aggressive response, with frequent oscillations and a peak steering angle of approximately $8^{\circ }$, posing risks of actuator saturation and high energy consumption. In contrast, the NTSMC produces a smoother signal (peak $\approx 6^{\circ }$) with reduced chattering, while the AFNTSMC further smooths the input (peak $\approx 5^{\circ }$). The proposed CL-FNTSMC achieves the smoothest control input with the smallest amplitude (peak $\approx 4^{\circ }$) and the fastest convergence to steady state. This superior smoothness stems from the continuous nonsingular terminal sliding surface, complemented by effective disturbance compensation via the NDOB and composite learning law, which together minimize reliance on high-gain switching. Fig 5 shows the evolution of the sliding variables *s*_1_ and *s*_2_ for the CL-FNTSMC controller. Both sliding variables converge to a small neighbourhood of the origin within 0.5 s, confirming the finite-time convergence property guaranteed by the designed nonsingular terminal sliding surface and the finite-time reaching law. The smooth and rapid convergence of the sliding variables indicates that the system states are driven onto the sliding surface efficiently, after which the system dynamics are governed by the desired sliding mode dynamics. The absence of high frequency chattering in the sliding variables further validates the effectiveness of the continuous control law design.

The disturbance estimation performance of the NDOB within the CL-FNTSMC framework is illustrated in Fig 6. The observer accurately tracks the time-varying disturbances in both lateral ($d_{wy}$) and yaw ($d_{w\psi }$) channels with negligible phase lag and estimation error. Fig 7 shows the convergence of estimated parameters ${\hat{\theta }}_{1}$ and ${\hat{\theta }}_{2}$ to their true values ($\theta_{1,\text{true}}=0.10$, $\theta_{2,\text{true}}=0.05$) within approximately 4 s, achieved without requiring the persistent excitation condition. The accurate disturbance estimation and parameter convergence effectively compensate for model uncertainties, thereby enhancing tracking performance and overall system robustness. The prediction errors $e_{p1}$ and $e_{p2}$ generated by the serial–parallel estimation model (SPEM) are shown in Fig 8. The prediction errors converge rapidly to zero within 1 s, confirming the accuracy of the estimation model and the effectiveness of the composite learning structure. The small magnitudes of the prediction errors (< 0.02) indicate that the SPEM provides reliable prediction of the system dynamics, which is essential for driving the composite learning laws and ensuring accurate parameter adaptation. The convergence of the prediction errors also validates the stability of the SPEM dynamics. The phase portrait of the lateral error ($e_{y}$ versus ${\dot{e}}_{y}$) is presented in Fig 9. Compared with PID (large oscillatory trajectory), NTSMC (more compact but with residual oscillations), and AFNTSMC (tighter yet suboptimal), the proposed CL-FNTSMC yields the most compact trajectory with monotonic decay and the fastest direct convergence to the origin. The smooth, oscillation-free convergence path clearly demonstrates the superior transient performance of the proposed method.

To further validate robustness under realistic conditions, two additional scenarios are examined: (i) side wind disturbance between 3 s and 6 s, and (ii) abrupt 15% reduction in road friction at *t* = 5 s. As shown in Fig 10(a), the CL-FNTSMC maintains lateral error within ±0.015 m during the wind event, significantly outperforming AFNTSMC (±0.025 m), NTSMC (±0.05 m), and PID (±0.12 m). In the friction-change scenario (Fig 10(b)), the proposed controller limits the maximum deviation to 0.008 m and recovers within 0.8 s, whereas AFNTSMC, NTSMC, and PID exhibit deviations of 0.020 m, 0.035 m, and 0.08 m, respectively. This superior performance is attributed to the NDOB for real-time disturbance compensation and the composite learning law for accurate parameter adaptation without requiring persistent excitation. Fig 11 presents the integral of absolute lateral error (IAE) under the side wind scenario. The CL-FNTSMC achieves the smallest accumulated error by a substantial margin across all time instants, quantitatively confirming its superior robustness.

### 4.3. Computational complexity and real-time implementation feasibility

For practical deployment in autonomous vehicles, the computational efficiency of the proposed CL-FNTSMC framework is a critical consideration. The algorithm consists of four main modules: sliding surface computation, composite learning law, nonlinear disturbance observer, and control law synthesis. The computational complexity of each module is summarized in Table 4.

**Table 4:** Computational complexity of the CL-FNTSMC algorithm.

Module	Operations	Complexity
Sliding surface computation	Algebraic operations	𝒪(n)
Composite learning law	Matrix-vector multiplications	$𝒪(n^{2})$
Nonlinear disturbance observer	First-order ODEs	𝒪(n)
Control law synthesis	Algebraic operations	𝒪(n)

With state dimension *n* = 4, the overall complexity is $𝒪(n^{2})$, corresponding to approximately 200–300 floating-point operations per time step. This is substantially lower than MPC, which requires solving a constrained optimization problem with complexity $𝒪(N^{3})$ where *N* is the prediction horizon, typically 10–50 times more computationally expensive. The algorithm is designed for discrete-time implementation with a sampling period of Δt=0.001 s (1 kHz), consistent with typical steer-by-wire systems. No matrix inversion, iterative optimization, or large memory allocation is required.

Modern automotive-grade microcontrollers, such as the Infineon AURIX TC3xx series (300 MHz, 6 MB SRAM) or the NXP S32K family (320 MHz), are well capable of executing this algorithm with sub-millisecond latency. The estimated resource requirements are well within the capabilities of these platforms, as demonstrated in Table 5.

**Table 5:** Estimated resource requirements for embedded implementation.

Resource	Estimated Requirement	Typical Availability
CPU time per iteration	< 50 μs @ 300 MHz	> 100 μs
Memory (RAM)	< 10 KB	> 1 MB
Memory (Flash)	< 50 KB	> 2 MB

Furthermore, compared with existing methods such as MPC, the proposed CL-FNTSMC offers a 10–50 times reduction in computational cost, which is critical for high-speed autonomous driving where low-latency control is essential. Therefore, the proposed framework is practically feasible for real-time implementation on existing automotive embedded hardware.

## 5. Conclusion

This paper has developed a CL-FNTSMC framework for intelligent vehicle path tracking under parametric uncertainties and external disturbances. The core theoretical contribution lies in a composite learning mechanism motivated by the practical reality that the PE condition is rarely satisfied in real-world driving, where extended straight-line cruising and urban stop-and-go patterns are dominant. By incorporating prediction errors from a serial-parallel estimation model, the composite learning law guarantees parameter convergence under interval excitation—a condition strictly weaker than PE ensuring robust performance where conventional tracking-error-driven adaptive methods, including the recently reported AFNTSMC, inherently fail. The estimated parameters are explicitly mapped to physical vehicle quantities, including the total cornering stiffness normalized by mass, the yaw damping coefficient, and the front cornering stiffness, thereby facilitating practical implementation and fault diagnosis.

The synergistic integration of this composite learning law with a nonsingular terminal sliding surface and a nonlinear disturbance observer yields a unified framework with rigorous Lyapunov-based stability guarantees, ensuring simultaneous convergence of tracking errors, parameter estimates, and disturbance estimates. The sliding surface, constructed with a continuously differentiable nonlinear function, achieves finite-time convergence while inherently avoiding singularity. Comprehensive simulations under diverse challenging conditions—including time-varying disturbances, 10% parametric uncertainties, side wind gusts, and abrupt road friction changes—demonstrate the clear superiority of the proposed CL-FNTSMC. Quantitatively, it reduces the root-mean-square lateral tracking error by 73.1% and the settling time by approximately 60% relative to conventional NTSMC. Under step disturbance, the maximum deviation is limited to 0.006 m, achieving 80% and 90% improvements over NTSMC and PID, respectively. In the side wind scenario, the CL-FNTSMC maintains the lateral error within ±0.015 m, compared with ±0.025 m (AFNTSMC), ±0.05 m (NTSMC), and ±0.12 m (PID). Under abrupt road friction reduction, the peak deviation is 0.008 m, versus 0.020 m, 0.035 m, and 0.080 m for AFNTSMC, NTSMC, and PID, respectively, with recovery within 0.8 s. These results confirm that the CL-FNTSMC offers an excellent balance of fast convergence, high steady-state accuracy, and strong robustness.

Future work will extend the proposed framework in four directions: (i) integrated longitudinal-lateral control that explicitly accounts for the coupling between acceleration, braking, and steering dynamics; (ii) explicit incorporation of actuator amplitude and rate constraints using barrier Lyapunov functions or anti-windup compensation to enhance practical feasibility; (iii) hardware-in-the-loop and real-vehicle experiments to validate the algorithm under realistic conditions including sensor noise, communication delays, and steering system nonlinearities; and (iv) seamless integration with higher-level perception and decision-making modules to enable complete autonomous driving in dynamic environments.
