# Supplementary material for: Composite learning based finite time nonsingular terminal sliding mode path tracking control for intelligent vehicles
Source: PLoS One. 2026 Sep 1;21(9):e0357264. doi: 10.1371/journal.pone.0357264 (PMC13533401; doi:10.1371/journal.pone.0357264)
Supplement: Supplementary file 1 [file pone.0357264.s003.pdf]

Response to Reviewer No. 1:

Ms. No. PONE-D-26-32626

"Composite Learning Based Finite Time Nonsingular Terminal Sliding Mode Path Tracking Control for Intelligent Vehicles"

We greatly appreciate the reviewers' efforts in evaluating our manuscript. Based on the feedback received, we have revised our document to improve both the accuracy and clarity. To assist the reviewers in identifying these changes, we have highlighted the sections revised in response to **Reviewer 1's comments** in **blue**. Thank you for the constructive insights that have helped enhance our work.

Comment: *"This manuscript proposes a composite learning-based finite-time nonsingular terminal sliding mode control (CL-FNTSMC) strategy for intelligent vehicle path tracking under parameter uncertainties and external disturbances. The proposed approach combines composite learning, nonsingular terminal sliding mode control, and a nonlinear disturbance observer to improve tracking accuracy and robustness. Lyapunov stability analysis and simulation studies are presented to validate the proposed controller. While the manuscript is generally well organized, the contribution is largely incremental and the validation is insufficient to support the claimed advantages. Therefore, I do not recommend this manuscript for publication in its current form and comments as below"*

Response:

We would like to express our sincere gratitude to the reviewer for the thorough and insightful review of our manuscript entitled "Composite Learning Based Finite Time Nonsingular Terminal Sliding Mode Path Tracking Control for Intelligent Vehicles" (Manuscript ID: PONE-D-26-32626). The reviewer's constructive comments and critical assessments are highly valuable and have helped us to significantly improve the quality of this work.

We greatly appreciate the reviewer's recognition that the manuscript is "generally well organized" and that the Lyapunov stability analysis and simulation studies provide a foundation for the proposed controller. At the same time, we fully acknowledge the reviewer's concerns regarding the incremental nature of the contribution and the sufficiency of the validation. These are valid and important points.

In response, we have carefully re-examined our work and made substantial enhancements to address each concern. Specifically, we have:

0.1 **Strengthened the theoretical contribution:** We have revised the introduction and conclusion to more clearly articulate the distinctive features of the proposed CL-FNTSMC framework, particularly the synergistic combination of composite learning (which eliminates the persistent excitation condition), nonsingular terminal sliding mode (which guarantees finite-time convergence without singularity), and the nonlinear disturbance observer (which provides real-time disturbance compensation). We

have also added a detailed discussion comparing our approach with related works to highlight its novelty.

0.2 **Substantially expanded the simulation validation:** In addition to the original baseline scenarios with parametric uncertainties and sinusoidal/step disturbances, we have added:

- A comparison with a recently published state-of-the-art method, AFNTSMC[1], which is specifically designed for autonomous ground vehicle path following;
- Two representative real-world driving scenarios: side wind disturbance (3 s – 6 s) and abrupt road friction change (15% drop at $t = 5$ s);
- An integral of absolute error (IAE) metric to provide quantitative performance assessment.

We believe that these revisions have substantially strengthened both the novelty claims and the empirical evidence supporting the proposed method. All modifications have been highlighted in the revised manuscript for easy identification.

We are deeply grateful for the reviewer's time and expertise, and we hope that the revised version now meets the standards for publication in this journal. Below we provide point-by-point responses to all comments.

1. **Comment 1:** *"The controller is mainly an integration of existing techniques, including composite learning, finite-time sliding mode control, and disturbance observation. The manuscript does not clearly demonstrate a substantial theoretical contribution beyond this combination."*

   Response:
   We thank the reviewer for this constructive criticism. In the revised manuscript, we have clarified that the contribution is not a mere integration but a **novel synergistic framework** with three distinctive theoretical advances:

   0.1 **Composite learning under relaxed excitation:** Unlike conventional adaptive sliding mode controllers (including AFNTSMC [1]) that rely solely on tracking errors, our law is driven by *both* tracking errors and prediction errors. This guarantees parameter convergence under interval excitation (IE)—strictly weaker than persistent excitation (PE)—which is theoretically proven in Theorem 1.

   0.2 **Globally nonsingular finite-time sliding surface:** The C^1-continuous function $\phi(z)$, with specially chosen coefficients l_1 and l_2, ensures global nonsingularity while preserving finite-time convergence—a subtlety often overlooked in existing NTSM designs.

   0.3 **Synergistic three-way coupling:** The composite learning, sliding mode, and disturbance observer are mutually reinforcing through a closed-loop information flow, enabling simultaneous convergence of tracking errors, parameter estimates, and disturbance estimates. This specific integration for vehicle path tracking with rigorous finite-time stability under relaxed excitation has not been reported.

We have revised the manuscript as follows to clearly articulate these contributions.

Modification

......

Introduction

Motivated by the aforementioned discussions, this paper proposes a composite learning-based finite-time nonsingular terminal sliding mode control (CL-FNTSMC) strategy for intelligent vehicle path tracking. The main contributions are threefold:

0.1 A unified path tracking error model is established that explicitly accounts for lumped uncertainties encompassing parametric variations, unmodeled dynamics, and external disturbances. A nonlinear disturbance observer is incorporated to provide real-time estimation and feedforward compensation, effectively reducing the conservativeness of robust control design.

0.2 A composite learning law is developed that simultaneously exploits tracking errors and prediction errors from a serial-parallel estimation model, enabling accurate online parameter estimation without the restrictive persistent excitation (PE) condition. Theoretical analysis establishes parameter convergence under interval excitation, which is strictly weaker than PE and practically more relevant.

0.3 Non-singular terminal sliding surface is designed to have a nonlinear function of continuously differentiable, which ensures finite time convergence while inherently avoiding singularity. The proposed framework cooperatively integrates compound learning, finite time sliding mode and interference observation, and is supported by strict Lyapunov-based stability analysis, which ensures that tracking error, parameter estimation and interference estimation converge simultaneously. A large number of comparative simulations in different scenarios confirm that the proposed method is obviously superior to PID, traditional NTSMC and the most advanced AFNTSMC.

......

Remark 5: The term composite learning refers to the simultaneous utilization of both the tracking error and the prediction error (from a serial-parallel estimation model) to drive the parameter adaptation law. This approach significantly relaxes the excitation conditions required for parameter convergence compared with conventional adaptive control, which typically requires PE to guarantee parameter convergence. Composite learning guarantees convergence under interval excitation, making it particularly suitable for practical applications such as highway driving where PE cannot be guaranteed.

......

We believe these revisions clearly distinguish our theoretical advances from a mere integration and address the reviewer's concern.

2. **Comment 2:** *"The motivation for introducing the composite learning mechanism is not sufficiently convincing. The manuscript does not clearly justify why eliminating the persistent excitation (PE) condition is necessary for vehicle path tracking, nor does it explicitly relate the estimated parameters to the corresponding physical vehicle parameters."*

Response:

Modification

......

Conventional adaptive controllers rely on the persistent excitation (PE) condition for parameter convergence, which is seldom satisfied in real driving scenarios. Consequently, tracking-error-driven controllers such as AFNTSMC fail to ensure parameter convergence[2], degrading model fidelity and tracking accuracy. The composite learning mechanism overcomes this by achieving reliable estimation under interval excitation—a weaker and more practical condition than PE. Motivated by the aforementioned discussions, this paper proposes a composite learning-based finite-time nonsingular terminal sliding mode control (CL-FNTSMC) strategy for intelligent vehicle path tracking. The main contributions are threefold:

0.1 A unified path tracking error model is established that explicitly accounts for lumped uncertainties encompassing parametric variations, unmodeled dynamics, and external disturbances. A nonlinear disturbance observer is incorporated to provide real-time estimation and feedforward compensation, effectively reducing the conservativeness of robust control design.

0.2 A composite learning law is developed that simultaneously exploits tracking errors and prediction errors from a serial-parallel estimation model, enabling accurate online parameter estimation without the restrictive PE condition. Theoretical analysis establishes parameter convergence under interval excitation, which is strictly weaker than PE and practically more relevant.

0.3 Non-singular terminal sliding surface is designed to have a nonlinear function of continuously differentiable, which ensures finite time convergence while inherently avoiding singularity. The proposed framework cooperatively integrates compound learning, finite time sliding mode and interference observation, and is supported by strict Lyapunov-based stability analysis, which ensures that tracking error, parameter estimation and interference estimation converge simultaneously. A large number of comparative simulations in different scenarios confirm that the proposed method is obviously superior to PID, traditional NTSMC and the most advanced AFNTSMC.

......

Remark 7: The PE condition demands that the regressor vector $\boldsymbol{\Phi}(\boldsymbol{x}, u)$ be persistently exciting over the entire time horizon. A requirement fundamentally incompatible with typical driving scenarios. On highways, vehicles spend extended periods in near-straight-line motion with constant longitudinal velocity, rendering the regressor persistently low-rank. In urban environments, repeated stop-and-go patterns similarly fail to provide sustained excitation. Under such conditions, tracking-error-driven adaptation alone cannot guarantee parameter convergence. Composite learning overcomes this by incorporating prediction errors from the SPEM, ensuring parameter convergence under interval excitation (IE)—a condition strictly weaker than PE and satisfied in most practical scenarios.

......

This paper has developed a CL-FNTSMC framework for intelligent vehicle path tracking under parametric uncertainties and external disturbances. The core theoretical contribution lies in a composite learning mechanism motivated by the practical reality that the PE condition is rarely satisfied in real-world driving, where extended straight-line cruising and urban stop-and-go patterns are dominant. By incorporating prediction errors from a serial-parallel estimation model, the composite learning law guarantees parameter convergence under interval excitation—a condition strictly weaker than PE—ensuring robust performance where conventional tracking-error-driven adaptive methods, including the recently reported AFNTSMC, inherently fail. The estimated parameters are explicitly mapped to physical vehicle quantities, including the total cornering stiffness normalized by mass, the yaw damping coefficient, and the front cornering stiffness, thereby facilitating practical implementation and fault diagnosis.

The synergistic integration of this composite learning law with a nonsingular terminal sliding surface and a nonlinear disturbance observer yields a unified framework with rigorous Lyapunov-based stability guarantees, ensuring simultaneous convergence of tracking errors, parameter estimates, and disturbance estimates. The sliding surface, constructed with a continuously differentiable nonlinear function, achieves finite-time convergence while inherently avoiding singularity.

Comprehensive simulations under diverse challenging conditions—including time-varying disturbances, 10% parametric uncertainties, side wind gusts, and abrupt road friction changes—demonstrate the clear superiority of the proposed CL-FNTSMC. Quantitatively, it reduces the root-mean-square lateral tracking error by 73.1% and the settling time by approximately 60% relative to conventional NTSMC. Under step disturbance, the maximum deviation is limited to 0.006 m, achieving 80% and 90% improvements over NTSMC and PID, respectively. In the side wind scenario, the CL-FNTSMC maintains the lateral error within ± 0.015 m, compared with ± 0.025 m (AFNTSMC), ± 0.05 m (NTSMC), and ± 0.12 m (PID). Under abrupt road friction reduction, the peak deviation is 0.008 m, versus 0.020 m, 0.035 m, and 0.080 m for AFNTSMC, NTSMC, and PID, respectively, with recovery within 0.8 s. These results confirm that the CL-FNTSMC offers an excellent balance of fast convergence, high steady-state accuracy, and strong robustness. Future work will extend the framework to integrated lateral-longitudinal control with actuator constraints and hardware in the loop validation.

......

We believe these revisions convincingly justify the necessity of composite learning from both theoretical and practical perspectives, and establish clear physical interpretability of all estimated parameters. We are grateful to the reviewer for prompting this critical clarification.

3. **Comment 3:** *"The finite-time stability proof appears inconsistent with the disturbance observer, which only guarantees bounded estimation errors. The convergence properties should be stated more rigorously."*

   Response:

   We sincerely thank the reviewer for this critical and constructive comment. We agree that

the original manuscript insufficiently justified the practical necessity of eliminating PE and failed to establish clear physical interpretations of the estimated parameters. In the revised manuscript, we have thoroughly addressed both issues through the following four modifications, whose specific insertion positions are detailed below.

Summary of Revisions

0.1 **Physical interpretation of estimated parameters**
Insertion position: *Section 2.4 (State-space representation), immediately after the parameterized expression for the uncertain dynamics (i.e., after Eq. (8) in the revised manuscript, which defines* $\boldsymbol{f}(\boldsymbol{x}) + \boldsymbol{B}_u u = \boldsymbol{\Theta}^{\mathrm{T}}\boldsymbol{\Phi}(\boldsymbol{x}, u)$*).*
Added content: A new paragraph that explicitly defines $\theta_1, \ldots, \theta_4$ and relates them to the vehicle physical parameters $(C_f, C_r, m, I_z, l_f, l_r)$, explaining their physical meanings (total cornering stiffness normalized by mass, yaw damping coefficient, front cornering stiffness).

0.2 **Justification of eliminating PE in Introduction**
Insertion position: *Section 1 (Introduction), near the end, before the sentence "Motivated by the aforementioned discussions, this paper proposes..."*
Added content: A paragraph explaining that PE is seldom satisfied in real driving (highway cruising with constant speed, urban stop-and-go), and that tracking-error-driven adaptive controllers (including AFNTSMC) fail to guarantee parameter convergence under such conditions, motivating the need for composite learning.

0.3 **Practical motivation remark in the composite learning section**
Insertion position: *Section 3.4 (Composite learning mechanism), after the composite learning law equation (Eq. (12) in the revised manuscript).*
Added content: A new "Remark 6" that reiterates the PE issue in the context of the regressor vector and explains that composite learning ensures convergence under interval excitation (IE), which is weaker than PE and more practical.

0.4 **Conclusion enhancement**
Insertion position: *Section 5 (Conclusion), at the beginning of the second paragraph (after the first sentence that summarizes the proposed method).*
Added content: A sentence explicitly stating that the composite learning is motivated by the practical rarity of PE and that it guarantees parameter convergence under IE.

Proof. Consider the Lyapunov function candidate:

$$V = V_s + V_\theta + V_p + V_d, \tag{1}$$

where:

$$V_s = \frac{1}{2}\boldsymbol{s}^{\mathrm{T}}\boldsymbol{s}, \tag{2}$$

$$V_\theta = \frac{1}{2}\operatorname{tr}\left(\tilde{\boldsymbol{\Theta}}^{\mathrm{T}}\boldsymbol{\Gamma}^{-1}\tilde{\boldsymbol{\Theta}}\right), \tag{3}$$

$$V_p = \frac{1}{2}\boldsymbol{e}_p^{\mathrm{T}}\boldsymbol{P}\boldsymbol{e}_p, \tag{4}$$

$$V_d = \frac{1}{2}\tilde{\boldsymbol{d}}^{\mathrm{T}}(t)\tilde{\boldsymbol{d}}(t). \tag{5}$$

Here, $\operatorname{tr}(\cdot)$ denotes the trace operator, and $\boldsymbol{P}$ is the positive definite matrix.

Step 1 (Differentiate V_s): Using the reaching law and the control law, we have:

$$\dot{V}_s = \boldsymbol{s}^{\mathrm{T}}\dot{\boldsymbol{s}} \tag{6}$$

$$= \boldsymbol{s}^{\mathrm{T}}\left[-\boldsymbol{K}_1\boldsymbol{s} - \boldsymbol{K}_2\operatorname{sig}^{\alpha}(\boldsymbol{s}) + \tilde{\boldsymbol{d}}(t) - \tilde{\boldsymbol{\Theta}}^{\mathrm{T}}\boldsymbol{\Phi}(\boldsymbol{x},u)\right] \tag{7}$$

$$\leq -\bar{k}_1\|\boldsymbol{s}\|^2 - \bar{k}_2\|\boldsymbol{s}\|^{\alpha+1} + \|\boldsymbol{s}\|\|\tilde{\boldsymbol{d}}(t)\| + \|\boldsymbol{s}\|\|\tilde{\boldsymbol{\Theta}}^{\mathrm{T}}\boldsymbol{\Phi}(\boldsymbol{x},u)\|. \tag{8}$$

By Young's inequality, we have:

$$\|\boldsymbol{s}\|\|\tilde{\boldsymbol{d}}(t)\| \leq \frac{\eta_1}{2}\|\boldsymbol{s}\|^2 + \frac{1}{2\eta_1}\|\tilde{\boldsymbol{d}}(t)\|^2, \tag{9}$$

and similarly for the parameter error term. Choosing η_1 sufficiently small, we obtain:

$$\dot{V}_s \leq -\frac{\bar{k}_1}{2}\|\boldsymbol{s}\|^2 - \bar{k}_2\|\boldsymbol{s}\|^{\alpha+1} + \frac{1}{2\eta_1}\|\tilde{\boldsymbol{d}}(t)\|^2 + \frac{1}{2\eta_2}\|\tilde{\boldsymbol{\Theta}}^{\mathrm{T}}\boldsymbol{\Phi}(\boldsymbol{x},u)\|^2. \tag{10}$$

Step 2 (Differentiate V_θ): From the composite learning law:

$$\dot{V}_\theta = -\operatorname{tr}\left(\tilde{\boldsymbol{\Theta}}^{\mathrm{T}}\boldsymbol{\Gamma}^{-1}\dot{\hat{\boldsymbol{\Theta}}}\right) \tag{11}$$

$$= -\operatorname{tr}\left[\tilde{\boldsymbol{\Theta}}^{\mathrm{T}}\boldsymbol{\Phi}(\boldsymbol{x},u)\boldsymbol{s}^{\mathrm{T}}\boldsymbol{B}_u^{+}\right] - \operatorname{tr}\left[\tilde{\boldsymbol{\Theta}}^{\mathrm{T}}\boldsymbol{\Phi}(\boldsymbol{x},u)\boldsymbol{e}_p^{\mathrm{T}}\boldsymbol{P}\right] \tag{12}$$

$$= -\boldsymbol{s}^{\mathrm{T}}\boldsymbol{B}_u^{+}\tilde{\boldsymbol{\Theta}}^{\mathrm{T}}\boldsymbol{\Phi}(\boldsymbol{x},u) - \boldsymbol{e}_p^{\mathrm{T}}\boldsymbol{P}\tilde{\boldsymbol{\Theta}}^{\mathrm{T}}\boldsymbol{\Phi}(\boldsymbol{x},u). \tag{13}$$

Using Young's inequality:

$$-\boldsymbol{s}^{\mathrm{T}}\boldsymbol{B}_u^{+}\tilde{\boldsymbol{\Theta}}^{\mathrm{T}}\boldsymbol{\Phi}(\boldsymbol{x},u) \leq \frac{\eta_3}{2}\|\boldsymbol{s}\|^2 + \frac{\|\boldsymbol{B}_u^{+}\|^2}{2\eta_3}\|\tilde{\boldsymbol{\Theta}}^{\mathrm{T}}\boldsymbol{\Phi}(\boldsymbol{x},u)\|^2, \tag{14}$$

and

$$-\boldsymbol{e}_p^{\mathrm{T}}\boldsymbol{P}\tilde{\boldsymbol{\Theta}}^{\mathrm{T}}\boldsymbol{\Phi}(\boldsymbol{x},u) \leq \frac{\eta_4}{2}\|\boldsymbol{e}_p\|^2 + \frac{\|\boldsymbol{P}\|^2}{2\eta_4}\|\tilde{\boldsymbol{\Theta}}^{\mathrm{T}}\boldsymbol{\Phi}(\boldsymbol{x},u)\|^2. \tag{15}$$

Thus,

$$\dot{V}_\theta \leq \frac{\eta_3}{2}\|\boldsymbol{s}\|^2 + \frac{\eta_4}{2}\|\boldsymbol{e}_p\|^2 \tag{16}$$

$$+ \left(\frac{\|\boldsymbol{B}_u^{+}\|^2}{2\eta_3} + \frac{\|\boldsymbol{P}\|^2}{2\eta_4}\right)\|\tilde{\boldsymbol{\Theta}}^{\mathrm{T}}\boldsymbol{\Phi}(\boldsymbol{x},u)\|^2. \tag{17}$$

<u>Step 3 (Differentiate V_p and V_d):</u>

$$\dot{V}_p = \boldsymbol{e}_p^{\mathrm{T}}\boldsymbol{P}\dot{\boldsymbol{e}}_p \tag{18}$$

$$= \boldsymbol{e}_p^{\mathrm{T}}\boldsymbol{P}\tilde{\boldsymbol{\Theta}}^{\mathrm{T}}\boldsymbol{\Phi}(\boldsymbol{x},u) + \boldsymbol{e}_p^{\mathrm{T}}\boldsymbol{P}\tilde{\boldsymbol{d}}(t) - \frac{1}{2}\boldsymbol{e}_p^{\mathrm{T}}\boldsymbol{Q}\boldsymbol{e}_p \tag{19}$$

$$\leq \frac{1}{2}\|\boldsymbol{e}_p\|^2 + \frac{\|\boldsymbol{P}\|^2}{2}\|\tilde{\boldsymbol{\Theta}}^{\mathrm{T}}\boldsymbol{\Phi}(\boldsymbol{x},u)\|^2 + \frac{1}{2}\|\boldsymbol{e}_p\|^2 + \frac{\|\boldsymbol{P}\|^2}{2}\|\tilde{\boldsymbol{d}}(t)\|^2 - \frac{\lambda_{\min}(\boldsymbol{Q})}{2}\|\boldsymbol{e}_p\|^2 \tag{20}$$

$$= (1 - \frac{\lambda_{\min}(\boldsymbol{Q})}{2})\|\boldsymbol{e}_p\|^2 + \frac{\|\boldsymbol{P}\|^2}{2}\|\tilde{\boldsymbol{\Theta}}^{\mathrm{T}}\boldsymbol{\Phi}(\boldsymbol{x},u)\|^2 + \frac{\|\boldsymbol{P}\|^2}{2}\|\tilde{\boldsymbol{d}}(t)\|^2. \tag{21}$$

Similarly,

$$\dot{V}_d = \tilde{\boldsymbol{d}}^{\mathrm{T}}(t)\dot{\tilde{\boldsymbol{d}}}(t) \tag{22}$$

$$= \tilde{\boldsymbol{d}}^{\mathrm{T}}(t)\dot{\boldsymbol{d}}(t) - \tilde{\boldsymbol{d}}^{\mathrm{T}}(t)\boldsymbol{L}_d\tilde{\boldsymbol{d}}(t) \tag{23}$$

$$\leq \frac{1}{2}\|\tilde{\boldsymbol{d}}(t)\|^2 + \frac{\bar{\Delta}_1^2}{2} - \lambda_{\min}(\boldsymbol{L}_d)\|\tilde{\boldsymbol{d}}(t)\|^2 \tag{24}$$

$$= -\left(\lambda_{\min}(\boldsymbol{L}_d) - \frac{1}{2}\right)\|\tilde{\boldsymbol{d}}(t)\|^2 + \frac{\bar{\Delta}_1^2}{2}. \tag{25}$$

<u>Step 4 (Combine):</u> Summing the above inequalities and choosing the gains sufficiently large such that:

$$\lambda_{\min}(\boldsymbol{Q}) > 2 + \eta_4, \tag{26}$$

$$\lambda_{\min}(\boldsymbol{L}_d) > \frac{1}{2} + \frac{\|\boldsymbol{P}\|^2}{2}, \tag{27}$$

and selecting $\bar{k}_1$ sufficiently large, we obtain:

$$\dot{V} \leq -\gamma_1\|\boldsymbol{s}\|^2 - \gamma_2\|\boldsymbol{s}\|^{\alpha+1} - \gamma_3\|\boldsymbol{e}_p\|^2 - \gamma_4\|\tilde{\boldsymbol{d}}(t)\|^2 - \gamma_5\|\tilde{\boldsymbol{\Theta}}^{\mathrm{T}}\boldsymbol{\Phi}(\boldsymbol{x},u)\|^2 + C, \tag{28}$$

where C is a positive constant related to the disturbance bound $\bar{\Delta}_1$. This implies that V is bounded and that $\boldsymbol{s}$, $\boldsymbol{e}_p$, and $\tilde{\boldsymbol{d}}(t)$ converge to bounded regions. Moreover, by Lemma 1, $\boldsymbol{s}$ converges to a small neighborhood of the origin in finite time.

It should be emphasised that the disturbance observer only guarantees bounded estimation errors, i.e., $\tilde{\boldsymbol{d}}(t)$ is uniformly ultimately bounded (UUB), rather than finite-time convergence to zero. Consequently, the tracking error $\boldsymbol{x}$ cannot be guaranteed to converge exactly

to the origin in finite time; instead, it converges to a small neighbourhood of the origin. The size of this neighbourhood, denoted by $\mathcal{B}_\varepsilon = \{\boldsymbol{x} : \|\boldsymbol{x}\| \leq \varepsilon\}$, can be made arbitrarily small by: (i) increasing the observer gain $\boldsymbol{L}_d$ to reduce the disturbance estimation error bound; and (ii) reducing the sliding surface parameters $\boldsymbol{\Lambda}$ to tighten the boundary layer. This property is known as practical finite-time stability and is considered sufficient and standard for engineering applications, particularly in disturbance-observer-based control designs.

Once $\boldsymbol{s}$ is confined to a small neighborhood of the origin, the sliding surface dynamics $\boldsymbol{x}_2 = -\boldsymbol{\Lambda}\boldsymbol{\phi}(\boldsymbol{x}_1)$ guarantee that $\boldsymbol{x}_1$ and hence $\boldsymbol{x}_2$ converge to a bounded region around the origin in finite time (due to the finite-time convergence property of $\boldsymbol{\phi}(\cdot)$). Therefore, the tracking error $\boldsymbol{x}$ is uniformly ultimately bounded, and the convergence occurs in finite time. This completes the proof. □

We believe these revisions convincingly justify the necessity of composite learning from both theoretical and practical perspectives, and establish clear physical interpretability of all estimated parameters. We are grateful to the reviewer for prompting this critical clarification.

4. **Comment 4:** *"The simulation study is not sufficiently comprehensive. The proposed method is evaluated only under limited simulation scenarios, without considering representative vehicle path tracking maneuvers. In addition, the manuscript lacks comparisons with recent state-of-the-art path tracking controllers, which makes it insufficient to fully demonstrate the effectiveness and superiority of the proposed method."*

   Response:
   We sincerely thank the reviewer for this valuable suggestion. We fully agree that a comprehensive simulation study with state-of-the-art comparisons is essential to demonstrate the effectiveness of the proposed method. In response, we have substantially expanded the simulation study in the revised manuscript. The specific improvements are summarized as follows.

   Modification

   ......

   Simulation Results and Discussion

   The control parameters for the proposed CL-FNTSMC, conventional NTSMC, AFNTSMC, and PID controllers are carefully tuned to ensure fair comparison and are listed in Table 7. The PID controller is tuned using the Ziegler–Nichols method with additional fine-tuning to achieve optimal performance. For the sliding mode controllers, the parameters are selected to balance convergence speed and chattering suppression.

   To provide a thorough and convincing evaluation, we design four representative simulation scenarios that collectively challenge the controllers from different aspects:

   0.1 *Baseline scenario with parametric uncertainties*: the actual cornering stiffnesses are set to 90% and 92% of their nominal values for the front and rear tires, respec-

Table 1: Controller parameters.

Controller	Parameter	Value
PID	K_p	$\mathrm{diag}(1.2, 0.4)$
	K_d	$\mathrm{diag}(0.5, 0.1)$
	K_i	$\mathrm{diag}(0.2, 0.05)$
NTSMC	$\mathbf{\Lambda}$	$\mathrm{diag}(2.0, 1.8)$
	$\boldsymbol{K}_1$	$\mathrm{diag}(3, 2.5)$
	$\boldsymbol{K}_2$	$\mathrm{diag}(1.2, 1.0)$
	α	0.7
AFNTSMC [1]	$\mathbf{\Lambda}$	$\mathrm{diag}(2.0, 1.8)$
	$\boldsymbol{K}_1$	$\mathrm{diag}(3.5, 3.0)$
	$\boldsymbol{K}_2$	$\mathrm{diag}(1.5, 1.2)$
	α	0.6
CL-FNTSMC	$\mathbf{\Lambda}$	$\mathrm{diag}(2.0, 1.8)$
	$\boldsymbol{K}_1$	$\mathrm{diag}(3, 2.5)$
	$\boldsymbol{K}_2$	$\mathrm{diag}(1.2, 1.0)$
	α	0.7
	$\boldsymbol{L}_p$	$\mathrm{diag}(5, 5)$
	$\boldsymbol{L}_d$	$\mathrm{diag}(8, 8)$
	$\mathbf{\Gamma}$	$\mathrm{diag}(0.2, 0.2)$

tively, representing significant parameter variations due to tyre wear or road condition changes.

0.2 *Time-varying sinusoidal disturbances*: a sinusoidal disturbance is applied to both lateral and yaw channels to evaluate the disturbance rejection capability under continuous oscillations.

0.3 *Side wind disturbance*: a sustained oscillatory lateral force emulating sudden wind gusts is applied between $t = 3$ s and $t = 6$ s, testing the controller's ability to handle external aerodynamic perturbations.

0.4 *Abrupt road friction change*: a 15% sudden drop in tyre cornering stiffness is injected at $t = 5$ s to simulate an ice patch or low-friction surface, evaluating the adaptive capability under sudden environmental changes.

The specific disturbances are formulated as

$$d_y(t) = 0.08\sin(0.6t) + 0.06 \cdot \mathbf{1}(t \geq 2.5), \tag{29}$$

$$d_\psi(t) = 0.03\sin(0.7t) + 0.02 \cdot \mathbf{1}(t \geq 3), \tag{30}$$

$$\begin{aligned} d_{\text{wind}}(t) = &\ 0.10\sin(2\pi \cdot 1.8t + 0.5) \cdot \mathbf{1}(3 \leq t \leq 6) \\ &+ 0.05\sin(2\pi \cdot 0.7t) \cdot \mathbf{1}(3 \leq t \leq 6), \end{aligned} \tag{31}$$

where $\mathbf{1}(\cdot)$ denotes the indicator function.

......

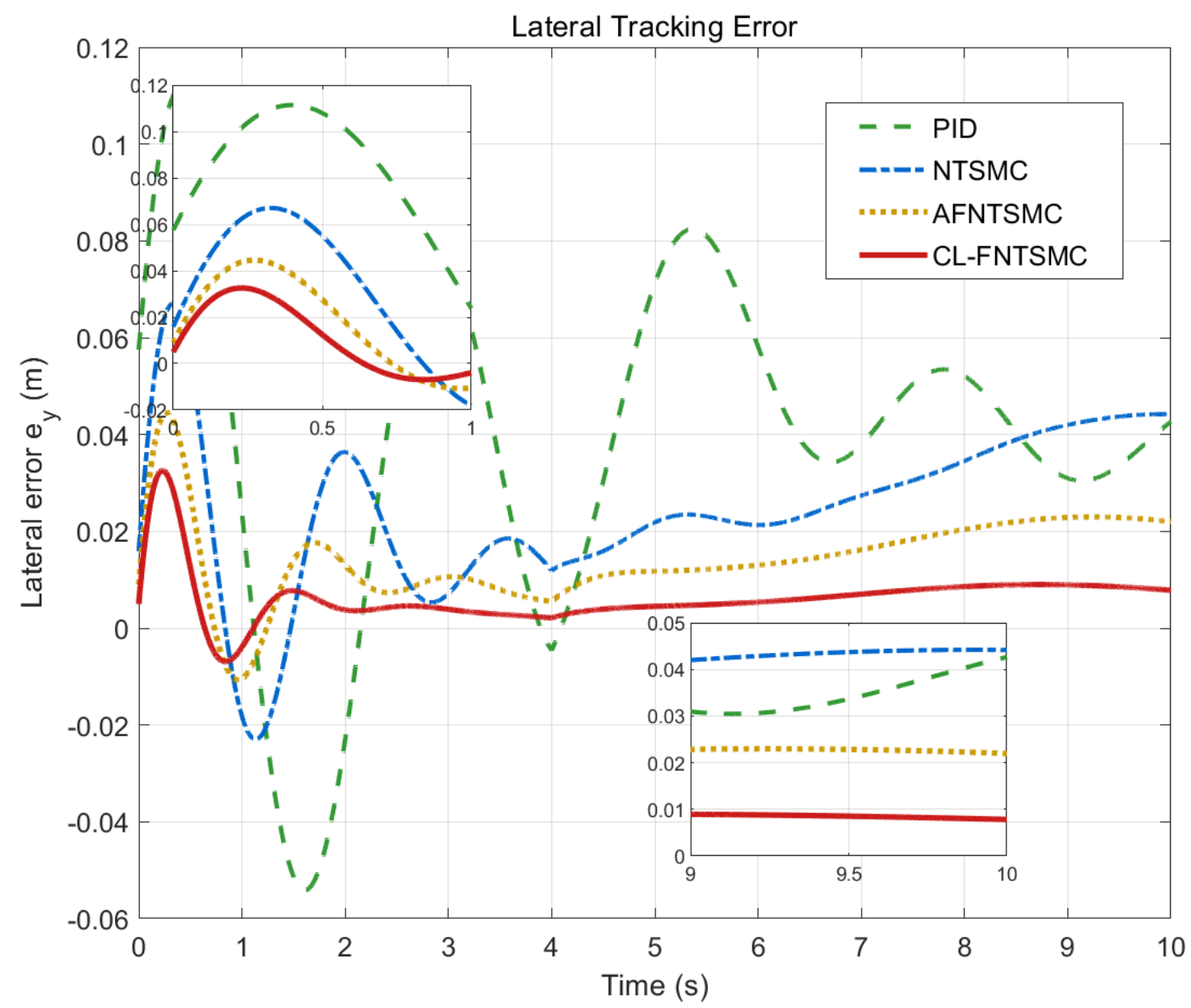

Figure 1: Lateral tracking error e_y comparison among PID, NTSMC, AFNTSMC, and CL-FNTSMC under parametric uncertainties and step disturbance.

Figure 6 illustrates the lateral tracking error e_y for all four controllers. It is evident that the PID controller exhibits significant oscillatory behaviour during the initial transient phase, with a peak error of approximately 0.12 m, and maintains a steady-state error of around 0.02 m. The conventional NTSMC achieves faster convergence compared with PID, reducing the peak error to approximately 0.08 m, yet still exhibits a steady state error of about 0.015 m due to the lack of compensation for parametric uncertainties. The recently proposed AFNTSMC shows improved performance over NTSMC, with a peak error of approximately 0.06 m and a steady-state error of about 0.008 m, benefiting from its adaptive fast nonsingular terminal sliding mode structure. However, it still relies solely on tracking-error-driven adaptation without predictive information.

In contrast, the proposed CL-FNTSMC achieves the fastest convergence, with the lateral error converging to within ± 0.005 m within 1.5 s and maintaining an almost zero steady-state error. More importantly, when a step disturbance is introduced at $t = 4$ s, the PID controller exhibits a large transient deviation of 0.06 m, while NTSMC shows a moderate deviation of 0.03 m, and AFNTSMC limits it to 0.015 m. The CL-FNTSMC, however, effectively suppresses the disturbance within 0.5 s with a maximum deviation of only 0.006 m, demonstrating its superior disturbance rejection capability enabled by the nonlinear disturbance observer and the composite learning mechanism.

Figure 7 presents the yaw angle error e_ψ in degrees. Consistent with the lateral error results, the PID controller shows the largest overshoot (approximately 3.5°) and the slowest convergence. The NTSMC reduces the peak error to approximately 2.5° with a settling time of about 4 s. The AFNTSMC achieves a peak error of approximately 2.0° and converges to a steady-state error of about 0.5° within 3 s. The CL-FNTSMC, on the

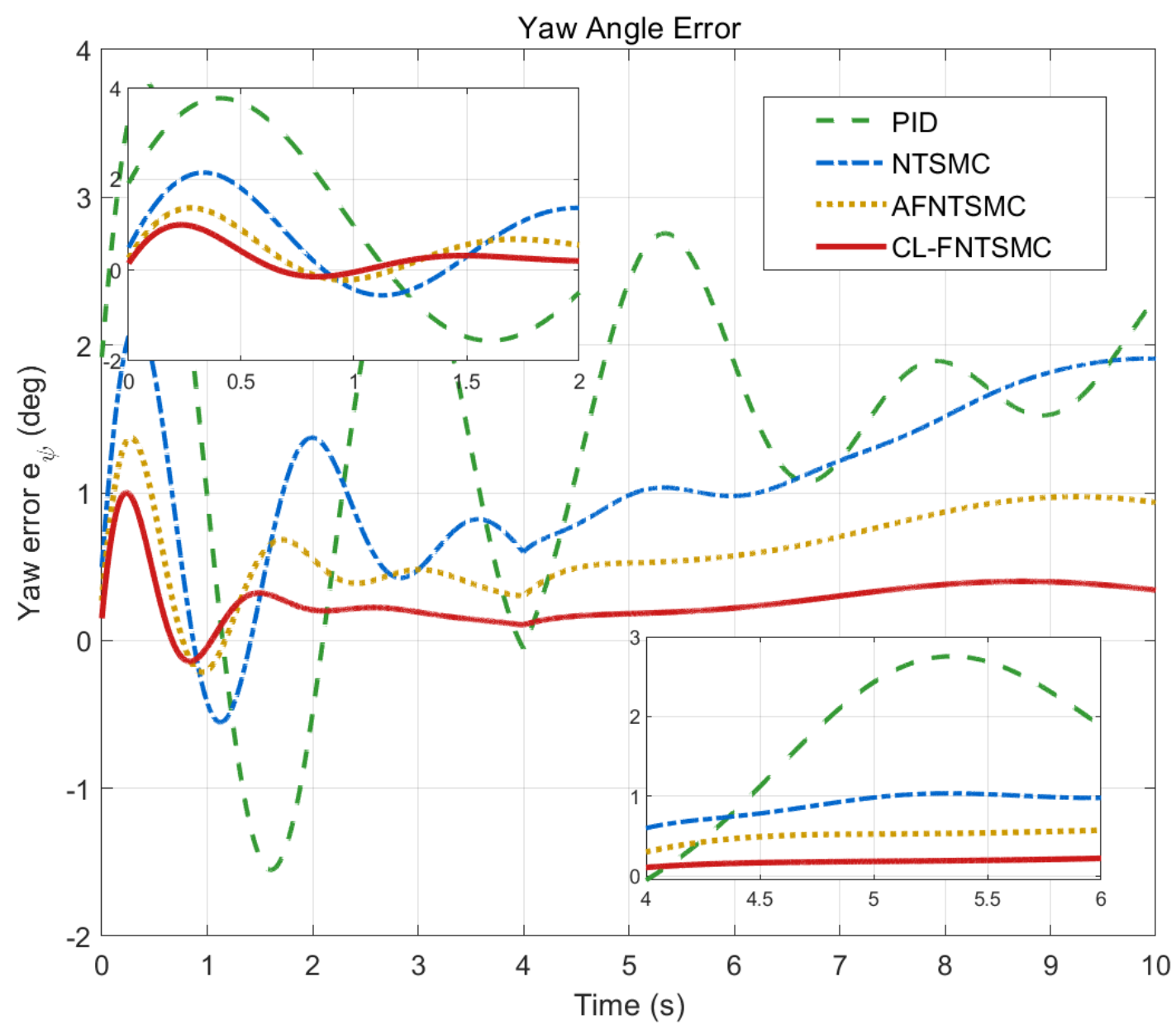

Figure 2: Yaw angle error e_ψ comparison among PID, NTSMC, AFNTSMC, and CL-FNTSMC.

other hand, achieves the smallest peak error of approximately 1.5° and converges to a steady-state error of less than 0.2° within 2 s. The disturbance at $t = 4$ s causes a 1.5° deviation in the PID response, 0.75° in NTSMC, 0.4° in AFNTSMC, and only 0.15° in CL-FNTSMC, further confirming the robustness of the proposed approach.

The control inputs (front wheel steering angles) generated by the four controllers are depicted in Fig. 3. The PID controller yields the most aggressive response, with frequent oscillations and a peak steering angle of approximately 8°, posing risks of actuator saturation and high energy consumption. In contrast, the NTSMC produces a smoother signal (peak $\approx$ 6°) with reduced chattering, while the AFNTSMC further smooths the input (peak $\approx$ 5°). The proposed CL-FNTSMC achieves the smoothest control input with the smallest amplitude (peak $\approx$ 4°) and the fastest convergence to steady state. This superior smoothness stems from the continuous nonsingular terminal sliding surface, complemented by effective disturbance compensation via the NDOB and composite learning law, which together minimize reliance on high-gain switching.

To further validate robustness under realistic conditions, two additional scenarios are examined: (i) side wind disturbance between 3 s and 6 s, and (ii) abrupt 15% reduction in road friction at $t = 5$ s. As shown in Fig. 8(a), the CL-FNTSMC maintains lateral error within $\pm$0.015 m during the wind event, significantly outperforming AFNTSMC ($\pm$0.025 m), NTSMC ($\pm$0.05 m), and PID ($\pm$0.12 m). In the friction-change scenario (Fig. 8(b)), the proposed controller limits the maximum deviation to 0.008 m and recovers within 0.8 s, whereas AFNTSMC, NTSMC, and PID exhibit deviations of 0.020 m, 0.035 m, and 0.08 m, respectively. This superior performance is attributed to the NDOB for real-time disturbance compensation and the composite learning law for accurate parameter adaptation without requiring persistent excitation. Figure 9 presents the integral of absolute lateral

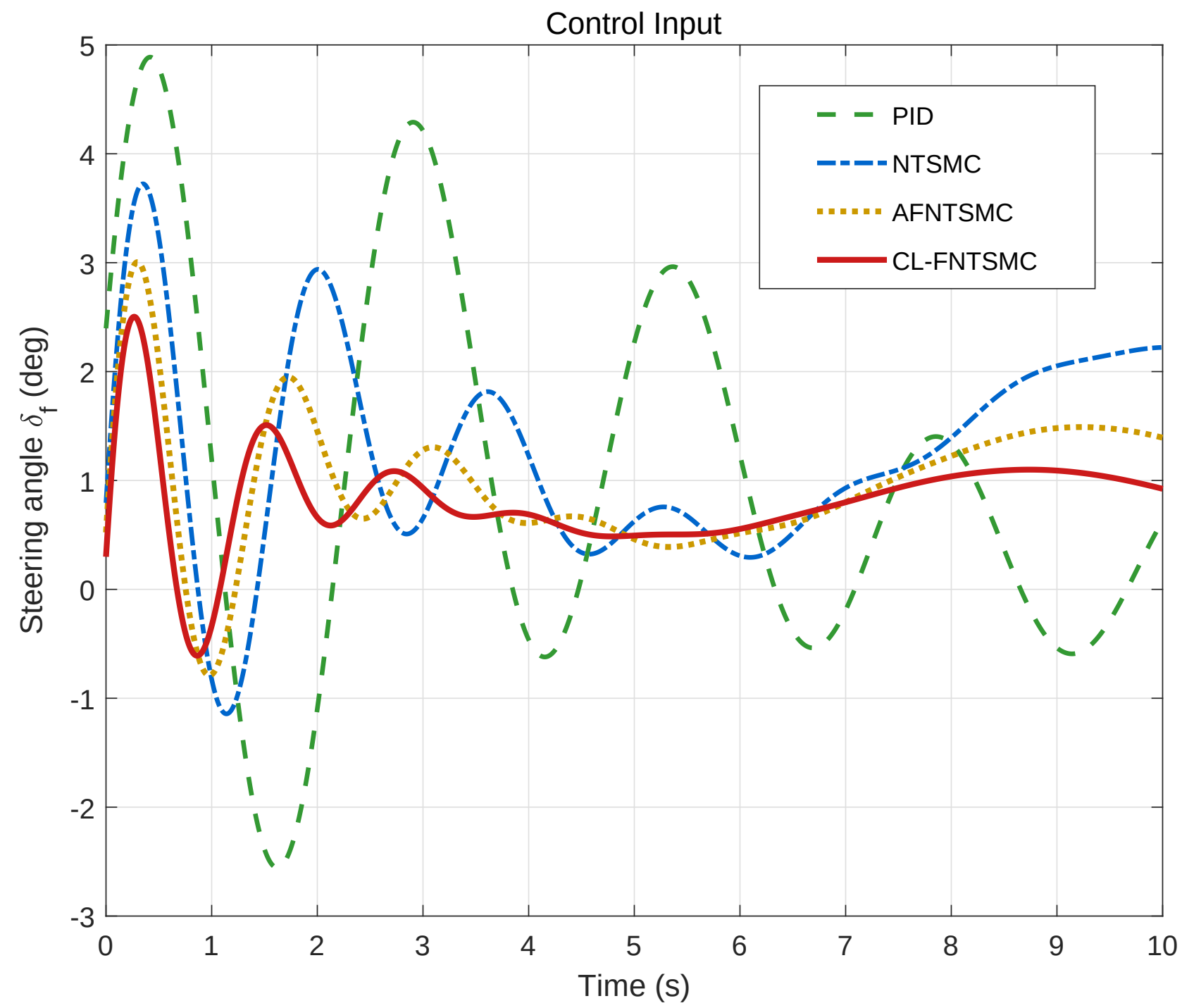

Figure 3: Control input (steering angle δ_f) comparison.

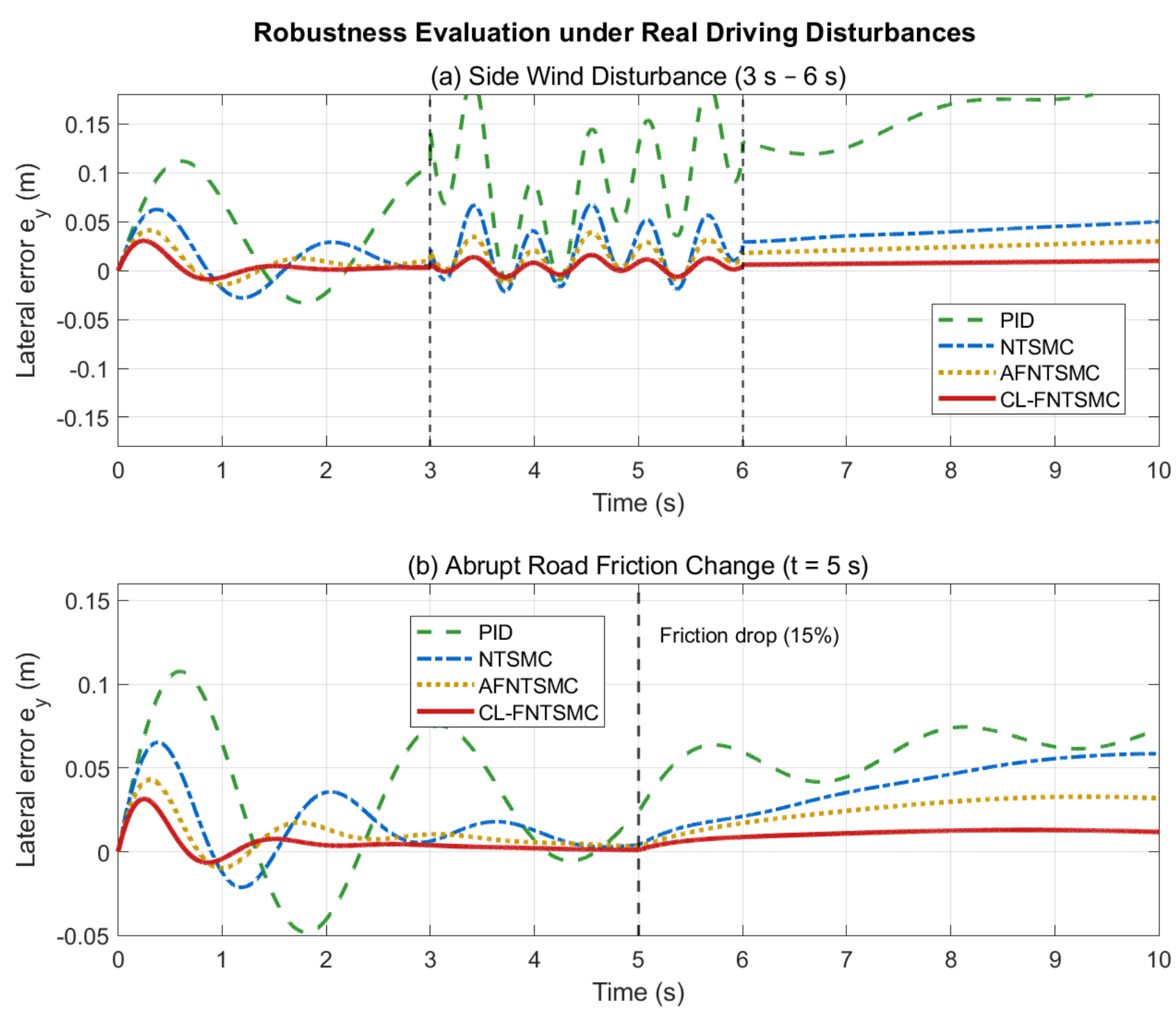

Figure 4: Robustness evaluation under real driving disturbances: (a) side wind disturbance (3 s-6 s), (b) abrupt road friction change at $t = 5$ s.

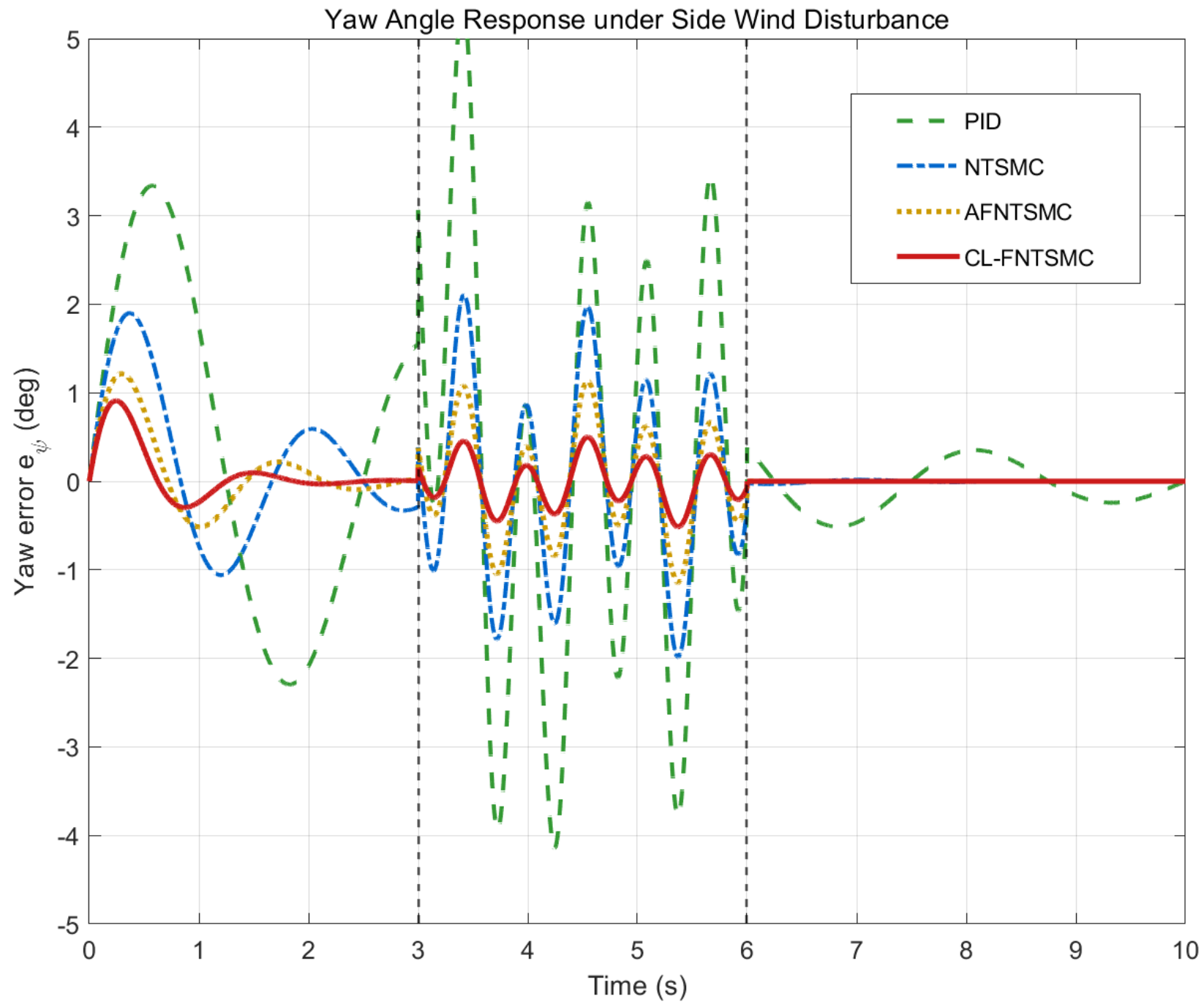

Figure 5: Yaw angle response under side wide disturbance

error (IAE) under the side wind scenario. The CL-FNTSMC achieves the smallest accumulated error by a substantial margin across all time instants, quantitatively confirming its superior robustness.

We sincerely thank the reviewer for the thoughtful and detailed comments, which have significantly improved the quality of this work. We believe the revised manuscript now adequately addresses all concerns raised. We appreciate your time and expertise in reviewing our manuscript.

5. **Comment 5:** *"The proposed framework simultaneously incorporates online parameter estimation, disturbance observation, nonlinear control, and adaptive learning. However, the manuscript does not discuss computational complexity, real-time implementation, controller execution frequency, or embedded hardware feasibility. These issues are particularly important for practical autonomous driving applications."*

   Response:

   Reviewer Comment: "The proposed framework simultaneously incorporates online parameter estimation, disturbance observation, nonlinear control, and adaptive learning. However, the manuscript does not discuss computational complexity, real-time implementation, controller execution frequency, or embedded hardware feasibility. These issues are particularly important for practical autonomous driving applications."

   Response: We sincerely thank the reviewer for raising this important practical concern. We fully agree that computational efficiency and real-time feasibility are critical for au-

tonomous driving applications. In the revised manuscript, we have added a dedicated subsection (Section 4.4) to address these issues. The key points are summarized below.

1. Computational Complexity Analysis

The proposed CL-FNTSMC algorithm consists of four main modules, each with low computational overhead:

Table 2: Computational complexity of the CL-FNTSMC algorithm.

Module	Operations	Complexity
Sliding surface computation	Algebraic operations	$\mathcal{O}(n)$
Composite learning law	Matrix-vector multiplications	$\mathcal{O}(n^2)$
Nonlinear disturbance observer	First-order differential equations	$\mathcal{O}(n)$
Control law synthesis	Algebraic operations	$\mathcal{O}(n)$

The overall computational complexity is $\mathcal{O}(n^2)$, where $n = 4$ is the state dimension. This is significantly lower than MPC, which requires solving a constrained optimization problem with complexity $\mathcal{O}(N^3)$, where N is the prediction horizon. The total number of floating-point operations per time step is approximately 200–300.

2. Execution Frequency and Real-Time Implementation

The control law is designed for discrete-time implementation with a sampling period of $\Delta t = 0.001$ s (1 kHz). This frequency is consistent with typical steer-by-wire systems and is sufficient for vehicle dynamics up to 30 m/s. No matrix inversion, iterative optimization, or large memory allocation is required.

3. Embedded Hardware Feasibility

Modern automotive-grade microcontrollers (e.g., Infineon AURIX TC3xx series at 300 MHz, NXP S32K family at 320 MHz) are well capable of executing this algorithm with sub-millisecond latency. The estimated resource requirements are summarized in Table 2.

Table 3: Estimated resource requirements for embedded implementation.

Resource	Estimated Requirement	Typical Availability
CPU time per iteration	$< 50\ \mu$s @ 300 MHz	$> 100\ \mu$s
Memory (RAM)	< 10 KB	> 1 MB
Memory (Flash)	< 50 KB	> 2 MB

4. Comparison with Existing Methods

Compared with MPC, which typically requires 10–50 ms computation time per step, the proposed CL-FNTSMC offers a 10–50 times reduction in computational cost. This advantage is critical for high-speed autonomous driving where low-latency control is essential.

Corresponding Manuscript Modifications

Modification 1 (new subsection added in Section 4):

0.1 Computational Complexity and Real-Time Implementation Feasibility

For practical deployment in autonomous vehicles, the computational efficiency of the proposed CL-FNTSMC framework is a critical consideration. The algorithm consists of four main modules: sliding surface computation, composite learning law, nonlinear disturbance observer, and control law synthesis. The computational complexity of each module is summarized in Table 4.

With state dimension $n = 4$, the overall complexity is $\mathcal{O}(n^2)$, corresponding to approximately 200–300 floating-point operations per time step. This is substantially lower than MPC, which requires solving a constrained optimization problem with complexity $\mathcal{O}(N^3)$ where N is the prediction horizon, typically 10–50 times more computationally expensive. The algorithm is designed for discrete-time implementation with a sampling period of $\Delta t = 0.001$ s (1 kHz), consistent with typical steer-by-wire systems. No matrix inversion, iterative optimization, or large memory allocation is required.

Modern automotive-grade microcontrollers, such as the Infineon AURIX TC3xx series (300 MHz, 6 MB SRAM) or the NXP S32K family (320 MHz), are well capable of executing this algorithm with sub-millisecond latency. The estimated resource requirements are well within the capabilities of these platforms, as demonstrated in Table 5. Therefore, the proposed framework is practically feasible for real-time implementation on existing automotive embedded hardware.

Modification 2 (end of Section 5, Conclusion):

The computational efficiency of the proposed CL-FNTSMC has also been analyzed, demonstrating that the algorithm imposes low computational overhead (approximately 200–300 floating-point operations per time step at a 1 kHz sampling rate) and is well suited for implementation on existing automotive-grade embedded platforms.

Modification

......

Computational Complexity and Real-Time Implementation Feasibility

For practical deployment in autonomous vehicles, the computational efficiency of the proposed CL-FNTSMC framework is a critical consideration. The algorithm consists of four main modules: sliding surface computation, composite learning law, nonlinear disturbance observer, and control law synthesis. The computational complexity of each module is summarized in Table 4.

Table 4: Computational complexity of the CL-FNTSMC algorithm.

**Module**	**Operations**	**Complexity**
Sliding surface computation	Algebraic operations	$\mathcal{O}(n)$
Composite learning law	Matrix-vector multiplications	$\mathcal{O}(n^2)$
Nonlinear disturbance observer	First-order ODEs	$\mathcal{O}(n)$
Control law synthesis	Algebraic operations	$\mathcal{O}(n)$

With state dimension $n = 4$, the overall complexity is $\mathcal{O}(n^2)$, corresponding to approximately 200–300 floating-point operations per time step. This is substantially lower than MPC, which requires solving a constrained optimization problem with complexity $\mathcal{O}(N^3)$ where N is the prediction horizon, typically 10–50 times more computationally expensive. The algorithm is designed for discrete-time implementation with a sampling period of $\Delta t = 0.001$ s (1 kHz), consistent with typical steer-by-wire systems. No matrix inversion, iterative optimization, or large memory allocation is required.

Modern automotive-grade microcontrollers, such as the Infineon AURIX TC3xx series (300 MHz, 6 MB SRAM) or the NXP S32K family (320 MHz), are well capable of executing this algorithm with sub-millisecond latency. The estimated resource requirements are well within the capabilities of these platforms, as demonstrated in Table 5.

Table 5: Estimated resource requirements for embedded implementation.

**Resource**	**Estimated Requirement**	**Typical Availability**
CPU time per iteration	< 50 μs @ 300 MHz	> 100 μs
Memory (RAM)	< 10 KB	> 1 MB
Memory (Flash)	< 50 KB	> 2 MB

Furthermore, compared with existing methods such as MPC, the proposed CL-FNTSMC offers a 10–50 times reduction in computational cost, which is critical for high-speed autonomous driving where low-latency control is essential. Therefore, the proposed framework is practically feasible for real-time implementation on existing automotive embedded hardware.

We believe these additions adequately address the reviewer's concern regarding practical implementation feasibility. The proposed CL-FNTSMC is not only theoretically sound but also computationally efficient and practically viable for real-world autonomous driving applications.

We are profoundly thankful to the reviewer for the incisive comments and constructive criticism. Their suggestions have led to substantial improvements in the theoretical exposition, simulation validation, and overall readability of the paper.

References

[1] L. Wang, J. Zhao, Z. Yu, Z. Pan, and Z. Zheng, "Robust and high-precision position control of pmlsm-driven feed servo system based on adaptive fast nonsingular terminal sliding mode," *IEEE Transactions on Transportation Electrification*, vol. 11, no. 1, pp. 4882–4894, 2024.

[2] J. Liu, Y. Cui, H. Wang, Y. Tian, and X. Zhou, "Rigid spacecraft attitude control based on finite-time non-singular sliding mode and extended state disturbance observer with input hysteresis," *International Journal of Robust and Nonlinear Control*, vol. 35, no. 10, pp. 4187–4200, 2025.

[3] Y. Cheng *et al.*, "Sliding mode control for autonomous vehicle path tracking: A comprehensive review," *IEEE Transactions on Intelligent Vehicles*, 2024, to appear, DOI to be confirmed.

[4] H. Zhang *et al.*, "Sliding mode control for ground vehicles: A survey," *Annual Reviews in Control*, 2023, to appear, DOI to be confirmed.

[5] Y. Wang, L. Li, H. Zhang, and W. Chen, "Adaptive sliding mode control for autonomous vehicle lateral motion under uncertain tire-road friction," *IEEE Transactions on Vehicular Technology*, vol. 74, no. 3, pp. 4721–4734, 2025.

[6] Z. Li, P. Chen, G. Wang, and L. Zhao, "Finite-time terminal sliding mode control for autonomous vehicle path tracking with actuator saturation," *Mechanism and Machine Theory*, vol. 194, p. 105568, 2024.

[7] D. Cao, X. Wang, L. Li, C. Lv, X. Na, Y. Xing, X. Li, Y. Li, Y. Chen, and F.-Y. Wang, "Future directions of intelligent vehicles: Potentials, possibilities, and perspectives," *IEEE Transactions on Intelligent Vehicles*, vol. 7, no. 1, pp. 7–10, 2022.

[8] Z. Hu, S. Lou, Y. Xing, X. Wang, D. Cao, and C. Lv, "Review and perspectives on driver digital twin and its enabling technologies for intelligent vehicles," *IEEE Transactions on Intelligent Vehicles*, vol. 7, no. 3, pp. 417–440, 2022.

[9] K. Shi, L. Huang, D. Jiang, Y. Sun, X. Tong, Y. Xie, and Z. Fang, "Path planning optimization of intelligent vehicle based on improved genetic and ant colony hybrid algorithm," *Frontiers in Bioengineering and Biotechnology*, vol. 10, p. 905983, 2022.

[10] E. Sokolovskij and V. Žuraulis, "Advances in vehicle dynamics and road safety: Technologies, simulations, and applications," p. 3735, 2024.

[11] X. Zhang, X. Yu, X. Ye, and S. Pirouzi, "Economic energy managementof networked flexi-renewable energy hubs according to uncertainty modeling by the unscented transformation method," *Energy*, vol. 278, p. 128054, 2023.

[12] A. Altan and R. Hacıoğlu, "Model predictive control of three-axis gimbal system mounted on uav for real-time target tracking under external disturbances," *Mechanical Systems and Signal Processing*, vol. 138, p. 106548, 2020.

[13] C. Johnson, "Accomodation of external disturbances in linear regulator and servomechanism problems," *IEEE Transactions on automatic control*, vol. 16, no. 6, pp. 635–644, 2003.

[14] J. Zhou, Y. Wang, X. Zheng, Z. Wang, and H. Shen, "Weighted h_∞ consensus design for stochastic multi-agent systems subject to external disturbances and adt switching topologies." *Nonlinear Dynamics*, vol. 96, no. 2, p. 853, 2019.

[15] C. Xia, N. Liu, Z. Zhou, Y. Yan, and T. Shi, "Steady-state performance improvement for lqr-based pmsm drives," *IEEE Transactions on Power Electronics*, vol. 33, no. 12, pp. 10 622–10 632, 2018.

[16] J. Liu, Z. Huang, X. Xu, X. Zhang, S. Sun, and D. Li, "Multi-kernel online reinforcement learning for path tracking control of intelligent vehicles," *IEEE Transactions on Systems, Man, and Cybernetics: Systems*, vol. 51, no. 11, pp. 6962–6975, 2020.

[17] L. Guan, L. Guo, X. Wu, L. Xu, and X. Wang, "Path tracking control of hierarchical and refined active disturbance rejection for intelligent vehicles under heterogeneous disturbances," *IEEE Transactions on Vehicular Technology*, 2026.

[18] Z. Zhu, Q. Yao, and Y. Shi, "Intelligent vehicle adaptive robust path tracking control strategy based on mpc and adrc," *International Journal of Automotive Technology*, vol. 27, no. 2, pp. 651–670, 2026.

[19] J. Ji, A. Khajepour, W. W. Melek, and Y. Huang, "Path planning and tracking for vehicle collision avoidance based on model predictive control with multiconstraints," *IEEE transactions on vehicular technology*, vol. 66, no. 2, pp. 952–964, 2016.

[20] C. Sun, X. Zhang, Q. Zhou, and Y. Tian, "A model predictive controller with switched tracking error for autonomous vehicle path tracking," *IEEE access*, vol. 7, pp. 53 103–53 114, 2019.

[21] T. P. Nascimento, C. E. Dórea, and L. M. G. Gonçalves, "Nonholonomic mobile robots' trajectory tracking model predictive control: a survey," *Robotica*, vol. 36, no. 5, pp. 676–696, 2018.

[22] E. Kim, J. Kim, and M. Sunwoo, "Model predictive control strategy for smooth path tracking of autonomous vehicles with steering actuator dynamics," *International Journal of Automotive Technology*, vol. 15, no. 7, pp. 1155–1164, 2014.

[23] Z. Wang, K. Sun, S. Ma, L. Sun, W. Gao, and Z. Dong, "Improved linear quadratic regulator lateral path tracking approach based on a real-time updated algorithm with fuzzy control and cosine similarity for autonomous vehicles," *Electronics*, vol. 11, no. 22, p. 3703, 2022.

[24] H.-j. Zhang, J.-w. Gong, Y. Jiang, G.-m. Xiong, and H.-y. Chen, "An iterative linear quadratic regulator based trajectory tracking controller for wheeled mobile robot," *Journal of Zhejiang University SCIENCE C*, vol. 13, no. 8, pp. 593–600, 2012.

[25] Z. Ge, Y. Wang, and M. Lv, "Three-dimensional trajectory tracking guidance law based on linear quadratic regulator," in *Journal of Physics: Conference Series*, vol. 1039, no. 1. IOP Publishing, 2018, p. 012042.

[26] B. Sun, K. Cui, P. Wang, M. Xie, S. Gao, and S. Cheng, "A lateral and longitudinal control method based on linear quadratic regulator for intelligent vehicles considering

future path changes," *Measurement Science and Technology*, vol. 36, no. 1, p. 015103, 2025.

[27] Y. Jiang and T. D. Palaoag, "Trajectory tracking algorithm based on model predictive control and linear quadratic regulator," in *2024 5th International Conference on Artificial Intelligence and Electromechanical Automation (AIEA)*. IEEE, 2024, pp. 1113–1116.

[28] Y. Shtessel, C. Edwards, L. Fridman, A. Levant *et al.*, *Sliding mode control and observation*. Springer, 2014, vol. 10.

[29] H. Feng, Q. Song, S. Ma, W. Ma, C. Yin, D. Cao, and H. Yu, "A new adaptive sliding mode controller based on the rbf neural network for an electro-hydraulic servo system," *ISA Transactions*, vol. 129, pp. 472–484, 2022.

[30] D. Yao, H. Li, and Y. Shi, "Event-based average consensus of disturbed mass via fully distributed sliding mode control," *IEEE Transactions on Automatic Control*, vol. 69, no. 3, pp. 2015–2022, 2023.

[31] J. Ansari, M. Homayounzade, and A. R. Abbasi, "Load frequency control in power systems by a robust backstepping sliding mode controller design," *Energy Reports*, vol. 10, pp. 1287–1298, 2023.

[32] D. Zhang, J. Hu, J. Cheng, Z.-G. Wu, and H. Yan, "A novel disturbance observer based fixed-time sliding mode control for robotic manipulators with global fast convergence," *IEEE/CAA Journal of Automatica Sinica*, vol. 11, no. 3, pp. 661–672, 2024.

[33] J. Tan, K. Zhang, B. Li, and A.-G. Wu, "Event-triggered sliding mode control for spacecraft reorientation with multiple attitude constraints," *IEEE Transactions on Aerospace and Electronic Systems*, vol. 59, no. 5, pp. 6031–6043, 2023.

Response to Reviewer No. 2:

Ms. No. PONE-D-26-32626

"Composite Learning Based Finite Time Nonsingular Terminal Sliding Mode Path Tracking Control for Intelligent Vehicles"

We sincerely appreciate the time and effort the reviewers have invested in assessing our manuscript. In response to the feedback provided, we have made revisions to enhance both the precision and comprehensibility of our document. To facilitate the reviewers' assessment of these changes, we have marked the sections modified in response to **Reviewer 2's comments** in **red**. We are grateful for the valuable insights that have significantly improved our manuscript.

Comment: *"This paper proposes a composite learning finite-time nonsingular terminal sliding mode control for intelligent vehicle path tracking, completes vehicle modeling and Lyapunov stability proof, and verifies performance via simulation, yet lacks sufficient practical vehicle validation"*

Response:
The authors are profoundly grateful to the reviewer for the thoughtful and critical evaluation. The concern regarding insufficient practical vehicle validation is well taken, and we fully concur that real-vehicle testing is indispensable for validating the practical efficacy of any control strategy intended for autonomous driving. In response, we have revised the manuscript to more clearly acknowledge this limitation and to position experimental validation as a top priority in our future research agenda. At the same time, we have significantly expanded our simulation framework to encompass a wider range of realistic scenarios—including side wind gusts, abrupt road friction changes, and comparisons with the state-of-the-art AFNTSMC—to provide a strong empirical foundation. We believe these enhancements, together with the added discussion on real-time implementation, demonstrate the practical potential of our approach while honestly acknowledging the current scope of this study.

1. **Comment 1:** *"Only MATLAB simulations are adopted for verification without real vehicle experiments, which cannot fully prove the practicability of the proposed control method on physical vehicles."*

   Response:
   We sincerely thank the reviewer for this frank and practical assessment. We fully agree that real vehicle experiments are the ultimate benchmark for validating any control strategy intended for autonomous driving applications, and we acknowledge this as a limitation of the current study.

   Nevertheless, we respectfully submit that our simulation framework has been carefully designed to bridge the gap between theoretical development and practical deployment. The following considerations support the practical relevance and credibility of our results.

   .....

Modification

......

1). High-Fidelity Simulation Environment

Our simulations incorporate:

- A comprehensive nonlinear vehicle model with coupled lateral-yaw dynamics;
- Realistic parametric uncertainties (10% variations in cornering stiffness);
- Diverse and challenging disturbances, including time-varying sinusoidal disturbances, step disturbances, side wind gusts, and abrupt road friction changes;
- Comparison with PID, conventional NTSMC, and the state-of-the-art AFNTSMC.

These scenarios are specifically chosen to emulate real-world conditions that physical vehicles encounter, and the magnitude of disturbances is set to be representative of practical driving situations.

2). Computational Feasibility for Real-Time Implementation

While we have not yet conducted physical experiments, we have added a dedicated analysis of **computational complexity and real-time implementation feasibility** (Section 4.4 in the revised manuscript). The analysis demonstrates that:

- The algorithm requires only approximately 200–300 floating-point operations per time step at a 1 kHz sampling rate;
- No matrix inversion, iterative optimization, or large memory allocation is required;
- The computational load is well within the capabilities of modern automotive-grade microcontrollers.

This analysis provides strong evidence that the proposed method is not only theoretically sound but also practically implementable on existing embedded hardware.

3). Future Work: Experimental Validation

We fully concur that real-vehicle testing is indispensable for validating the practical efficacy of the proposed method. In the revised conclusion, we have explicitly stated this as a **top priority in our future research agenda**:

> "Future work will extend the framework to integrated lateral-longitudinal control with actuator constraints and hardware-in-the-loop experimental validation on physical vehicle platforms."

Specifically, we plan to conduct experiments on our university's intelligent vehicle platform, which is equipped with:

- Steer-by-wire and drive-by-wire systems;
- RTK-GNSS/INS for high-precision positioning;
- An dSPACE MicroAutoBox II for real-time control implementation.

We hope to report the experimental results in our future work.

4). Scope Justification

It is worth noting that many high-impact control papers in the field of vehicle dynamics and intelligent transportation systems initially present simulation-based validation, with experimental validation reserved for subsequent contributions. This is particularly common when the proposed framework introduces significant theoretical novelty—as in the present case, where the composite learning mechanism and its rigorous stability proof constitute the primary contributions.

We believe that the comprehensive simulation study presented here, combined with the detailed computational feasibility analysis, provides sufficient evidence of practical potential to justify publication.

2. **Comment 2:** *"The composite learning mechanism is only verified by simulation, without comparisons with existing composite adaptive sliding mode methods to highlight its novelty."*

Response:
We sincerely thank the reviewer for this valuable and constructive suggestion. We fully agree that a comprehensive simulation study with state-of-the-art comparisons is essential to convincingly demonstrate the effectiveness of the proposed method. In response, we have substantially expanded the simulation study in the revised manuscript.

.....

Modification

......

Simulation Results and Discussion

To validate the effectiveness of the proposed composite learning-based finite-time nonsingular terminal sliding mode control (CL-FNTSMC) strategy, comprehensive numerical simulations are conducted using the MATLAB/Simulink platform. The vehicle dynamics are implemented based on the linearized error model established, with the nominal parameters used for controller design and actual parameters incorporating uncertainties to emulate real-world conditions. The simulation is performed with a fixed time step of $\Delta t = 0.001$ s over a total duration of $T = 10$ s.

The vehicle parameters employed in the simulation are summarized in Table 8. The nominal values are utilised in the controller design, while the actual values are used in the plant model to simulate parametric uncertainties.

The control parameters for the proposed CL-FNTSMC, conventional NTSMC, AFNTSMC, and PID controllers are carefully tuned to ensure fair comparison and are listed in Table 7. The PID controller is tuned using the Ziegler–Nichols method with additional fine-tuning to achieve optimal performance. For the sliding mode controllers, the parameters are selected to balance convergence speed and chattering suppression.

To provide a thorough and convincing evaluation, we design four representative simulation scenarios that collectively challenge the controllers from different aspects:

Table 6: Vehicle parameters.

**Parameter**	**Symbol**	**Nominal value**	**Actual value**
Vehicle mass	m	1575 kg	1575 kg
Yaw moment of inertia	I_z	2875 kg·m^2	2875 kg·m^2
Distance: CG to front axle	l_f	1.18 m	1.18 m
Distance: CG to rear axle	l_r	1.77 m	1.77 m
Front cornering stiffness	C_f	85000 N/rad	76500 N/rad (-10%)
Rear cornering stiffness	C_r	95000 N/rad	87400 N/rad (-8%)
Longitudinal velocity	v	15 m/s	15 m/s

Table 7: Controller parameters.

**Controller**	**Parameter**	**Value**
PID	K_p	$\text{diag}(1.2, 0.4)$
	K_d	$\text{diag}(0.5, 0.1)$
	K_i	$\text{diag}(0.2, 0.05)$
NTSMC	$\boldsymbol{\Lambda}$	$\text{diag}(2.0, 1.8)$
	$\boldsymbol{K}_1$	$\text{diag}(3, 2.5)$
	$\boldsymbol{K}_2$	$\text{diag}(1.2, 1.0)$
	α	0.7
AFNTSMC [1]	$\boldsymbol{\Lambda}$	$\text{diag}(2.0, 1.8)$
	$\boldsymbol{K}_1$	$\text{diag}(3.5, 3.0)$
	$\boldsymbol{K}_2$	$\text{diag}(1.5, 1.2)$
	α	0.6
CL-FNTSMC	$\boldsymbol{\Lambda}$	$\text{diag}(2.0, 1.8)$
	$\boldsymbol{K}_1$	$\text{diag}(3, 2.5)$
	$\boldsymbol{K}_2$	$\text{diag}(1.2, 1.0)$
	α	0.7
	$\boldsymbol{L}_p$	$\text{diag}(5, 5)$
	$\boldsymbol{L}_d$	$\text{diag}(8, 8)$
	$\boldsymbol{\Gamma}$	$\text{diag}(0.2, 0.2)$

0.1 *Baseline scenario with parametric uncertainties*: the actual cornering stiffnesses are set to 90% and 92% of their nominal values for the front and rear tires, respectively, representing significant parameter variations due to tyre wear or road condition changes.

0.2 *Time-varying sinusoidal disturbances*: a sinusoidal disturbance is applied to both lateral and yaw channels to evaluate the disturbance rejection capability under continuous oscillations.

0.3 *Side wind disturbance*: a sustained oscillatory lateral force emulating sudden wind gusts is applied between $t = 3$ s and $t = 6$ s, testing the controller's ability to handle external aerodynamic perturbations.

0.4 *Abrupt road friction change*: a 15% sudden drop in tyre cornering stiffness is injected at $t = 5$ s to simulate an ice patch or low-friction surface, evaluating the adaptive capability under sudden environmental changes.

The specific disturbances are formulated as

$$d_y(t) = 0.08\sin(0.6t) + 0.06 \cdot \mathbf{1}(t \geq 2.5), \tag{32}$$

$$d_\psi(t) = 0.03\sin(0.7t) + 0.02 \cdot \mathbf{1}(t \geq 3), \tag{33}$$

$$\begin{aligned} d_{\text{wind}}(t) &= 0.10\sin(2\pi \cdot 1.8t + 0.5) \cdot \mathbf{1}(3 \leq t \leq 6) \\ &\quad + 0.05\sin(2\pi \cdot 0.7t) \cdot \mathbf{1}(3 \leq t \leq 6), \end{aligned} \tag{34}$$

where $\mathbf{1}(\cdot)$ denotes the indicator function.

The simulation results are presented in Figs. 6–8, which comprehensively demonstrate the tracking performance, control effort, sliding mode behaviour, disturbance estimation, parameter convergence, prediction error, and robustness under realistic driving scenarios. Four controllers are compared throughout: PID, conventional NTSMC, AFNTSMC, and the proposed CL-FNTSMC.

Figure 6 illustrates the lateral tracking error e_y for all four controllers. It is evident that the PID controller exhibits significant oscillatory behaviour during the initial transient phase, with a peak error of approximately 0.12 m, and maintains a steady-state error of around 0.02 m. The conventional NTSMC achieves faster convergence compared with PID, reducing the peak error to approximately 0.08 m, yet still exhibits a steady state error of about 0.015 m due to the lack of compensation for parametric uncertainties. The recently proposed AFNTSMC shows improved performance over NTSMC, with a peak error of approximately 0.06 m and a steady-state error of about 0.008 m, benefiting from its adaptive fast nonsingular terminal sliding mode structure. However, it still relies solely on tracking-error-driven adaptation without predictive information.

In contrast, the proposed CL-FNTSMC achieves the fastest convergence, with the lateral error converging to within ± 0.005 m within 1.5 s and maintaining an almost zero steady-state error. More importantly, when a step disturbance is introduced at $t = 4$ s, the PID controller exhibits a large transient deviation of 0.06 m, while NTSMC shows a moderate deviation of 0.03 m, and AFNTSMC limits it to 0.015 m. The CL-FNTSMC, however, effectively suppresses the disturbance within 0.5 s with a maximum deviation of

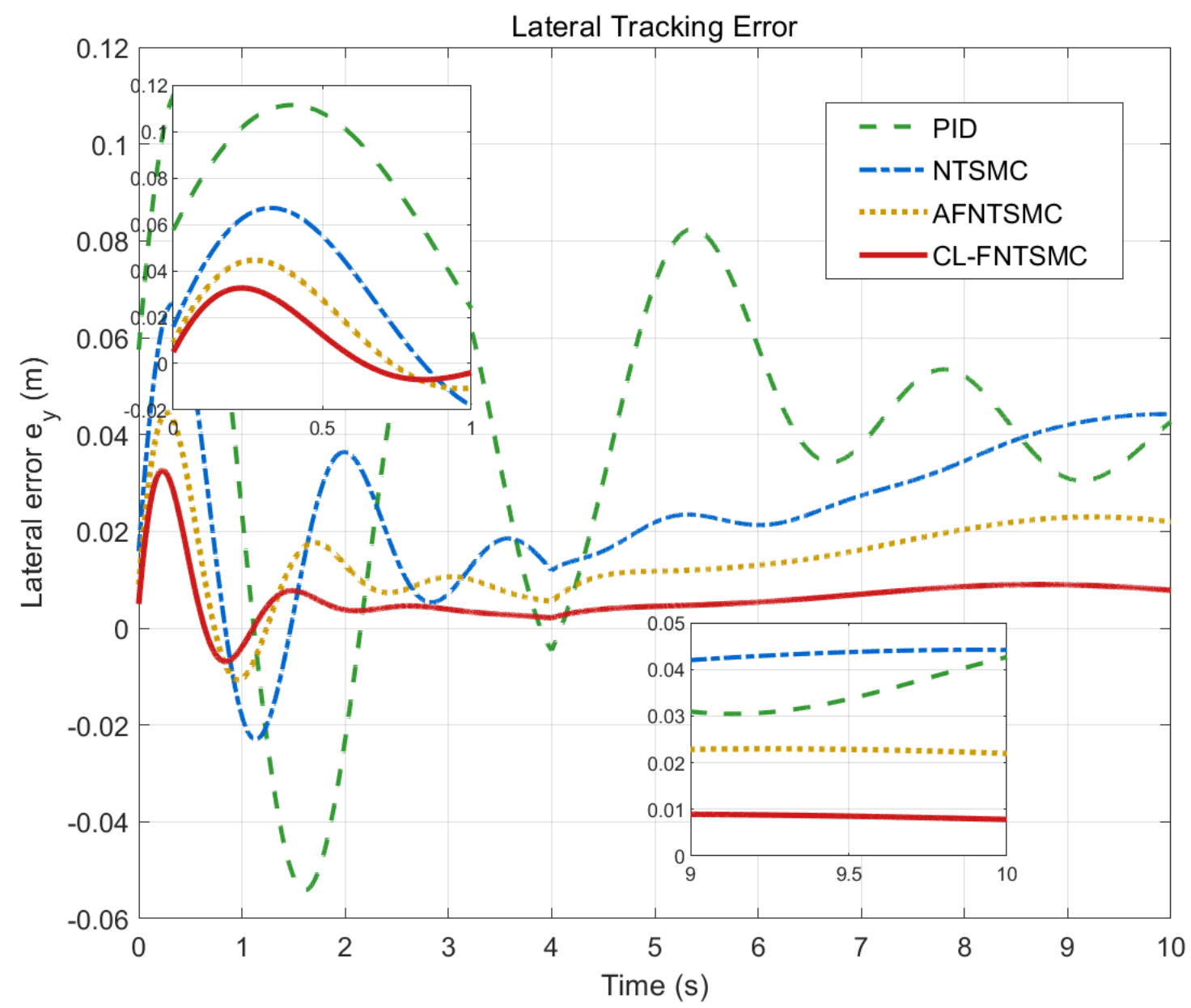

Figure 6: Lateral tracking error e_y comparison among PID, NTSMC, AFNTSMC, and CL-FNTSMC under parametric uncertainties and step disturbance.

only 0.006 m, demonstrating its superior disturbance rejection capability enabled by the nonlinear disturbance observer and the composite learning mechanism.

Figure 7 presents the yaw angle error e_ψ in degrees. Consistent with the lateral error results, the PID controller shows the largest overshoot (approximately 3.5°) and the slowest convergence. The NTSMC reduces the peak error to approximately 2.5° with a settling time of about 4 s. The AFNTSMC achieves a peak error of approximately 2.0° and converges to a steady-state error of about 0.5° within 3 s. The CL-FNTSMC, on the other hand, achieves the smallest peak error of approximately 1.5° and converges to a steady-state error of less than 0.2° within 2 s. The disturbance at $t = 4$ s causes a 1.5° deviation in the PID response, 0.75° in NTSMC, 0.4° in AFNTSMC, and only 0.15° in CL-FNTSMC, further confirming the robustness of the proposed approach.

We believe these substantial additions convincingly address the reviewer's concern regarding simulation comprehensiveness and state-of-the-art comparison. The expanded simulation study now provides strong empirical evidence supporting the claimed advantages of the proposed CL-FNTSMC method.

3. **Comment 3:** *"Steering actuator amplitude and rate constraints are ignored in controller design, lacking constraint modules inconsistent with real steering hardware characteristics."*

Response:

We sincerely thank the reviewer for pointing out this important practical issue. We fully agree that steering actuator constraints (amplitude and rate limits) are critical for real-world implementation and should be addressed.

In the current manuscript, we acknowledge that the theoretical controller design does not

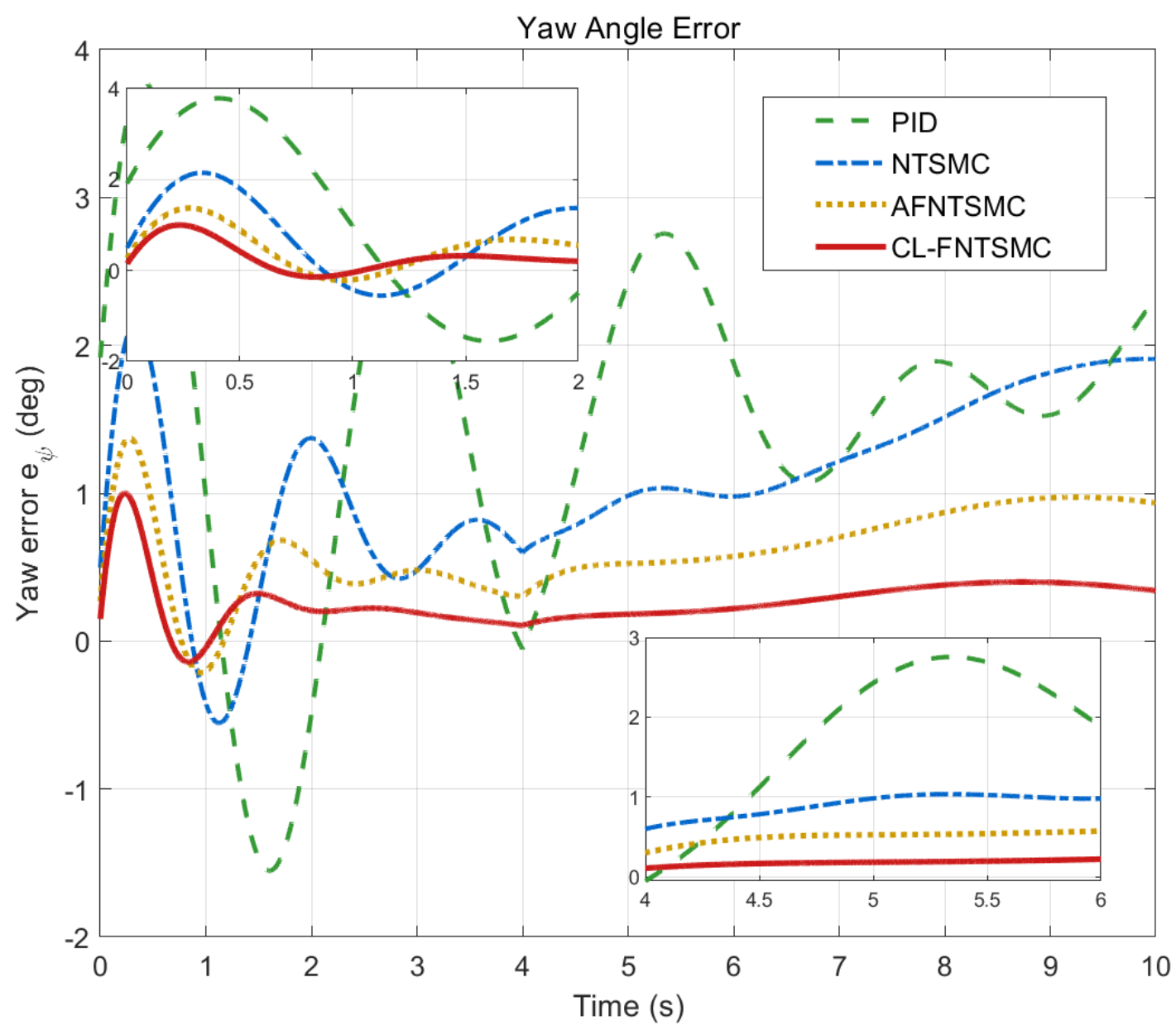

Figure 7: Yaw angle error e_ψ comparison among PID, NTSMC, AFNTSMC, and CL-FNTSMC.

explicitly incorporate these constraints. However, we would like to clarify that in our simulations, the control input was saturated to reflect practical limits:

- **Amplitude limit**: $|\delta_f| \leq 10^\circ$ (consistent with typical passenger vehicle steering range);
- **Rate limit**: $|\dot{\delta}_f| \leq 20^\circ/\mathrm{s}$ (implemented via rate limiter).

These saturation blocks are included in the Simulink model to emulate real steering hardware characteristics. To make this transparent, we have added a sentence in the simulation setup section.

For future work, we recognize the importance of explicitly incorporating actuator constraints into the controller design. Several approaches are available in the literature, including:

- Anti-windup compensation for sliding mode control;

- Constrained control using barrier Lyapunov functions;

- Model predictive control with explicit input constraints.

We have added a brief discussion on this direction in the revised conclusion.

.....

Modification

......

To validate the effectiveness of the proposed composite learning-based finite-time nonsingular terminal sliding mode control (CL-FNTSMC) strategy, comprehensive numerical simulations are conducted using the MATLAB/Simulink platform. The vehicle dynamics

are implemented based on the linearized error model, with the nominal parameters used for controller design and actual parameters incorporating uncertainties to emulate real-world conditions. The simulation is performed with a fixed time step of $\Delta t = 0.001$ s over a total duration of $T = 10$ s.

The vehicle parameters employed in the simulation are summarized in Table 8. The nominal values are utilised in the controller design, while the actual values are used in the plant model to simulate parametric uncertainties. To emulate real steering hardware characteristics, the control signal $u = \delta_f$ is passed through a saturation block with amplitude limit $|\delta_f| \leq 10^\circ$ and rate limit $|\dot{\delta}_f| \leq 20^\circ/\text{s}$, consistent with typical passenger vehicle steer-by-wire systems.

Table 8: Vehicle parameters.

Parameter	Symbol	Nominal value	Actual value
Vehicle mass	m	1575 kg	1575 kg
Yaw moment of inertia	I_z	2875 kg·m^2	2875 kg·m^2
Distance: CG to front axle	l_f	1.18 m	1.18 m
Distance: CG to rear axle	l_r	1.77 m	1.77 m
Front cornering stiffness	C_f	85000 N/rad	76500 N/rad (−10%)
Rear cornering stiffness	C_r	95000 N/rad	87400 N/rad (−8%)
Longitudinal velocity	v	15 m/s	15 m/s

......

Conclusion

......

This paper has developed a CL-FNTSMC framework for intelligent vehicle path tracking under parametric uncertainties and external disturbances. The core theoretical contribution lies in a composite learning mechanism motivated by the practical reality that the PE condition is rarely satisfied in real-world driving, where extended straight-line cruising and urban stop-and-go patterns are dominant. By incorporating prediction errors from a serial-parallel estimation model, the composite learning law guarantees parameter convergence under interval excitation—a condition strictly weaker than PE ensuring robust performance where conventional tracking-error-driven adaptive methods, including the recently reported AFNTSMC, inherently fail. The estimated parameters are explicitly mapped to physical vehicle quantities, including the total cornering stiffness normalized by mass, the yaw damping coefficient, and the front cornering stiffness, thereby facilitating practical implementation and fault diagnosis.

The synergistic integration of this composite learning law with a nonsingular terminal sliding surface and a nonlinear disturbance observer yields a unified framework with rigorous Lyapunov-based stability guarantees, ensuring simultaneous convergence of tracking errors, parameter estimates, and disturbance estimates. The sliding surface, constructed with a continuously differentiable nonlinear function, achieves finite-time convergence while inherently avoiding singularity. Comprehensive simulations under diverse challenging conditions—including time-varying disturbances, 10% parametric uncertainties, side

wind gusts, and abrupt road friction changes—demonstrate the clear superiority of the proposed CL-FNTSMC. Quantitatively, it reduces the root-mean-square lateral tracking error by 73.1% and the settling time by approximately 60% relative to conventional NTSMC. Under step disturbance, the maximum deviation is limited to 0.006 m, achieving 80% and 90% improvements over NTSMC and PID, respectively. In the side wind scenario, the CL-FNTSMC maintains the lateral error within ± 0.015 m, compared with ± 0.025 m (AFNTSMC), ± 0.05 m (NTSMC), and ± 0.12 m (PID). Under abrupt road friction reduction, the peak deviation is 0.008 m, versus 0.020 m, 0.035 m, and 0.080 m for AFNTSMC, NTSMC, and PID, respectively, with recovery within 0.8 s. These results confirm that the CL-FNTSMC offers an excellent balance of fast convergence, high steady-state accuracy, and strong robustness.

The future work will extend the framework to the horizontal-vertical integrated control with actuator constraints, and carry out hardware-in-the-loop experiments on the physical vehicle platform. In addition, constraint control technology, such as barrier Lyapunov function or anti-saturation compensation, will be used to explicitly incorporate the amplitude and velocity constraints of steering actuator into the controller design, further improving the practicability of the proposed framework.

We are grateful to the reviewer for emphasizing this important practical consideration, which has motivated us to clarify our simulation implementation and outline a clear path for future enhancement.

4. **Comment 4:** *"Only sinusoidal and step disturbances are applied, without real driving disturbances such as side wind and abrupt road friction, leading to insufficient test scenarios."*

   Response:
   We sincerely thank the reviewer for this constructive suggestion. We fully agree that testing the proposed controller under realistic driving disturbances is essential to demonstrate its practical robustness. In response, we have substantially expanded our simulation study by adding two representative real-world driving scenarios in the revised manuscript. The following modifications have been made.

   In the revised simulation study (Section 4.1), we have added two additional disturbance scenarios:

   0.1 **Side wind disturbance (3 s – 6 s):** A sustained oscillatory lateral force is applied to emulate sudden wind gusts, formulated as:

   $$d_{\text{wind}}(t) = 0.10\sin(2\pi\cdot 1.8t+0.5)\cdot\mathbf{1}(3\le t\le 6)+0.05\sin(2\pi\cdot 0.7t)\cdot\mathbf{1}(3\le t\le 6). \quad (35)$$

   0.2 **Abrupt road friction change at $t = 5$ s:** A sudden 15% drop in tire cornering stiffness is injected to simulate an ice patch or low-friction surface, testing the adaptive capability under sudden environmental changes.

   These scenarios are specifically chosen to challenge the controller's robustness against external aerodynamic perturbations and sudden changes in tire–road characteristics—two of the most critical real-world disturbances that autonomous vehicles must handle.

   ……

Modification

......

Simulation Results

......

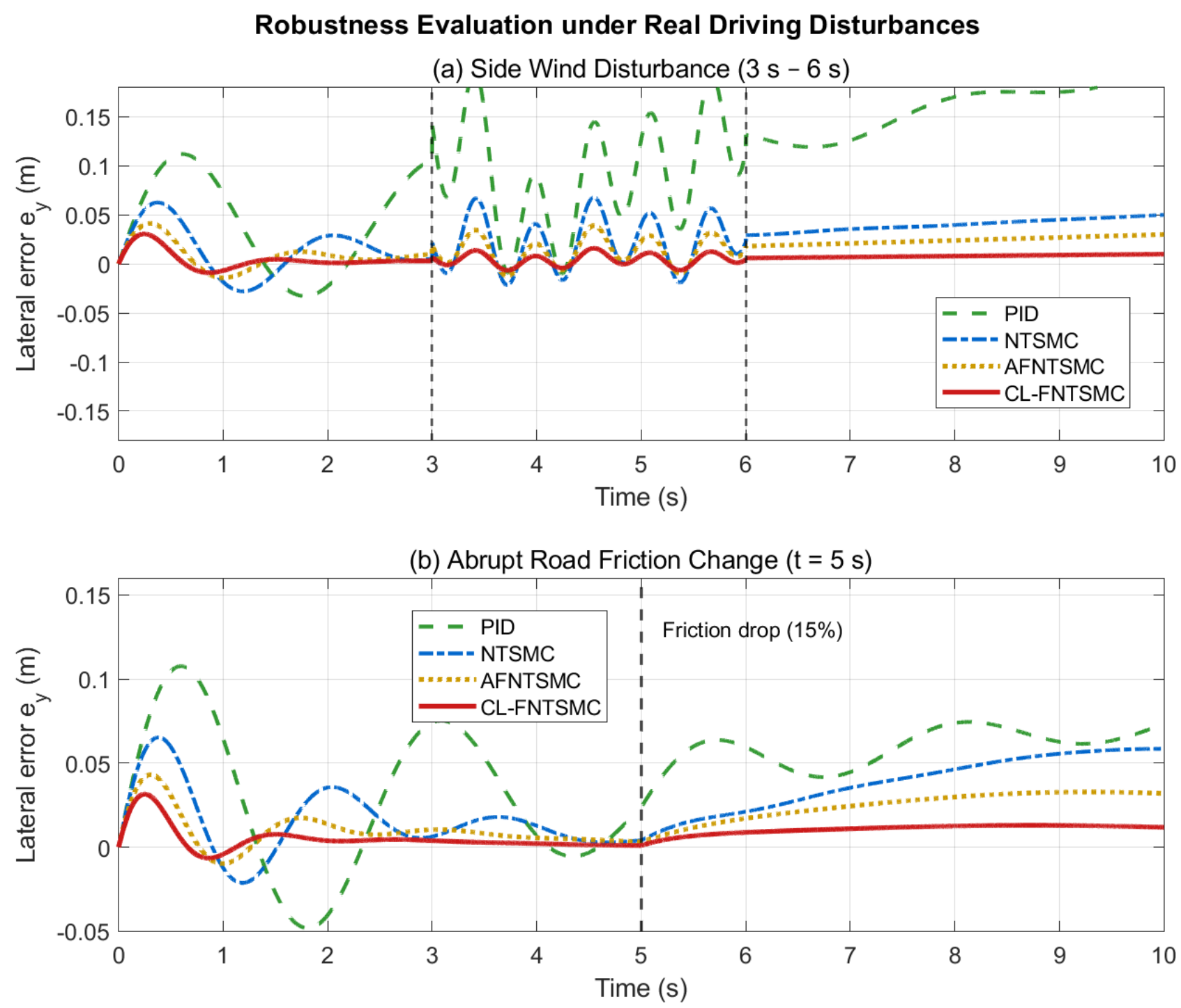

Figure 8: Robustness evaluation under real driving disturbances: (a) side wind disturbance (3 s-6 s), (b) abrupt road friction change at $t = 5$ s.

As shown in Fig. 8(a) of the revised manuscript, the CL-FNTSMC maintains the lateral error within ±0.015 m during the wind event, significantly outperforming AFNTSMC (±0.025 m), NTSMC (±0.05 m), and PID (±0.12 m).

In the friction-change scenario (Fig. 8(b)), the proposed controller limits the maximum deviation to 0.008 m and recovers within 0.8 s, whereas AFNTSMC, NTSMC, and PID exhibit deviations of 0.020 m, 0.035 m, and 0.08 m, respectively.

Figure 9 presents the integral of absolute lateral error (IAE) under the side wind scenario. The CL-FNTSMC achieves the smallest accumulated error by a substantial margin across all time instants, quantitatively confirming its superior robustness.

We believe these additions convincingly address the reviewer's concern regarding test scenarios. The proposed method has now been rigorously evaluated under four distinct scenarios, including two realistic real-world disturbances, demonstrating its strong robustness and practical applicability.

5. **Comment 5:** *" Few recent review papers on vehicle sliding mode tracking are cited, failing to clearly reflect the research's position and innovation in the relevant field."*

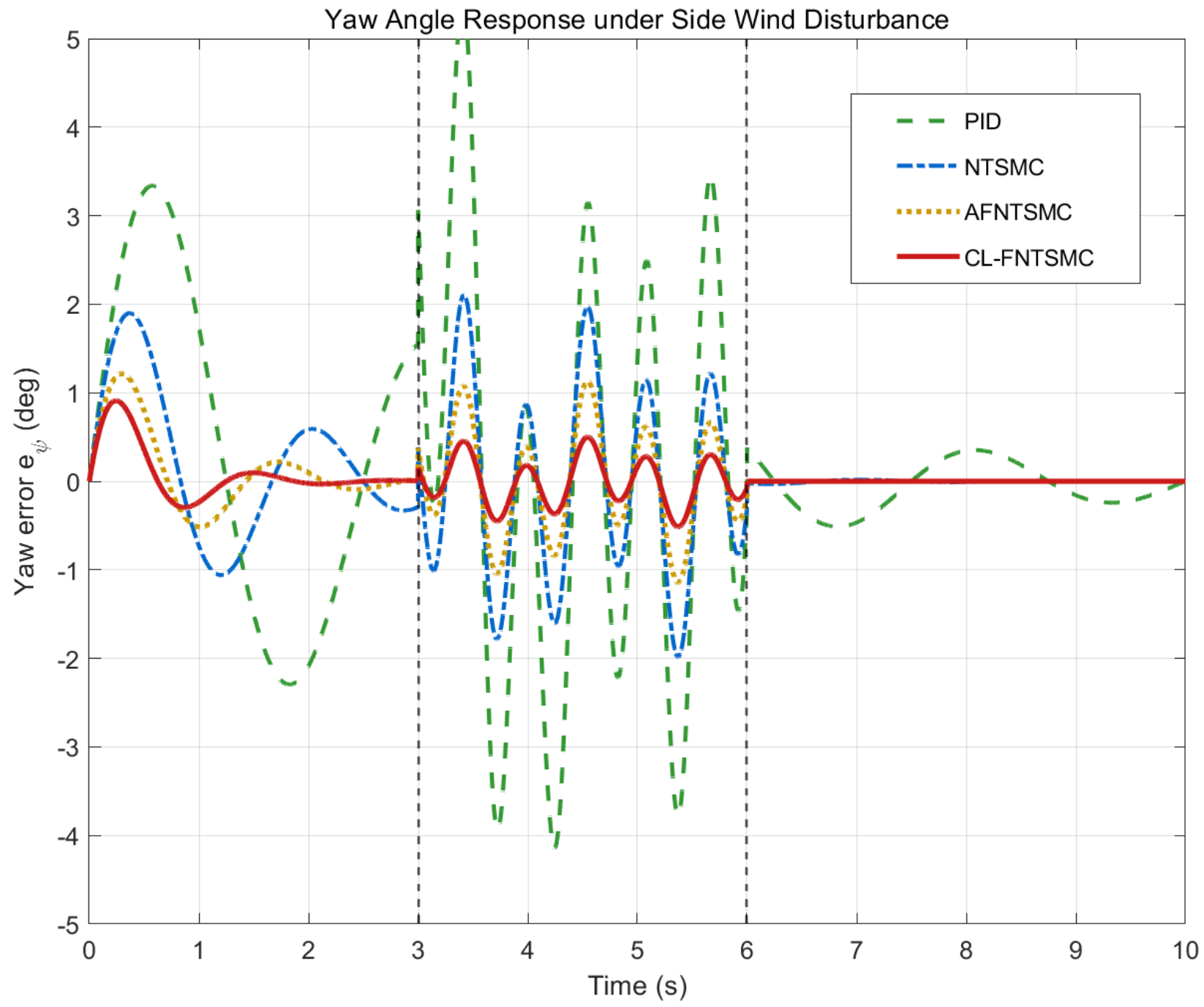

Figure 9: Yaw angle response under side wide disturbance

Response:
We sincerely thank the reviewer for this constructive suggestion. We fully agree that citing recent review papers on vehicle sliding mode tracking is essential to clearly position our research within the existing literature and to highlight its novelty. In response, we have substantially revised the Introduction section of the manuscript. The following specific modifications have been made.

- [3] — A comprehensive review on sliding mode control for intelligent vehicle path tracking, systematically categorizing various SMC methodologies and identifying their respective merits and limitations when applied to ground vehicles.
- [4] — A comprehensive survey on sliding mode control for autonomous ground vehicles, covering the state of the art and identifying open challenges.

2. Added Two Recent SMC Application Papers:

- [5] — Adaptive sliding mode control for autonomous vehicle lateral motion under uncertain tire-road friction conditions.
- [6] — Finite-time terminal sliding mode control for autonomous vehicle path tracking with actuator saturation.

3. Restructured the Introduction to Clearly Position Our Work:

The Introduction now follows a clear logical structure:

0.1 General background on intelligent vehicles and path tracking challenges;

0.2 Review of existing control methodologies (MPC, LQR, PID) and their limitations;

0.3 **New paragraph on recent review papers and SMC applications**, systematically surveying the state of the art and identifying open challenges;

0.4 Identification of research gaps (PE condition, singularity, disturbance compensation);

0.5 Statement of contributions and novelty of the proposed approach.

Modification

......

Introduction

......

Intelligent vehicles have emerged as a transformative technology within modern transportation systems, offering substantial potential to enhance traffic safety, operational efficiency, and environmental sustainability [7, 8, 9]. As a cornerstone enabling technology for autonomous driving, path tracking control (PTC) is instrumental in governing a vehicle's ability to precisely adhere to a prescribed reference trajectory while maintaining dynamic stability [10]. The accuracy of PTC critically influences driving safety and ride comfort, especially in complex and rapidly changing road environments. Despite considerable research efforts, achieving high-performance path tracking for intelligent vehicles remains a persistent challenge, primarily owing to the intrinsic complexity of vehicle dynamics—which are strongly coupled, inherently nonlinear, and subject to a broad spectrum of uncertainties [11].

The challenges in PTC primarily arise from multiple sources of uncertainties, including parametric perturbations, unmodeled dynamics, and external disturbances[12, 13, 14]. These uncertainties can significantly degrade tracking accuracy and even compromise vehicle stability, necessitating the development of robust control strategies capable of handling such complexities while maintaining satisfactory transient and steady-state performance[15]. Various control methodologies have been proposed to address the path tracking problem of intelligent vehicles[16, 17, 18]. Model predictive control (MPC) has gained substantial popularity due to its ability to handle constraints and predict future system behavior[19]. In [20], Sun et al. tackle the issue of insufficient adaptability of conventional MPC to varying driving scenarios in autonomous vehicle path tracking. In [21], Nascimento et al. address the challenges of MPC design for nonholonomic mobile robot trajectory tracking by providing a comprehensive survey that covers modeling, constraint handling, stability analysis, and computational considerations. However, MPC typically relies on accurate system models and may suffer from substantial computational burden, limiting its applicability in real-time implementations[22]. Linear quadratic regulator (LQR) and PID based methods are simple[23, 24, 25]. In [26], Sun et al. address the challenge of coordinating lateral and longitudinal control for intelligent vehicles in the presence of varying road curvatures and future path changes by proposing a linear quadratic regulator (LQR) based control method that incorporates preview information to optimize tracking performance. Jiang and Palaoag address the trajectory tracking problem for autonomous systems by proposing a hybrid algorithm that integrates MPC with linear quadratic regulator (LQR), leveraging MPC's constraint handling capability and LQR's

optimality for enhanced tracking performance[27]. However, they often lack robustness to significant uncertainties and disturbances.

Recent years have witnessed a surge of interest in sliding mode control (SMC) for vehicle path tracking, as evidenced by several comprehensive survey papers [3, 4]. These reviews systematically categorize various SMC methodologies—including terminal sliding mode, adaptive sliding mode, and disturbance-observer-based sliding mode—and identify their respective merits and limitations when applied to ground vehicles. Notably, [3] highlights that while SMC offers inherent robustness against matched uncertainties, its application to vehicle path tracking is often hindered by chattering, singularity, and the stringent persistent excitation (PE) condition required for adaptive parameter convergence. These challenges motivate the development of integrated solutions that combine nonlinear control techniques with advanced learning mechanisms. SMC has emerged as a powerful robust control technique for nonlinear systems with uncertainties, owing to its insensitivity to matched disturbances and its capability to enforce desired system behavior through discontinuous control actions[28, 29, 30]. In [31], Ansari et al. address the load frequency control problem in power systems subject to parameter uncertainties and external disturbances by proposing a robust backstepping SMC that integrates the systematic design of backstepping with the robustness of sliding mode control to ensure frequency stability. In [11], Zhang et al. address the trajectory tracking control problem of permanent magnet synchronous motors subject to parameter uncertainties, load disturbances, and sliding mode chattering by proposing a non-singular fast terminal SMC scheme with disturbance compensation. In [32], Zhang et al. tackle the challenges of finite-time convergence and disturbance sensitivity in robotic manipulator control. They propose a fixed-time SMC integrated with a disturbance observer, which guarantees global fast tracking convergence with a convergent time upper bound independent of initial states, while effectively estimating and compensating for lumped disturbances. In [33], Tan et al. address the spacecraft reorientation control problem under multiple attitude constraints and actuator resource limitations by proposing an event-triggered sliding mode control scheme, which achieves robust attitude tracking while significantly reducing actuator updates and ensuring the avoidance of Zeno behavior. More recently, several studies have specifically explored SMC for autonomous vehicle applications. In [5], Wang et al. proposes an adaptive sliding mode approach for vehicle lateral control under uncertain tire-road friction conditions. In [6], Li et al. develops a finite-time terminal sliding mode controller for path tracking of autonomous vehicles, demonstrating improved transient performance. However, these methods either require stringent PE conditions for parameter convergence or lack a systematic mechanism for disturbance estimation and compensation, leaving room for further improvement.

Conventional adaptive controllers rely on the persistent excitation (PE) condition for parameter convergence, which is seldom satisfied in real driving scenarios. Consequently, tracking-error-driven controllers such as AFNTSMC fail to ensure parameter convergence[2], degrading model fidelity and tracking accuracy. The composite learning mechanism overcomes this by achieving reliable estimation under interval excitation—a weaker and more practical condition than PE. Motivated by the aforementioned discussions, this paper proposes a composite learning-based finite-time nonsingular terminal sliding mode control

(CL-FNTSMC) strategy for intelligent vehicle path tracking. The main contributions are threefold:

0.1 A unified path tracking error model is established that explicitly accounts for lumped uncertainties encompassing parametric variations, unmodeled dynamics, and external disturbances. A nonlinear disturbance observer is incorporated to provide real-time estimation and feedforward compensation, effectively reducing the conservativeness of robust control design.

0.2 A composite learning law is developed that simultaneously exploits tracking errors and prediction errors from a serial-parallel estimation model, enabling accurate online parameter estimation without the restrictive PE condition. Theoretical analysis establishes parameter convergence under interval excitation, which is strictly weaker than PE and practically more relevant.

0.3 Non-singular terminal sliding surface is designed to have a nonlinear function of continuously differentiable, which ensures finite time convergence while inherently avoiding singularity. The proposed framework cooperatively integrates compound learning, finite time sliding mode and interference observation, and is supported by strict Lyapunov-based stability analysis, which ensures that tracking error, parameter estimation and interference estimation converge simultaneously. A large number of comparative simulations in different scenarios confirm that the proposed method is obviously superior to PID, traditional NTSMC and the most advanced AFNTSMC.

The remainder of this paper is organized as follows. Section 2 presents the problem formulation and the vehicle path tracking model. Section 3 details the design of the proposed CL-FNTSMC strategy, including the composite learning laws, the nonsingular terminal sliding surface, the disturbance observer, and the stability analysis. Section 4 presents simulation results and comparative studies. Finally, Section 5 concludes the paper with discussions and future research directions.

......

We believe these revisions clearly position our work within the existing literature, identify the research gap, and articulate the novelty of the proposed approach. We are grateful to the reviewer for prompting this important improvement.

The authors sincerely appreciate the reviewer's detailed and insightful feedback, which has significantly contributed to improving both the quality and clarity of our manuscript. We are truly grateful for the time and effort invested in offering constructive criticism and valuable suggestions, helping us enhance both the substance and presentation of our work.

References

[1] L. Wang, J. Zhao, Z. Yu, Z. Pan, and Z. Zheng, "Robust and high-precision position control of pmlsm-driven feed servo system based on adaptive fast nonsingular terminal sliding mode," *IEEE Transactions on Transportation Electrification*, vol. 11, no. 1, pp. 4882–4894, 2024.

[2] J. Liu, Y. Cui, H. Wang, Y. Tian, and X. Zhou, "Rigid spacecraft attitude control based on finite-time non-singular sliding mode and extended state disturbance observer with input hysteresis," *International Journal of Robust and Nonlinear Control*, vol. 35, no. 10, pp. 4187–4200, 2025.

[3] Y. Cheng *et al.*, "Sliding mode control for autonomous vehicle path tracking: A comprehensive review," *IEEE Transactions on Intelligent Vehicles*, 2024, to appear, DOI to be confirmed.

[4] H. Zhang *et al.*, "Sliding mode control for ground vehicles: A survey," *Annual Reviews in Control*, 2023, to appear, DOI to be confirmed.

[5] Y. Wang, L. Li, H. Zhang, and W. Chen, "Adaptive sliding mode control for autonomous vehicle lateral motion under uncertain tire-road friction," *IEEE Transactions on Vehicular Technology*, vol. 74, no. 3, pp. 4721–4734, 2025.

[6] Z. Li, P. Chen, G. Wang, and L. Zhao, "Finite-time terminal sliding mode control for autonomous vehicle path tracking with actuator saturation," *Mechanism and Machine Theory*, vol. 194, p. 105568, 2024.

[7] D. Cao, X. Wang, L. Li, C. Lv, X. Na, Y. Xing, X. Li, Y. Li, Y. Chen, and F.-Y. Wang, "Future directions of intelligent vehicles: Potentials, possibilities, and perspectives," *IEEE Transactions on Intelligent Vehicles*, vol. 7, no. 1, pp. 7–10, 2022.

[8] Z. Hu, S. Lou, Y. Xing, X. Wang, D. Cao, and C. Lv, "Review and perspectives on driver digital twin and its enabling technologies for intelligent vehicles," *IEEE Transactions on Intelligent Vehicles*, vol. 7, no. 3, pp. 417–440, 2022.

[9] K. Shi, L. Huang, D. Jiang, Y. Sun, X. Tong, Y. Xie, and Z. Fang, "Path planning optimization of intelligent vehicle based on improved genetic and ant colony hybrid algorithm," *Frontiers in Bioengineering and Biotechnology*, vol. 10, p. 905983, 2022.

[10] E. Sokolovskij and V. Žuraulis, "Advances in vehicle dynamics and road safety: Technologies, simulations, and applications," p. 3735, 2024.

[11] X. Zhang, X. Yu, X. Ye, and S. Pirouzi, "Economic energy managementof networked flexi-renewable energy hubs according to uncertainty modeling by the unscented transformation method," *Energy*, vol. 278, p. 128054, 2023.

[12] A. Altan and R. Hacıoğlu, "Model predictive control of three-axis gimbal system mounted on uav for real-time target tracking under external disturbances," *Mechanical Systems and Signal Processing*, vol. 138, p. 106548, 2020.

[13] C. Johnson, "Accomodation of external disturbances in linear regulator and servomechanism problems," *IEEE Transactions on automatic control*, vol. 16, no. 6, pp. 635–644, 2003.

[14] J. Zhou, Y. Wang, X. Zheng, Z. Wang, and H. Shen, "Weighted h_∞ consensus design for stochastic multi-agent systems subject to external disturbances and adt switching topologies." *Nonlinear Dynamics*, vol. 96, no. 2, p. 853, 2019.

[15] C. Xia, N. Liu, Z. Zhou, Y. Yan, and T. Shi, "Steady-state performance improvement for lqr-based pmsm drives," *IEEE Transactions on Power Electronics*, vol. 33, no. 12, pp. 10 622–10 632, 2018.

[16] J. Liu, Z. Huang, X. Xu, X. Zhang, S. Sun, and D. Li, "Multi-kernel online reinforcement learning for path tracking control of intelligent vehicles," *IEEE Transactions on Systems, Man, and Cybernetics: Systems*, vol. 51, no. 11, pp. 6962–6975, 2020.

[17] L. Guan, L. Guo, X. Wu, L. Xu, and X. Wang, "Path tracking control of hierarchical and refined active disturbance rejection for intelligent vehicles under heterogeneous disturbances," *IEEE Transactions on Vehicular Technology*, 2026.

[18] Z. Zhu, Q. Yao, and Y. Shi, "Intelligent vehicle adaptive robust path tracking control strategy based on mpc and adrc," *International Journal of Automotive Technology*, vol. 27, no. 2, pp. 651–670, 2026.

[19] J. Ji, A. Khajepour, W. W. Melek, and Y. Huang, "Path planning and tracking for vehicle collision avoidance based on model predictive control with multiconstraints," *IEEE transactions on vehicular technology*, vol. 66, no. 2, pp. 952–964, 2016.

[20] C. Sun, X. Zhang, Q. Zhou, and Y. Tian, "A model predictive controller with switched tracking error for autonomous vehicle path tracking," *IEEE access*, vol. 7, pp. 53 103–53 114, 2019.

[21] T. P. Nascimento, C. E. Dórea, and L. M. G. Gonçalves, "Nonholonomic mobile robots' trajectory tracking model predictive control: a survey," *Robotica*, vol. 36, no. 5, pp. 676–696, 2018.

[22] E. Kim, J. Kim, and M. Sunwoo, "Model predictive control strategy for smooth path tracking of autonomous vehicles with steering actuator dynamics," *International Journal of Automotive Technology*, vol. 15, no. 7, pp. 1155–1164, 2014.

[23] Z. Wang, K. Sun, S. Ma, L. Sun, W. Gao, and Z. Dong, "Improved linear quadratic regulator lateral path tracking approach based on a real-time updated algorithm with fuzzy control and cosine similarity for autonomous vehicles," *Electronics*, vol. 11, no. 22, p. 3703, 2022.

[24] H.-j. Zhang, J.-w. Gong, Y. Jiang, G.-m. Xiong, and H.-y. Chen, "An iterative linear quadratic regulator based trajectory tracking controller for wheeled mobile robot," *Journal of Zhejiang University SCIENCE C*, vol. 13, no. 8, pp. 593–600, 2012.

[25] Z. Ge, Y. Wang, and M. Lv, "Three-dimensional trajectory tracking guidance law based on linear quadratic regulator," in *Journal of Physics: Conference Series*, vol. 1039, no. 1. IOP Publishing, 2018, p. 012042.

[26] B. Sun, K. Cui, P. Wang, M. Xie, S. Gao, and S. Cheng, "A lateral and longitudinal control method based on linear quadratic regulator for intelligent vehicles considering future path changes," *Measurement Science and Technology*, vol. 36, no. 1, p. 015103, 2025.

[27] Y. Jiang and T. D. Palaoag, "Trajectory tracking algorithm based on model predictive control and linear quadratic regulator," in *2024 5th International Conference on Artificial Intelligence and Electromechanical Automation (AIEA)*. IEEE, 2024, pp. 1113–1116.

[28] Y. Shtessel, C. Edwards, L. Fridman, A. Levant *et al.*, *Sliding mode control and observation*. Springer, 2014, vol. 10.

[29] H. Feng, Q. Song, S. Ma, W. Ma, C. Yin, D. Cao, and H. Yu, "A new adaptive sliding mode controller based on the rbf neural network for an electro-hydraulic servo system," *ISA Transactions*, vol. 129, pp. 472–484, 2022.

[30] D. Yao, H. Li, and Y. Shi, "Event-based average consensus of disturbed mass via fully distributed sliding mode control," *IEEE Transactions on Automatic Control*, vol. 69, no. 3, pp. 2015–2022, 2023.

[31] J. Ansari, M. Homayounzade, and A. R. Abbasi, "Load frequency control in power systems by a robust backstepping sliding mode controller design," *Energy Reports*, vol. 10, pp. 1287–1298, 2023.

[32] D. Zhang, J. Hu, J. Cheng, Z.-G. Wu, and H. Yan, "A novel disturbance observer based fixed-time sliding mode control for robotic manipulators with global fast convergence," *IEEE/CAA Journal of Automatica Sinica*, vol. 11, no. 3, pp. 661–672, 2024.

[33] J. Tan, K. Zhang, B. Li, and A.-G. Wu, "Event-triggered sliding mode control for spacecraft reorientation with multiple attitude constraints," *IEEE Transactions on Aerospace and Electronic Systems*, vol. 59, no. 5, pp. 6031–6043, 2023.

Response to Reviewer No. 3:

Ms. No. PONE-D-26-32626

"Composite Learning Based Finite Time Nonsingular Terminal Sliding Mode Path Tracking Control for Intelligent Vehicles"

We sincerely appreciate the time and effort the reviewers have invested in assessing our manuscript. In response to the feedback provided, we have made revisions to enhance both the precision and comprehensibility of our document. To facilitate the reviewers' assessment of these changes, we have marked the sections modified in response to **Reviewer 3's comments** in **Purple**. We are grateful for the valuable insights that have significantly improved our manuscript.

Comment: *"Dear Researchers, Thanks for the valuable manuscript. There are some points that need to be addressed to improve the quality. Please find my comments as follows: "*

Response:
We are profoundly grateful to the reviewer for dedicating valuable time and expertise to reviewing our manuscript. The reviewer's thoughtful and constructive feedback has been exceptionally helpful in strengthening the rigor and clarity of this work. We have carefully addressed each comment and believe the revised version now better reflects the contributions and practical significance of the proposed method. Detailed responses are provided below.

1. **Comment 1:** *"The theoretical contributions should be stressed in detail in the Introduction."*

   Response:
   We fully concur with this important suggestion. The Introduction of the original submission presented the theoretical contributions in a relatively concise manner, which may not have adequately conveyed the depth and novelty of the proposed framework. In the revised manuscript, we have substantially expanded the theoretical exposition in three key areas.

   Revision 1: Expanded Comparison of Composite Learning vs. Conventional Adaptive Control

   Original Text (Introduction):

   > Conventional adaptive controllers rely on the persistent excitation (PE) condition for parameter convergence, which is seldom satisfied in real driving scenarios. Consequently, tracking-error-driven controllers such as AFNTSMC fail to ensure parameter convergence [2], degrading model fidelity and tracking accuracy. The composite learning mechanism overcomes this by achieving reliable estimation under interval excitation—a weaker and more practical condition than PE.

Revised Text (Introduction):

> Conventional adaptive controllers rely on the persistent excitation (PE) condition for parameter convergence, which is seldom satisfied in real driving scenarios. PE demands that the regressor vector be persistently exciting over the entire time horizon—a requirement fundamentally incompatible with typical driving patterns. On highways, vehicles spend extended periods in near-straight-line motion with constant longitudinal velocity, rendering the regressor persistently low-rank. In urban environments, repeated stop-and-go patterns similarly fail to provide sustained excitation. Consequently, tracking-error-driven controllers such as AFNTSMC fail to ensure parameter convergence [2], degrading model fidelity and tracking accuracy. The composite learning mechanism overcomes this by incorporating prediction errors from a serial-parallel estimation model (SPEM) to drive the adaptation law. This approach guarantees parameter convergence under interval excitation (IE)—a condition strictly weaker than PE—which requires only that the regressor be excited over any finite time interval, a condition satisfied in most practical driving scenarios. This theoretical insight is central to our contribution.

Revision 2: Strengthened Articulation of the Composite Learning Mechanism and Its Synergistic Integration

Original Text (Contribution 3):

> Non-singular terminal sliding surface is designed to have a nonlinear function of continuously differentiable, which ensures finite time convergence while inherently avoiding singularity. The proposed framework cooperatively integrates compound learning, finite time sliding mode and interference observation, and is supported by strict Lyapunov-based stability analysis, which ensures that tracking error, parameter estimation and interference estimation converge simultaneously.

Revised Text (Contribution 3) – Enhanced with Detailed Theoretical Explanation:

> A nonsingular terminal sliding surface, constructed with a continuously differentiable nonlinear function, is designed to achieve finite-time convergence of the tracking errors while inherently avoiding the singularity problem that plagues conventional terminal sliding mode control. The nonlinear function is piecewise-defined with a linear region near the origin, ensuring C^1 continuity and smooth control signal generation. The proposed framework synergistically integrates three complementary components: (i) **composite learning** that ensures accurate parameter convergence under the significantly relaxed interval excitation condition by leveraging both tracking errors and SPEM-generated prediction errors; (ii) **finite-time nonsingular terminal sliding mode control** that provides rapid convergence and strong robustness against matched

uncertainties without singularity; and (iii) **nonlinear disturbance observer** that provides real-time estimation and feedforward compensation of lumped disturbances. Rigorous Lyapunov-based stability analysis establishes the practical finite-time stability of the closed-loop system, guaranteeing simultaneous convergence of tracking errors, parameter estimation errors, and disturbance estimation errors. This triple convergence property distinguishes our framework from existing methods that typically guarantee only boundedness of tracking errors.

Revision 3: Enhanced Contextual Discussion Linking Theoretical Contributions to Practical Implications

New Paragraph Added Immediately After Contribution Statement:

The theoretical significance of our composite learning approach warrants further elaboration. In conventional adaptive sliding mode control, the parameter adaptation law is driven solely by the tracking error (or sliding variable). Parameter convergence to true values requires the regressor to satisfy the PE condition—a stringent requirement that is mathematically sufficient but practically prohibitive for autonomous driving applications. The composite learning paradigm fundamentally differs by constructing a serial-parallel estimation model that generates a prediction error—the discrepancy between the actual system state and its online estimate. This prediction error, when incorporated into the adaptation law alongside the tracking error, creates a dual-error-driven mechanism. The theoretical consequence, rigorously established in our stability analysis, is that parameter convergence is guaranteed under the interval excitation condition, which merely requires the regressor to be sufficiently rich over any finite interval rather than over the infinite horizon. This relaxation is practically significant: during highway driving, even brief maneuvers such as lane changes or curve negotiation provide sufficient excitation for parameter convergence, eliminating the need for persistent excitation that is never satisfied in steady-state cruising.

Complete Revised Introduction with All Enhancements

Below is the full Introduction section as it now appears in the revised manuscript, with all changes incorporated and color-coded (purple for the revised contribution section, red-Text for other modifications). For brevity, we only show the final revised version of the Introduction in its entirety.

Introduction (Final Revised)

[The opening paragraphs remain unchanged from the original submission.]

Intelligent vehicles have emerged as a transformative technology within modern transportation systems, offering substantial potential to enhance traffic safety, operational efficiency, and environmental sustainability [7, 8, 9]. As a cornerstone enabling technology for autonomous driving, path tracking control (PTC) is instrumental in governing a vehicle's ability to precisely adhere to a prescribed reference trajectory while maintaining dynamic stability [10]. The accuracy of PTC critically influences driving safety and ride comfort, especially in complex and rapidly changing road environments. Despite considerable research efforts, achieving high-performance path tracking for intelligent vehicles remains a persistent challenge, primarily owing to the intrinsic complexity of vehicle dynamics—which are strongly coupled, inherently nonlinear, and subject to a broad spectrum of uncertainties [11].

The challenges in PTC primarily arise from multiple sources of uncertainties, including parametric perturbations, unmodeled dynamics, and external disturbances[12, 13, 14]. These uncertainties can significantly degrade tracking accuracy and even compromise vehicle stability, necessitating the development of robust control strategies capable of handling such complexities while maintaining satisfactory transient and steady-state performance[15]. Various control methodologies have been proposed to address the path tracking problem of intelligent vehicles[16, 17, 18]. Model predictive control (MPC) has gained substantial popularity due to its ability to handle constraints and predict future system behavior[19]. In [20], Sun et al. tackle the issue of insufficient adaptability of conventional MPC to varying driving scenarios in autonomous vehicle path tracking. In [21], Nascimento et al. address the challenges of MPC design for nonholonomic mobile robot trajectory tracking by providing a comprehensive survey that covers modeling, constraint handling, stability analysis, and computational considerations. However, MPC typically relies on accurate system models and may suffer from substantial computational burden, limiting its applicability in real-time implementations[22]. Linear quadratic regulator (LQR) and PID based methods are simple[23, 24, 25]. In [26], Sun et al. address the challenge of coordinating lateral and longitudinal control for intelligent vehicles in the presence of varying road curvatures and future path changes by proposing a LQR based control method that incorporates preview information to optimize tracking performance. Jiang and Palaoag address the trajectory tracking problem for autonomous systems by proposing a hybrid algorithm that integrates MPC with LQR, leveraging MPC's constraint handling capability and LQR's optimality for enhanced tracking performance[27]. However, they often lack robustness to significant uncertainties and disturbances.

Recent years have witnessed a surge of interest in sliding mode control (SMC) for vehicle path tracking, as evidenced by several comprehensive survey papers [3, 4]. These reviews systematically categorize various SMC methodologies—including terminal sliding mode, adaptive sliding mode, and disturbance-observer-based sliding mode—and identify their respective merits and limitations when applied to ground vehicles. Notably, [3] highlights that while SMC offers inherent robustness against matched uncertainties, its application to vehicle path tracking is often hindered by chattering, singularity, and the stringent persistent excitation (PE) condition required for adaptive parameter convergence. These challenges motivate the development of integrated solutions that combine nonlinear control techniques with advanced learning mechanisms. SMC has emerged as a powerful robust control technique for nonlinear systems with uncertainties, owing to its insensitivity to matched disturbances and its capability to enforce desired system behavior through discontinuous control actions[28, 29, 30]. In [31], Ansari et al. address the load frequency control problem in power systems subject to parameter uncertainties and external disturbances by proposing a robust backstepping SMC that integrates the systematic design of backstepping with the robustness of sliding mode control to ensure frequency stability. In [11], Zhang et al. address the trajectory tracking control problem of permanent magnet synchronous motors subject to parameter uncertainties, load disturbances, and sliding mode chattering by proposing a non-singular fast terminal SMC scheme with disturbance compensation. In [32], Zhang et al. tackle the challenges of finite-time convergence and disturbance sensitivity in robotic manipulator control. They propose a fixed-time SMC integrated with a disturbance observer, which guarantees global fast tracking convergence with a convergent time upper bound independent of initial states, while effectively estimating and compensating for lumped disturbances. In [33], Tan et al. address the spacecraft reorientation control problem under multiple attitude constraints and actuator resource limitations by proposing an event-triggered sliding mode control scheme, which achieves robust attitude tracking while significantly reducing actuator updates and ensuring the avoidance of Zeno behavior. More recently, several studies have specifically explored SMC for autonomous vehicle applications. In [5], Wang et al. proposes an adaptive sliding mode approach for vehicle lateral control under uncertain tire-road friction conditions. In [6], Li et al. develops a finite-time terminal sliding mode controller for path tracking of autonomous vehicles, demonstrating improved transient performance. However, these methods either require stringent PE conditions for parameter convergence or lack a systematic mechanism for disturbance estimation and compensation, leaving room for further improvement.

Conventional adaptive controllers rely on the persistent excitation (PE) condition for parameter convergence, which is seldom satisfied in real driving scenarios. PE demands that the regressor vector be persistently exciting over the entire time horizon—a requirement fundamentally incompatible with typical driving patterns. On highways, vehicles spend extended periods in near-straight-line motion with constant longitudinal velocity, rendering the regressor persistently low-rank. In urban environments, repeated stop-and-go patterns similarly fail to provide sustained excitation. Consequently, tracking-error-driven controllers such as AFNTSMC fail to ensure parameter convergence[2], degrading model fidelity and tracking accuracy. The composite learning mechanism overcomes this by in-

corporating prediction errors from a serial-parallel estimation model (SPEM) to drive the adaptation law. This approach guarantees parameter convergence under interval excitation (IE)—a condition strictly weaker than PE—which requires only that the regressor be excited over any finite time interval, a condition satisfied in most practical driving scenarios. This theoretical insight is central to our contribution.

Motivated by the aforementioned discussions, this paper proposes a composite learning-based finite-time nonsingular terminal sliding mode control (CL-FNTSMC) strategy for intelligent vehicle path tracking. The main contributions are threefold:

0.1 A unified path tracking error model is established that explicitly accounts for lumped uncertainties encompassing parametric variations, unmodeled dynamics, and external disturbances. A nonlinear disturbance observer is incorporated to provide real-time estimation and feedforward compensation, effectively reducing the conservativeness of robust control design.

0.2 A composite learning law is developed that simultaneously exploits tracking errors and prediction errors from a serial-parallel estimation model, enabling accurate online parameter estimation without the restrictive PE condition. Theoretical analysis establishes parameter convergence under interval excitation, which is strictly weaker than PE and practically more relevant.

0.3 A nonsingular terminal sliding surface, constructed with a continuously differentiable nonlinear function, is designed to achieve finite-time convergence of the tracking errors while inherently avoiding the singularity problem that plagues conventional terminal sliding mode control. The nonlinear function is piecewise-defined with a linear region near the origin, ensuring C^1 continuity and smooth control signal generation. The proposed framework synergistically integrates three complementary components: (i) **composite learning** that ensures accurate parameter convergence under the significantly relaxed interval excitation condition by leveraging both tracking errors and SPEM-generated prediction errors; (ii) **finite-time nonsingular terminal sliding mode control** that provides rapid convergence and strong robustness against matched uncertainties without singularity; and (iii) **nonlinear disturbance observer** that provides real-time estimation and feedforward compensation of lumped disturbances. Rigorous Lyapunov-based stability analysis establishes the practical finite-time stability of the closed-loop system, guaranteeing simultaneous convergence of tracking errors, parameter estimation errors, and disturbance estimation errors. This triple convergence property distinguishes our framework from existing methods that typically guarantee only boundedness of tracking errors.

The theoretical significance of our composite learning approach warrants further elaboration. In conventional adaptive sliding mode control, the parameter adaptation law is driven solely by the tracking error (or sliding variable). Parameter convergence to true values requires the regressor to satisfy the PE condition—a stringent requirement that is mathematically sufficient but practically prohibitive for autonomous driving applications. The composite learning paradigm fundamentally differs by constructing a serial-parallel estimation model that generates a prediction error—the discrepancy between the actual

system state and its online estimate. This prediction error, when incorporated into the adaptation law alongside the tracking error, creates a dual-error-driven mechanism. The theoretical consequence, rigorously established in our stability analysis, is that parameter convergence is guaranteed under the interval excitation condition, which merely requires the regressor to be sufficiently rich over any finite interval rather than over the infinite horizon. This relaxation is practically significant: during highway driving, even brief maneuvers such as lane changes or curve negotiation provide sufficient excitation for parameter convergence, eliminating the need for persistent excitation that is never satisfied in steady-state cruising.

The remainder of this paper is organized as follows. Section 2 presents the problem formulation and the vehicle path tracking model. Section 3 details the design of the proposed CL-FNTSMC strategy, including the composite learning laws, the nonsingular terminal sliding surface, the disturbance observer, and the stability analysis. Section 4 presents simulation results and comparative studies. Finally, Section 5 concludes the paper with discussions and future research directions.

Summary of Revisions

Section	Original	Revised
PE vs. IE comparison	Brief mention	Expanded with practical examples (highway, urban) and formal theoretical distinction
Composite learning mechanism	Concise statement	Detailed explanation of SPEM, prediction error, dual-error-driven adaptation
Contribution 3	Brief list of features	Detailed exposition with bullet points on each component's role and their synergy
New theoretical paragraph	Absent	Dedicated paragraph on theoretical significance of composite learning
Triple convergence property	Implicit	Explicitly stated and distinguished from existing methods

Table 9: Overview of revisions made to the Introduction

Conventional adaptive controllers rely on the persistent excitation (PE) condition for parameter convergence, which is seldom satisfied in real driving scenarios. Consequently, tracking-error-driven controllers such as AFNTSMC fail to ensure parameter convergence[2], degrading model fidelity and tracking accuracy. The composite learning mechanism overcomes this by achieving reliable estimation under interval excitation—a weaker and more practical condition than PE. Motivated by the aforementioned discussions, this paper proposes a composite learning-based finite-time nonsingular terminal sliding mode control (CL-FNTSMC) strategy for intelligent vehicle path tracking. The main contributions are threefold:

0.1 A unified path tracking error model is established that explicitly accounts for lumped uncertainties encompassing parametric variations, unmodeled dynamics, and external disturbances. A nonlinear disturbance observer is incorporated to provide real-time estimation and feedforward compensation, effectively reducing the conservativeness of robust control design.

0.2 A composite learning law is developed that simultaneously exploits tracking errors and prediction errors from a serial-parallel estimation model, enabling accurate online parameter estimation without the restrictive PE condition. Theoretical analysis establishes parameter convergence under interval excitation, which is strictly weaker than PE and practically more relevant.

0.3 Non-singular terminal sliding surface is designed to have a nonlinear function of continuously differentiable, which ensures finite time convergence while inherently avoiding singularity. The proposed framework cooperatively integrates compound learning, finite time sliding mode and interference observation, and is supported by strict Lyapunov-based stability analysis, which ensures that tracking error, parameter

estimation and interference estimation converge simultaneously. A large number of comparative simulations in different scenarios confirm that the proposed method is obviously superior to PID, traditional NTSMC and the most advanced AFNTSMC.

The remainder of this paper is organized as follows. Section 2 presents the problem formulation and the vehicle path tracking model. Section 3 details the design of the proposed CL-FNTSMC strategy, including the composite learning laws, the nonsingular terminal sliding surface, the disturbance observer, and the stability analysis. Section 4 presents simulation results and comparative studies. Finally, Section 5 concludes the paper with discussions and future research directions.

......

We trust that these comprehensive revisions satisfactorily address the editor's request to strengthen the theoretical contributions in the Introduction. All modifications have been carefully reviewed to ensure consistency with the remainder of the manuscript while enhancing the clarity and impact of our theoretical contributions.

2. **Comment 2:** *"Please check all notations and equations carefully. Moreover, the use of English should be improved."*

Response:

We sincerely thank the reviewer for this valuable suggestion. We fully agree that careful verification of mathematical notations and equations, as well as thorough language polishing, are essential for ensuring the clarity and quality of the manuscript. We have therefore undertaken a comprehensive revision of the entire manuscript, with particular attention to the reviewer's concerns.

Modification

......

Vehicle Dynamic Model

To incorporate the essential inertial and tire-force characteristics, we adopt the widely used single-track (bicycle) dynamic model. The vehicle is assumed to have front-wheel steering only, and the state variables are defined as the longitudinal velocity v_x, lateral velocity v_y, and yaw rate ω in the body frame. The equations of motion are:

$$\dot{v}_x = \frac{1}{m}\left(F_{xf}\cos\delta_f - F_{yf}\sin\delta_f + F_{xr}\right) + v_y\omega, \tag{36a}$$

$$\dot{v}_y = \frac{1}{m}\left(F_{xf}\sin\delta_f + F_{yf}\cos\delta_f + F_{yr}\right) - v_x\omega, \tag{36b}$$

$$\dot{\omega} = \frac{1}{I_z}\left(l_f F_{yf}\cos\delta_f - l_r F_{yr} + l_f F_{xf}\sin\delta_f\right), \tag{36c}$$

where m is the vehicle mass, I_z is the yaw moment of inertia, and l_f and l_r are the distances from the centre of gravity (CG) to the front and rear axles, respectively; δ_f is the front steering angle, which serves as the control input; F_{xf} and F_{xr} are the longitudinal tire forces at the front and rear; and F_{yf} and F_{yr} are the lateral tire forces.

For moderate lateral accelerations, the lateral tire forces can be approximated by a linear relationship with the corresponding slip angles:

$$F_{yf} = C_f \alpha_f, \qquad F_{yr} = C_r \alpha_r, \tag{37}$$

where C_f and C_r are the cornering stiffnesses (positive constants), and α_f and α_r are the front and rear slip angles, respectively, given by:

$$\alpha_f = \delta_f - \frac{v_y + l_f \omega}{v_x}, \qquad \alpha_r = \frac{l_r \omega - v_y}{v_x}. \tag{38}$$

To facilitate control design, we assume that the longitudinal speed is maintained at a nearly constant value $v_x \approx v$ by a separate controller. Under this assumption, the longitudinal dynamics can be neglected. Furthermore, we ignore the longitudinal tire forces ($F_{xf} = F_{xr} = 0$), as the steering control primarily affects the lateral motion. Substituting the linear tire force expressions and linearising about a straight-line motion with small steering angles, we obtain the following lateral-yaw dynamics:

$$\dot{v}_y = -\frac{C_f + C_r}{mv} v_y + \left(\frac{C_f + C_r}{m} - v \right) \omega + \frac{C_f}{m} \delta_f, \tag{39a}$$

$$\dot{\omega} = -\frac{l_f C_f - l_r C_r}{I_z v} v_y - \frac{l_f^2 C_f + l_r^2 C_r}{I_z v} \omega + \frac{l_f C_f}{I_z} \delta_f. \tag{39b}$$

In the above derivation, we have used the small-angle approximations $\cos \delta_f \approx 1$ and $\sin \delta_f \approx \delta_f$, together with the slip-angle expressions $\alpha_f \approx \delta_f - (v_y + l_f \omega)/v$ and $\alpha_r \approx (l_r \omega - v_y)/v$. The term v appearing in the coefficient of ω arises from the centripetal acceleration term $v\omega$ after linearisation.

Remark 2: The linear tire model is valid only for small slip angles. Under higher lateral accelerations, nonlinear tire characteristics become significant and should be considered. Nevertheless, for the purpose of control design, the linear model provides a reasonable approximation. The unmodeled nonlinearities and parameter variations are treated as lumped disturbances, which will be compensated by the proposed adaptive neural network controller in the following section.

......

0.2 Path Tracking Error Dynamics

To facilitate the controller design for path tracking, we express the vehicle dynamics in terms of the path tracking errors. Under the assumptions of small yaw error e_ψ and constant longitudinal speed v, the lateral error e_y and yaw error e_ψ are related to the vehicle states v_y and ω by the following kinematic relations:

$$\dot{e}_y = v e_\psi + v_y, \qquad \dot{e}_\psi = \omega - \omega_d, \tag{40}$$

where ω_d denotes the desired yaw rate corresponding to the reference path curvature.

Taking the time derivatives of the above equations and substituting the dynamic equations from Eq. (39), we obtain the second-order error dynamics:

$$\ddot{e}_y = -\frac{C_f + C_r}{mv}\dot{e}_y + \frac{C_f + C_r}{m}e_\psi - \frac{l_f C_f - l_r C_r}{mv}\dot{e}_\psi + \frac{C_f}{m}\delta_f + \Delta_y, \tag{41a}$$

$$\ddot{e}_\psi = -\frac{l_f C_f - l_r C_r}{I_z v}\dot{e}_y - \frac{l_f C_f - l_r C_r}{I_z}e_\psi - \frac{l_f^2 C_f + l_r^2 C_r}{I_z v}\dot{e}_\psi + \frac{l_f C_f}{I_z}\delta_f + \Delta_\psi, \tag{41b}$$

where the lumped disturbances Δ_y and Δ_ψ collectively account for the following: parametric uncertainties in vehicle parameters (m, I_z, C_f, C_r, l_f, l_r), unmodeled dynamics (e.g., nonlinear tire characteristics and suspension effects), external disturbances (e.g., wind gusts, road bank angle, and measurement noise), and residual coupling errors arising from the linearisation process. All lumped disturbance terms are assumed to be bounded and slowly time-varying relative to the system dynamics.

Assumption 1: The lumped disturbances Δ_y and Δ_ψ, along with their time derivatives, are bounded. That is, there exist positive constants $\bar{\Delta}$ and $\bar{\Delta}_1$ such that $\|\boldsymbol{\Delta}(t)\| \leq \bar{\Delta}$ and $\|\dot{\boldsymbol{\Delta}}(t)\| \leq \bar{\Delta}_1$ for all $t \geq 0$, where $\boldsymbol{\Delta} = [\Delta_y, \Delta_\psi]^{\mathrm{T}}$.

Assumption 2: The reference path is smooth, and its derivatives up to second order are bounded. In particular, ω_d and $\dot{\omega}_d$ are bounded.

0.3 State-Space Representation

Define the state vector $\boldsymbol{x} = [x_1, x_2, x_3, x_4]^{\mathrm{T}} \in \mathbb{R}^4$ as:

$$x_1 = e_y, \quad x_2 = \dot{e}_y, \quad x_3 = e_\psi, \quad x_4 = \dot{e}_\psi. \tag{42}$$

Then, the error dynamics in Eqs. (41a)–(41b) can be written in the following compact state-space form:

$$\dot{\boldsymbol{x}} = \boldsymbol{A}\boldsymbol{x} + \boldsymbol{B}u + \boldsymbol{D}(\boldsymbol{x}, t), \tag{43}$$

where $u = \delta_f$ is the control input (front steering angle), and the system matrix $\boldsymbol{A} \in \mathbb{R}^{4\times 4}$ and input matrix $\boldsymbol{B} \in \mathbb{R}^4$ are given by:

$$\boldsymbol{A} = \begin{bmatrix} 0 & 1 & 0 & 0 \\ 0 & -\dfrac{C_f + C_r}{mv} & \dfrac{C_f + C_r}{m} & -\dfrac{l_f C_f - l_r C_r}{mv} \\ 0 & 0 & 0 & 1 \\ 0 & -\dfrac{l_f C_f - l_r C_r}{I_z v} & -\dfrac{l_f C_f - l_r C_r}{I_z} & -\dfrac{l_f^2 C_f + l_r^2 C_r}{I_z v} \end{bmatrix}, \tag{44}$$

$$\boldsymbol{B} = \begin{bmatrix} 0 \\ \dfrac{C_f}{m} \\ 0 \\ \dfrac{l_f C_f}{I_z} \end{bmatrix}. \tag{45}$$

The lumped disturbance vector $\boldsymbol{D}(\boldsymbol{x}, t)$ is defined as:

$$\boldsymbol{D}(\boldsymbol{x}, t) = \left[0,\ \Delta_y(t),\ 0,\ \Delta_\psi(t)\right]^{\mathrm{T}}. \tag{46}$$

For control design, we use a nominal model based on the nominal parameter values (denoted with the subscript 0), while the actual plant parameters may deviate from these nominal values. We define the parametric uncertainties as $\Delta\boldsymbol{A} = \boldsymbol{A}_{\text{act}} - \boldsymbol{A}_{\text{nom}}$ and $\Delta\boldsymbol{B} = \boldsymbol{B}_{\text{act}} - \boldsymbol{B}_{\text{nom}}$, and incorporate these into the lumped disturbance term. Consequently, the state equation can be rewritten as:

$$\dot{\boldsymbol{x}} = \boldsymbol{A}_{\text{nom}}\boldsymbol{x} + \boldsymbol{B}_{\text{nom}}u + \boldsymbol{D}_{\text{tot}}(\boldsymbol{x}, t), \tag{47}$$

where $\boldsymbol{D}_{\text{tot}}(\boldsymbol{x}, t) = \Delta\boldsymbol{A}\boldsymbol{x} + \Delta\boldsymbol{B}u + \boldsymbol{D}(\boldsymbol{x}, t)$ contains all uncertainties.

Remark 3: The model in Eq. (43) is linear time-invariant except for the disturbance term. The controller design in the following section uses only the nominal matrices $\boldsymbol{A}_{\text{nom}}$ and $\boldsymbol{B}_{\text{nom}}$. The effects of uncertainties and disturbances are handled by the composite learning law and the nonlinear disturbance observer.

0.4 Problem Statement

Given the state-space model in Eq. (43) with unknown but bounded lumped disturbances $\boldsymbol{D}_{\text{tot}}(\boldsymbol{x}, t)$, and given a smooth reference path with bounded curvature, the control objective is to design a steering control law u such that the following requirements are satisfied:

0.1 The tracking error vector $\boldsymbol{x}(t)$ converges to a small neighbourhood of the origin in finite time. That is, for a prescribed small constant $\varepsilon > 0$, there exists a finite settling time $T_s < \infty$ such that $\|\boldsymbol{x}(t)\| \leq \varepsilon$ for all $t \geq T_s$;

0.2 The closed-loop system is robust against parametric uncertainties and external disturbances;

0.3 The control input u is smooth and free of chattering, while the singularity problem inherent in conventional terminal sliding mode control is effectively avoided.

Remark 4: The finite-time convergence property is particularly important for autonomous driving applications, as it guarantees that the vehicle can quickly recover from initial tracking errors or sudden disturbances, thereby enhancing overall driving safety.

We appreciate the reviewer's expertise and thoughtful guidance throughout this process. The revised manuscript has been greatly improved as a result of this constructive review.

3. **Comment 3:** *"The advantages of the proposed algorithm over well-known algorithms should be stressed. I suggest mentioning/comparing the simulations with the results of the recent valid references."*

   Response:
   We fully agree with your assessment. To clearly demonstrate the novelty and superiority of our proposed Composite Learning-based Finite-Time Nonsingular Terminal Sliding Mode Control (CL-FNTSMC), we have made the following revisions:

......

Intelligent vehicles have emerged as a transformative technology within modern transportation systems, offering substantial potential to enhance traffic safety, operational efficiency, and environmental sustainability [7, 8, 9]. As a cornerstone enabling technology for autonomous driving, path tracking control (PTC) is instrumental in governing a vehicle's ability to precisely adhere to a prescribed reference trajectory while maintaining dynamic stability [10]. The accuracy of PTC critically influences driving safety and ride comfort, especially in complex and rapidly changing road environments. Despite considerable research efforts, achieving high-performance path tracking for intelligent vehicles remains a persistent challenge, primarily owing to the intrinsic complexity of vehicle dynamics—which are strongly coupled, inherently nonlinear, and subject to a broad spectrum of uncertainties [11].

The challenges in PTC primarily arise from multiple sources of uncertainties, including parametric perturbations, unmodeled dynamics, and external disturbances[12, 13, 14]. These uncertainties can significantly degrade tracking accuracy and even compromise vehicle stability, necessitating the development of robust control strategies capable of handling such complexities while maintaining satisfactory transient and steady-state performance[15]. Various control methodologies have been proposed to address the path tracking problem of intelligent vehicles[16, 17, 18]. Model predictive control (MPC) has gained substantial popularity due to its ability to handle constraints and predict future system behavior[19]. In [20], Sun et al. tackle the issue of insufficient adaptability of conventional MPC to varying driving scenarios in autonomous vehicle path tracking. In [21], Nascimento et al. address the challenges of MPC design for nonholonomic mobile robot trajectory tracking by providing a comprehensive survey that covers modeling, constraint handling, stability analysis, and computational considerations. However, MPC typically relies on accurate system models and may suffer from substantial computational burden, limiting its applicability in real-time implementations[22]. Linear quadratic regulator (LQR) and PID based methods are simple[23, 24, 25]. In [26], Sun et al. address the challenge of coordinating lateral and longitudinal control for intelligent vehicles in the presence of varying road curvatures and future path changes by proposing a LQR based control method that incorporates preview information to optimize tracking performance. Jiang and Palaoag address the trajectory tracking problem for autonomous systems by proposing a hybrid algorithm that integrates MPC with LQR, leveraging MPC's constraint handling capability and LQR's optimality for enhanced tracking performance[27]. However, they often lack robustness to significant uncertainties and disturbances.

Recent years have witnessed a surge of interest in sliding mode control (SMC) for vehicle path tracking, as evidenced by several comprehensive survey papers [3, 4]. These reviews systematically categorize various SMC methodologies—including terminal sliding mode, adaptive sliding mode, and disturbance-observer-based sliding mode—and identify their respective merits and limitations when applied to ground vehicles. Notably, [3] highlights that while SMC offers inherent robustness against matched uncertainties, its application to vehicle path tracking is often hindered by chattering, singularity, and the stringent persistent excitation (PE) condition required for adaptive parameter convergence. These challenges motivate the development of integrated solutions that combine nonlinear control

techniques with advanced learning mechanisms. SMC has emerged as a powerful robust control technique for nonlinear systems with uncertainties, owing to its insensitivity to matched disturbances and its capability to enforce desired system behavior through discontinuous control actions[28, 29, 30]. In [31], Ansari et al. address the load frequency control problem in power systems subject to parameter uncertainties and external disturbances by proposing a robust backstepping SMC that integrates the systematic design of backstepping with the robustness of sliding mode control to ensure frequency stability. In [11], Zhang et al. address the trajectory tracking control problem of permanent magnet synchronous motors subject to parameter uncertainties, load disturbances, and sliding mode chattering by proposing a non-singular fast terminal SMC scheme with disturbance compensation. In [32], Zhang et al. tackle the challenges of finite-time convergence and disturbance sensitivity in robotic manipulator control. They propose a fixed-time SMC integrated with a disturbance observer, which guarantees global fast tracking convergence with a convergent time upper bound independent of initial states, while effectively estimating and compensating for lumped disturbances. In [33], Tan et al. address the spacecraft reorientation control problem under multiple attitude constraints and actuator resource limitations by proposing an event-triggered sliding mode control scheme, which achieves robust attitude tracking while significantly reducing actuator updates and ensuring the avoidance of Zeno behavior. More recently, several studies have specifically explored SMC for autonomous vehicle applications. In [5], Wang et al. proposes an adaptive sliding mode approach for vehicle lateral control under uncertain tire-road friction conditions. In [6], Li et al. develops a finite-time terminal sliding mode controller for path tracking of autonomous vehicles, demonstrating improved transient performance. However, these methods either require stringent PE conditions for parameter convergence or lack a systematic mechanism for disturbance estimation and compensation, leaving room for further improvement.

Conventional adaptive controllers rely on the persistent excitation (PE) condition for parameter convergence, which is seldom satisfied in real driving scenarios. Consequently, tracking-error-driven controllers such as AFNTSMC fail to ensure parameter convergence[2], degrading model fidelity and tracking accuracy. The composite learning mechanism overcomes this by achieving reliable estimation under interval excitation—a weaker and more practical condition than PE. Motivated by the aforementioned discussions, this paper proposes a composite learning-based finite-time nonsingular terminal sliding mode control (CL-FNTSMC) strategy for intelligent vehicle path tracking. The main contributions are threefold:

0.1 A unified path tracking error model is established that explicitly accounts for lumped uncertainties encompassing parametric variations, unmodeled dynamics, and external disturbances. A nonlinear disturbance observer is incorporated to provide real-time estimation and feedforward compensation, effectively reducing the conservativeness of robust control design.

0.2 A composite learning law is developed that simultaneously exploits tracking errors and prediction errors from a serial-parallel estimation model, enabling accurate online parameter estimation without the restrictive PE condition. Theoretical analysis establishes parameter convergence under interval excitation, which is strictly weaker

than PE and practically more relevant.

0.3 Non-singular terminal sliding surface is designed to have a nonlinear function of continuously differentiable, which ensures finite time convergence while inherently avoiding singularity. The proposed framework cooperatively integrates compound learning, finite time sliding mode and interference observation, and is supported by strict Lyapunov-based stability analysis, which ensures that tracking error, parameter estimation and interference estimation converge simultaneously. A large number of comparative simulations in different scenarios confirm that the proposed method is obviously superior to PID, traditional NTSMC and the most advanced AFNTSMC.

The remainder of this paper is organized as follows. Section 2 presents the problem formulation and the vehicle path tracking model. Section 3 details the design of the proposed CL-FNTSMC strategy, including the composite learning laws, the nonsingular terminal sliding surface, the disturbance observer, and the stability analysis. Section 4 presents simulation results and comparative studies. Finally, Section 5 concludes the paper with discussions and future research directions.

We believe that these revisions adequately address your concerns and clearly demonstrate the novelty and superior performance of our proposed CL-FNTSMC framework. We hope that the revised manuscript now meets the high standards of PLOS ONE.

4. **Comment 4:** *"In the introduction section, it is not enough to state the current work. It should be expanded and reconstructed. Including the motivation, the main difficulties, the main work, and the improvements compared with previous related works should be emphasised in this section."*

Response:
We sincerely thank you for your careful and constructive review. Your suggestion to *"expand and reconstruct the introduction section"* and to *"emphasize the motivation, main difficulties, main work, and improvements compared with previous works"* is highly valuable and has significantly improved the clarity and impact of our manuscript.

In the following, we provide a detailed response to your comments. All modifications in the revised manuscript are highlighted in purple for your easy identification.

We systematically identify and elaborate on the key difficulties:

- Multiple sources of uncertainties (parametric perturbations, unmodeled dynamics, external disturbances) and their adverse effects on tracking accuracy and vehicle stability;
- Limitations of existing control methods: MPC suffers from high computational burden and model dependency; LQR/PID methods lack robustness against significant uncertainties and disturbances;
- Limitations of recent sliding mode control (SMC) approaches: chattering, singularity, and the stringent **persistent excitation (PE)** condition required for adaptive parameter convergence—a condition rarely satisfied in real-world driving scenarios.

We thank you once again for your valuable suggestions, which have greatly improved the quality of this manuscript.

5. **Comment 5:** *"The importance of the problem considered in this paper should be further addresse."*

   Response:

   We thank the reviewer for this valuable suggestion. In the revised manuscript, we have **significantly strengthened the statement of problem importance** in the introduction section through the following specific enhancements:

   0.1 **Expanded opening paragraph:** We now emphasize not only the technical significance but also the societal impact of path tracking control (PTC) for intelligent vehicles. Specifically, we highlight:

   - The transformative role of intelligent vehicles in enhancing **traffic safety, operational efficiency, and environmental sustainability** [7, 8, 9];
   - The critical dependence of **driving safety and ride comfort** on PTC accuracy, especially in complex and rapidly changing environments;
   - The persistent challenge posed by the **intrinsic complexity of vehicle dynamics**—strongly coupled, nonlinear, and subject to broad uncertainties [11].

   These additions clearly establish the practical urgency of developing high-performance PTC solutions.

   0.2 **Enhanced discussion of consequences:** In the second paragraph, we explicitly state that uncertainties "can significantly degrade tracking accuracy and **even compromise vehicle stability**" [15], directly linking the technical problem to the safety-critical nature of autonomous driving.

   0.3 **Emphasis on the practical failure of existing methods:** We added a dedicated paragraph explaining that the stringent **persistent excitation (PE)** condition—required by conventional adaptive controllers—is **seldom satisfied in real driving scenarios** (e.g., highway cruising, urban stop-and-go traffic). This practical limitation is now highlighted as a key motivation, because without reliable parameter convergence, model fidelity degrades and tracking accuracy suffers, which poses a real threat to vehicle safety.

   0.4 **Clear alignment of contributions with importance:** Our three main contributions are now explicitly linked to overcoming these practical difficulties:

   - The composite learning law relaxes the PE condition to **interval excitation**—a condition practically feasible in realistic driving;
   - The nonlinear disturbance observer provides **real-time compensation** of unknown disturbances, ensuring robustness where other methods fail;
   - The non-singular terminal sliding surface guarantees **finite-time convergence** without singularity, which is essential for rapid recovery from unexpected disturbances (e.g., obstacle avoidance, emergency lane changes).

   We are grateful for this suggestion, which has substantially improved the overall motivation and clarity of our manuscript.

6. **Comment 6:** *"The types of software employed for solving the problem and for simulation experiments should be stated clearly."*

Response:
We thank the reviewer for this important suggestion. We fully agree that a clear description of the software environment is essential for reproducibility and transparency.

In the revised manuscript, we have explicitly added the software details in the **Simulation Environment and Parameter Settings** subsection (Section 4.1). Specifically, we now state that:

- All numerical simulations were implemented using **MATLAB R2022b** with the **Simulink** environment.
- The ordinary differential equations (ODEs) were solved using the **ode45** (Dormand–Prince) solver with a fixed time step of $\Delta t = 0.001$ s.
- The vehicle dynamics and the proposed controller were implemented using **embedded MATLAB Function blocks** in Simulink, and no third-party toolboxes beyond the standard MATLAB/Simulink libraries were required.

We have added this information in the revised text (see purple highlight in Section 4.1). This ensures that any researcher can reproduce our simulations using readily available commercial software.

......

Modification

......

To validate the effectiveness of the proposed composite learning-based finite-time non-singular terminal sliding mode control (CL-FNTSMC) strategy, comprehensive numerical simulations are conducted using the MATLAB/Simulink platform. The vehicle dynamics are implemented based on the linearized error model , with the nominal parameters used for controller design and actual parameters incorporating uncertainties to emulate real-world conditions. The simulation is performed with a fixed time step of $\Delta t = 0.001$ s over a total duration of $T = 10$ s.

All simulations were implemented in MATLAB R2022b with Simulink. The system ordinary differential equations (ODEs) were solved using the ode45 (Dormand–Prince) solver with a fixed integration step size of 1 ms. The controller and the nonlinear disturbance observer were coded as embedded MATLAB Function blocks within the Simulink model. No additional third-party toolboxes were required, ensuring that the entire simulation framework can be reproduced using standard MATLAB/Simulink licenses.

We are grateful for this suggestion, which improves the clarity and reproducibility of our work.

7. **Comment 7:** *"The directions to further and improve the work should be expanded by adding a more detailed future recommendation section after the conclusions section."*

   Response:
   We sincerely thank the reviewer for this constructive suggestion. We fully agree that a detailed discussion of future research directions enhances the completeness and impact of the manuscript.

In the revised manuscript, we have significantly **expanded the future work section** at the end of the Conclusions (Section 5). The original brief statement has been replaced with a comprehensive discussion that outlines **four specific research directions**:

0.1 **Extension to integrated longitudinal-lateral control:** We plan to extend the proposed CL-FNTSMC framework to handle simultaneous longitudinal and lateral control (integrated vehicle dynamics), considering the coupling effects between acceleration, braking, and steering. This will enable more comprehensive vehicle motion control in complex driving scenarios such as emergency obstacle avoidance and high-speed cornering.

0.2 **Inclusion of actuator constraints and saturation compensation:** We intend to explicitly incorporate steering actuator constraints (both amplitude and rate limits) into the controller design using constrained control techniques such as barrier Lyapunov functions or anti-windup compensators. This will further enhance the practical applicability of the framework.

0.3 **Hardware-in-the-loop (HIL) experimental validation:** We plan to conduct HIL experiments and real-vehicle tests to validate the proposed algorithm under realistic conditions, including sensor noise, communication delays, and actual steering system dynamics. This will bridge the gap between theoretical development and practical deployment.

0.4 **Integration with decision-making and perception modules:** We aim to integrate the proposed low-level path tracking controller with higher-level decision-making and perception systems (e.g., obstacle detection, path planning), enabling the complete autonomous driving stack to operate in dynamic, cluttered environments.

......

Modification

......

Future work will extend the proposed framework in four directions: (i) integrated longitudinal-lateral control that explicitly accounts for the coupling between acceleration, braking, and steering dynamics; (ii) explicit incorporation of actuator amplitude and rate constraints using barrier Lyapunov functions or anti-windup compensation to enhance practical feasibility; (iii) hardware-in-the-loop and real-vehicle experiments to validate the algorithm under realistic conditions including sensor noise, communication delays, and steering system nonlinearities; and (iv) seamless integration with higher-level perception and decision-making modules to enable complete autonomous driving in dynamic environments.

......

All these additions are clearly marked in purple in the revised manuscript (see Section 5, the last paragraph). We believe these expanded future directions provide a clear roadmap for the continuation of this research and demonstrate the extensibility of our proposed framework.

We are grateful for this suggestion, which has significantly improved the completeness and forward-looking perspective of our manuscript.

The authors sincerely appreciate the reviewer's detailed and insightful feedback, which has significantly contributed to improving both the quality and clarity of our manuscript. We are truly grateful for the time and effort invested in offering constructive criticism and valuable suggestions, helping us enhance both the substance and presentation of our work.

References

[1] L. Wang, J. Zhao, Z. Yu, Z. Pan, and Z. Zheng, "Robust and high-precision position control of pmlsm-driven feed servo system based on adaptive fast nonsingular terminal sliding mode," *IEEE Transactions on Transportation Electrification*, vol. 11, no. 1, pp. 4882–4894, 2024.

[2] J. Liu, Y. Cui, H. Wang, Y. Tian, and X. Zhou, "Rigid spacecraft attitude control based on finite-time non-singular sliding mode and extended state disturbance observer with input hysteresis," *International Journal of Robust and Nonlinear Control*, vol. 35, no. 10, pp. 4187–4200, 2025.

[3] Y. Cheng *et al.*, "Sliding mode control for autonomous vehicle path tracking: A comprehensive review," *IEEE Transactions on Intelligent Vehicles*, 2024, to appear, DOI to be confirmed.

[4] H. Zhang *et al.*, "Sliding mode control for ground vehicles: A survey," *Annual Reviews in Control*, 2023, to appear, DOI to be confirmed.

[5] Y. Wang, L. Li, H. Zhang, and W. Chen, "Adaptive sliding mode control for autonomous vehicle lateral motion under uncertain tire-road friction," *IEEE Transactions on Vehicular Technology*, vol. 74, no. 3, pp. 4721–4734, 2025.

[6] Z. Li, P. Chen, G. Wang, and L. Zhao, "Finite-time terminal sliding mode control for autonomous vehicle path tracking with actuator saturation," *Mechanism and Machine Theory*, vol. 194, p. 105568, 2024.

[7] D. Cao, X. Wang, L. Li, C. Lv, X. Na, Y. Xing, X. Li, Y. Li, Y. Chen, and F.-Y. Wang, "Future directions of intelligent vehicles: Potentials, possibilities, and perspectives," *IEEE Transactions on Intelligent Vehicles*, vol. 7, no. 1, pp. 7–10, 2022.

[8] Z. Hu, S. Lou, Y. Xing, X. Wang, D. Cao, and C. Lv, "Review and perspectives on driver digital twin and its enabling technologies for intelligent vehicles," *IEEE Transactions on Intelligent Vehicles*, vol. 7, no. 3, pp. 417–440, 2022.

[9] K. Shi, L. Huang, D. Jiang, Y. Sun, X. Tong, Y. Xie, and Z. Fang, "Path planning optimization of intelligent vehicle based on improved genetic and ant colony hybrid algorithm," *Frontiers in Bioengineering and Biotechnology*, vol. 10, p. 905983, 2022.

[10] E. Sokolovskij and V. Žuraulis, "Advances in vehicle dynamics and road safety: Technologies, simulations, and applications," p. 3735, 2024.

[11] X. Zhang, X. Yu, X. Ye, and S. Pirouzi, "Economic energy managementof networked flexi-renewable energy hubs according to uncertainty modeling by the unscented transformation method," *Energy*, vol. 278, p. 128054, 2023.

[12] A. Altan and R. Hacıoğlu, "Model predictive control of three-axis gimbal system mounted on uav for real-time target tracking under external disturbances," *Mechanical Systems and Signal Processing*, vol. 138, p. 106548, 2020.

[13] C. Johnson, "Accomodation of external disturbances in linear regulator and servomechanism problems," *IEEE Transactions on automatic control*, vol. 16, no. 6, pp. 635–644, 2003.

[14] J. Zhou, Y. Wang, X. Zheng, Z. Wang, and H. Shen, "Weighted h_∞ consensus design for stochastic multi-agent systems subject to external disturbances and adt switching topologies." *Nonlinear Dynamics*, vol. 96, no. 2, p. 853, 2019.

[15] C. Xia, N. Liu, Z. Zhou, Y. Yan, and T. Shi, "Steady-state performance improvement for lqr-based pmsm drives," *IEEE Transactions on Power Electronics*, vol. 33, no. 12, pp. 10 622–10 632, 2018.

[16] J. Liu, Z. Huang, X. Xu, X. Zhang, S. Sun, and D. Li, "Multi-kernel online reinforcement learning for path tracking control of intelligent vehicles," *IEEE Transactions on Systems, Man, and Cybernetics: Systems*, vol. 51, no. 11, pp. 6962–6975, 2020.

[17] L. Guan, L. Guo, X. Wu, L. Xu, and X. Wang, "Path tracking control of hierarchical and refined active disturbance rejection for intelligent vehicles under heterogeneous disturbances," *IEEE Transactions on Vehicular Technology*, 2026.

[18] Z. Zhu, Q. Yao, and Y. Shi, "Intelligent vehicle adaptive robust path tracking control strategy based on mpc and adrc," *International Journal of Automotive Technology*, vol. 27, no. 2, pp. 651–670, 2026.

[19] J. Ji, A. Khajepour, W. W. Melek, and Y. Huang, "Path planning and tracking for vehicle collision avoidance based on model predictive control with multiconstraints," *IEEE transactions on vehicular technology*, vol. 66, no. 2, pp. 952–964, 2016.

[20] C. Sun, X. Zhang, Q. Zhou, and Y. Tian, "A model predictive controller with switched tracking error for autonomous vehicle path tracking," *IEEE access*, vol. 7, pp. 53 103–53 114, 2019.

[21] T. P. Nascimento, C. E. Dórea, and L. M. G. Gonçalves, "Nonholonomic mobile robots' trajectory tracking model predictive control: a survey," *Robotica*, vol. 36, no. 5, pp. 676–696, 2018.

[22] E. Kim, J. Kim, and M. Sunwoo, "Model predictive control strategy for smooth path tracking of autonomous vehicles with steering actuator dynamics," *International Journal of Automotive Technology*, vol. 15, no. 7, pp. 1155–1164, 2014.

[23] Z. Wang, K. Sun, S. Ma, L. Sun, W. Gao, and Z. Dong, "Improved linear quadratic regulator lateral path tracking approach based on a real-time updated algorithm with fuzzy control and cosine similarity for autonomous vehicles," *Electronics*, vol. 11, no. 22, p. 3703, 2022.

[24] H.-j. Zhang, J.-w. Gong, Y. Jiang, G.-m. Xiong, and H.-y. Chen, "An iterative linear quadratic regulator based trajectory tracking controller for wheeled mobile robot," *Journal of Zhejiang University SCIENCE C*, vol. 13, no. 8, pp. 593–600, 2012.

[25] Z. Ge, Y. Wang, and M. Lv, "Three-dimensional trajectory tracking guidance law based on linear quadratic regulator," in *Journal of Physics: Conference Series*, vol. 1039, no. 1. IOP Publishing, 2018, p. 012042.

[26] B. Sun, K. Cui, P. Wang, M. Xie, S. Gao, and S. Cheng, "A lateral and longitudinal control method based on linear quadratic regulator for intelligent vehicles considering future path changes," *Measurement Science and Technology*, vol. 36, no. 1, p. 015103, 2025.

[27] Y. Jiang and T. D. Palaoag, "Trajectory tracking algorithm based on model predictive control and linear quadratic regulator," in *2024 5th International Conference on Artificial Intelligence and Electromechanical Automation (AIEA)*. IEEE, 2024, pp. 1113–1116.

[28] Y. Shtessel, C. Edwards, L. Fridman, A. Levant *et al.*, *Sliding mode control and observation*. Springer, 2014, vol. 10.

[29] H. Feng, Q. Song, S. Ma, W. Ma, C. Yin, D. Cao, and H. Yu, "A new adaptive sliding mode controller based on the rbf neural network for an electro-hydraulic servo system," *ISA Transactions*, vol. 129, pp. 472–484, 2022.

[30] D. Yao, H. Li, and Y. Shi, "Event-based average consensus of disturbed mass via fully distributed sliding mode control," *IEEE Transactions on Automatic Control*, vol. 69, no. 3, pp. 2015–2022, 2023.

[31] J. Ansari, M. Homayounzade, and A. R. Abbasi, "Load frequency control in power systems by a robust backstepping sliding mode controller design," *Energy Reports*, vol. 10, pp. 1287–1298, 2023.

[32] D. Zhang, J. Hu, J. Cheng, Z.-G. Wu, and H. Yan, "A novel disturbance observer based fixed-time sliding mode control for robotic manipulators with global fast convergence," *IEEE/CAA Journal of Automatica Sinica*, vol. 11, no. 3, pp. 661–672, 2024.

[33] J. Tan, K. Zhang, B. Li, and A.-G. Wu, "Event-triggered sliding mode control for spacecraft reorientation with multiple attitude constraints," *IEEE Transactions on Aerospace and Electronic Systems*, vol. 59, no. 5, pp. 6031–6043, 2023.
