# Supplementary material for: Composite learning based finite time nonsingular terminal sliding mode path tracking control for intelligent vehicles
Source: PLoS One. 2026 Sep 1;21(9):e0357264. doi: 10.1371/journal.pone.0357264 (PMC13533401; doi:10.1371/journal.pone.0357264)
Supplement: Supplementary file 2 [file pone.0357264.s001.docx]

This manuscript proposes a composite learning-based finite-time nonsingular terminal sliding mode control (CL-FNTSMC) strategy for intelligent vehicle path tracking under parameter uncertainties and external disturbances. The proposed approach combines composite learning, nonsingular terminal sliding mode control, and a nonlinear disturbance observer to improve tracking accuracy and robustness. Lyapunov stability analysis and simulation studies are presented to validate the proposed controller. While the manuscript is generally well organized, the contribution is largely incremental and the validation is insufficient to support the claimed advantages. Therefore, I do not recommend this manuscript for publication in its current form and comments as below,

(1) The controller is mainly an integration of existing techniques, including composite learning, finite-time sliding mode control, and disturbance observation. The manuscript does not clearly demonstrate a substantial theoretical contribution beyond this combination.

(2) The motivation for introducing the composite learning mechanism is not sufficiently convincing. The manuscript does not clearly justify why eliminating the persistent excitation (PE) condition is necessary for vehicle path tracking, nor does it explicitly relate the estimated parameters to the corresponding physical vehicle parameters.

(3) The finite-time stability proof appears inconsistent with the disturbance observer, which only guarantees bounded estimation errors. The convergence properties should be stated more rigorously.

(4) The simulation study is not sufficiently comprehensive. The proposed method is evaluated only under limited simulation scenarios, without considering representative vehicle path tracking maneuvers. In addition, the manuscript lacks comparisons with recent state-of-the-art path tracking controllers, which makes it insufficient to fully demonstrate the effectiveness and superiority of the proposed method.

(5) The proposed framework simultaneously incorporates online parameter estimation, disturbance observation, nonlinear control, and adaptive learning. However, the manuscript does not discuss computational complexity, real-time implementation, controller execution frequency, or embedded hardware feasibility. These issues are particularly important for practical autonomous driving applications.
