# Supplementary material for: Composite learning based finite time nonsingular terminal sliding mode path tracking control for intelligent vehicles
Source: PLoS One. 2026 Sep 1;21(9):e0357264. doi: 10.1371/journal.pone.0357264 (PMC13533401; doi:10.1371/journal.pone.0357264)
Supplement: Peer review history

# Peer review: Decision Letter 0

**DOI:** 10.1371/journal.pone.0357264.r001

**Authors:** Xingyu Wang

19 Jul 2026

Dear Dr. Wu,

Thank you for submitting your manuscript to PLOS ONE. After careful consideration, we feel that it has merit but does not fully meet PLOS ONE’s publication criteria as it currently stands. Therefore, we invite you to submit a revised version of the manuscript that addresses the points raised during the review process.

Please submit your revised manuscript by Sep 17 2026 11:59PM. If you will need more time than this to complete your revisions, please reply to this message or contact the journal office at plosone@plos.org. When you're ready to submit your revision, log on to https://www.editorialmanager.com/pone/ and select the 'Submissions Needing Revision' folder to locate your manuscript file.

- A letter that responds to each point raised by the academic editor and reviewer(s). You should upload this letter as a separate file labeled 'Response to Reviewers'.
- A marked-up copy of your manuscript that highlights changes made to the original version. You should upload this as a separate file labeled 'Revised Manuscript with Track Changes'.
- An unmarked version of your revised paper without tracked changes. You should upload this as a separate file labeled 'Manuscript'.

If you would like to make changes to your financial disclosure, please include your updated statement in your cover letter. Guidelines for resubmitting your figure files are available below the reviewer comments at the end of this letter.

If applicable, we recommend that you deposit your laboratory protocols in protocols.io to enhance the reproducibility of your results. Protocols.io assigns your protocol its own identifier (DOI) so that it can be cited independently in the future. For instructions see: https://journals.plos.org/plosone/s/submission-guidelines#loc-laboratory-protocols. Additionally, PLOS ONE offers an option for publishing peer-reviewed Lab Protocol articles, which describe protocols hosted on protocols.io. Read more information on sharing protocols at https://plos.org/protocols?utm_medium=editorial-email&utm_source=authorletters&utm_campaign=protocols.

As the corresponding author, your ORCID iD is verified in the submission system and will appear in the published article. PLOS supports the use of ORCID, and we encourage all coauthors to register for an ORCID iD and use it as well. Please encourage your coauthors to verify their ORCID iD within the submission system before final acceptance, as unverified ORCID iDs will not appear in the published article. *Only*  the individual author can complete the verification step; PLOS staff *cannot*  verify ORCID iDs on behalf of authors.

We look forward to receiving your revised manuscript.

Kind regards,

Xingyu Wang

Academic Editor

PLOS One

Journal Requirements:

When submitting your revision, we need you to address these additional requirements.

1. Please ensure that your manuscript meets PLOS ONE's style requirements, including those for file naming. The PLOS ONE style templates can be found at

https://journals.plos.org/plosone/s/file?id=wjVg/PLOSOne_formatting_sample_main_body.pdf and

https://journals.plos.org/plosone/s/file?id=ba62/PLOSOne_formatting_sample_title_authors_affiliations.pdf

2. Please note that PLOS One has specific guidelines on code sharing for submissions in which author-generated code underpins the findings in the manuscript. In these cases, we expect all author-generated code to be made available without restrictions upon publication of the work. Please review our guidelines at https://journals.plos.org/plosone/s/materials-and-software-sharing#loc-sharing-code and ensure that your code is shared in a way that follows best practice and facilitates reproducibility and reuse.

3. Please update your submission to use the PLOS LaTeX template. The template and more information on our requirements for LaTeX submissions can be found at http://journals.plos.org/plosone/s/latex.

4. Thank you for stating the following financial disclosure:

“This work was supported by the Jiangsu Provincial Vocational Education Teaching Reform Research Project under Grant No. ZDZC584.”

Please state what role the funders took in the study.  If the funders had no role, please state: "The funders had no role in study design, data collection and analysis, decision to publish, or preparation of the manuscript."

If this statement is not correct you must amend it as needed.

Please include this amended Role of Funder statement in your cover letter; we will change the online submission form on your behalf.

5. We note that your Data Availability Statement is currently as follows: “No data was generated by this study.”

Please confirm at this time whether or not your submission contains all raw data required to replicate the results of your study. Authors must share the “minimal data set” for their submission. PLOS defines the minimal data set to consist of the data required to replicate all study findings reported in the article, as well as related metadata and methods (https://journals.plos.org/plosone/s/data-availability#loc-minimal-data-set-definition).

For example, authors should submit the following data:

- The values behind the means, standard deviations and other measures reported;

- The values used to build graphs;

- The points extracted from images for analysis.

Authors do not need to submit their entire data set if only a portion of the data was used in the reported study.

If your submission does not contain these data, please either upload them as Supporting Information files or deposit them to a stable, public repository and provide us with the relevant URLs, DOIs, or accession numbers. For a list of recommended repositories, please see https://journals.plos.org/plosone/s/recommended-repositories.

If there are ethical or legal restrictions on sharing a de-identified data set, please explain them in detail (e.g., data contain potentially sensitive information, data are owned by a third-party organization, etc.) and who has imposed them (e.g., an ethics committee). Please also provide contact information for a data access committee, ethics committee, or other institutional body to which data requests may be sent. If data are owned by a third party, please indicate how others may request data access.

6. If the reviewer comments include a recommendation to cite specific previously published works, please review and evaluate these publications to determine whether they are relevant and should be cited. There is no requirement to cite these works unless the editor has indicated otherwise.

Additional Editor Comments:

This manuscript proposes a finite-time non-singular terminal sliding mode control strategy (CL-FNTSMC) that incorporates a composite learning mechanism to address the path-tracking control problem for intelligent vehicles in the presence of parameter uncertainties, unmodeled dynamics, and external disturbances. Based on a linearized bicycle model, this manuscript establishes the dynamics of the path-tracking error and designs a piecewise non-singular terminal sliding surface to ensure finite-time convergence and avoid singularity. A series-parallel estimation model is introduced to generate a prediction error, which, combined with the tracking error, jointly drives the adaptive parameter update, thereby relaxing the continuous excitation condition required by traditional adaptive control. Additionally, a nonlinear disturbance observer is employed to estimate and compensate for lumped disturbances in real time. The manuscript demonstrates the finite-time stability of the closed-loop system using Lyapunov theory and, based on MATLAB simulations comparing PID and traditional NTSMC methods, verifies the improvements of the proposed scheme in terms of tracking accuracy, convergence speed, and disturbance rejection performance. Three reviewers provided valuable comments. Taking their feedback into account, my comments are as follows:

(1) The manuscript emphasizes the finite-time stability of the closed-loop system; however, based on the derivation of Theorem 1, it only proves that the sliding mode variable s can converge to a neighborhood of the origin within a finite time, while the tracking error x can only be guaranteed to be uniformly ultimately bounded (UUB), not strictly converging to the origin within a finite time. The manuscript equates the finite-time arrival at the sliding mode surface with the finite-time stability of the system, thereby confusing the convergence properties of the sliding mode arrival phase with those of the sliding mode motion phase; the corresponding concepts need to be clarified.

(2) The ability to achieve parameter convergence without the need for continuous excitation (PE) is the core value of composite learning; however, the manuscript merely mentions this claim in the introduction and conclusion without conducting any theoretical analysis: it neither specifies the excitation conditions required for parameter convergence under the proposed scheme nor proves the convergence of the parameter estimation error or its convergence rate.

(3) The manuscript directly combines the Lyapunov functions of the sliding-mode system, parameter adaptation, and disturbance observer and draws conclusions without rigorously analyzing the coupling effects among the dynamics of these three components: Disturbance observation errors affect the accuracy of parameter estimation, and parameter estimation errors, in turn, affect the sliding-mode control. The dynamic coupling effects among these three elements were ignored in the stability proof; the boundedness conclusion was derived solely through inequality scaling, resulting in insufficient rigor in the argumentation.

(4) Parameter uncertainty considers only minor deviations in tire yaw stiffness (–10% and –8%), without addressing perturbations in key parameters such as vehicle mass and moment of inertia, nor does it account for time-varying parameter scenarios. External disturbances are modeled solely as simple sinusoidal superimposed step functions, which differ significantly from the complex disturbances encountered during actual driving, such as random wind disturbances, variations in road surface coefficient of friction, and road gradients. These overly idealized validation conditions fail to adequately demonstrate the method’s robustness.

[Note: HTML markup is below. Please do not edit.]

Reviewers' comments:

Reviewer's Responses to Questions

**Comments to the Author**

1. Is the manuscript technically sound, and do the data support the conclusions?

Reviewer #1: Partly

Reviewer #2: Yes

Reviewer #3: Yes

**********

2. Has the statistical analysis been performed appropriately and rigorously?

Reviewer #1: N/A

Reviewer #2: Yes

Reviewer #3: Yes

**********

3. Have the authors made all data underlying the findings in their manuscript fully available?

The PLOS Data policy

Reviewer #1: Yes

Reviewer #2: Yes

Reviewer #3: Yes

**********

4. Is the manuscript presented in an intelligible fashion and written in standard English?

Reviewer #1: Yes

Reviewer #2: Yes

Reviewer #3: Yes

**********

Reviewer #1: This manuscript proposes a composite learning-based finite-time nonsingular terminal sliding mode control (CL-FNTSMC) strategy for intelligent vehicle path tracking under parameter uncertainties and external disturbances. The proposed approach combines composite learning, nonsingular terminal sliding mode control, and a nonlinear disturbance observer to improve tracking accuracy and robustness. Lyapunov stability analysis and simulation studies are presented to validate the proposed controller. While the manuscript is generally well organized, the contribution is largely incremental and the validation is insufficient to support the claimed advantages. Therefore, I do not recommend this manuscript for publication in its current form and comments as below,

(1) The controller is mainly an integration of existing techniques, including composite learning, finite-time sliding mode control, and disturbance observation. The manuscript does not clearly demonstrate a substantial theoretical contribution beyond this combination.

(2) The motivation for introducing the composite learning mechanism is not sufficiently convincing. The manuscript does not clearly justify why eliminating the persistent excitation (PE) condition is necessary for vehicle path tracking, nor does it explicitly relate the estimated parameters to the corresponding physical vehicle parameters.

(3) The finite-time stability proof appears inconsistent with the disturbance observer, which only guarantees bounded estimation errors. The convergence properties should be stated more rigorously.

(4) The simulation study is not sufficiently comprehensive. The proposed method is evaluated only under limited simulation scenarios, without considering representative vehicle path tracking maneuvers. In addition, the manuscript lacks comparisons with recent state-of-the-art path tracking controllers, which makes it insufficient to fully demonstrate the effectiveness and superiority of the proposed method.

(5) The proposed framework simultaneously incorporates online parameter estimation, disturbance observation, nonlinear control, and adaptive learning. However, the manuscript does not discuss computational complexity, real-time implementation, controller execution frequency, or embedded hardware feasibility. These issues are particularly important for practical autonomous driving applications.

Reviewer #2: This paper proposes a composite learning finite-time nonsingular terminal sliding mode control for intelligent vehicle path tracking, completes vehicle modeling and Lyapunov stability proof, and verifies performance via simulation, yet lacks sufficient practical vehicle validation:

1. Only MATLAB simulations are adopted for verification without real vehicle experiments, which cannot fully prove the practicability of the proposed control method on physical vehicles.

2.The composite learning mechanism is only verified by simulation, without comparisons with existing composite adaptive sliding mode methods to highlight its novelty.

3.Steering actuator amplitude and rate constraints are ignored in controller design, lacking constraint modules inconsistent with real steering hardware characteristics.

4.Only sinusoidal and step disturbances are applied, without real driving disturbances such as side wind and abrupt road friction, leading to insufficient test scenarios.

5.Few recent review papers on vehicle sliding mode tracking are cited, failing to clearly reflect the research’s position and innovation in the relevant field.

Reviewer #3: Dear Researchers,

Thanks for the valuable manuscript. There are some points that need to be addressed to improve the quality. Please find my comments as follows:

The theoretical contributions should be stressed in detail in the Introduction.

Please check all notations and equations carefully. Moreover, the use of English should be improved.

The advantages of the proposed algorithm over well-known algorithms should be stressed. I suggest mentioning/comparing the simulations with the results of the recent valid references.

In the introduction section, it is not enough to state the current work. It should be expanded and reconstructed. Including the motivation, the main difficulties, the main work, and the improvements compared with previous related works should be emphasised in this section.

The importance of the problem considered in this paper should be further addressed.

The types of software employed for solving the problem and for simulation experiments should be stated clearly.

The directions to further and improve the work should be expanded by adding a more detailed future recommendation section after the conclusions section.

**********

what does this mean?). If published, this will include your full peer review and any attached files.

If you choose “no”, your identity will remain anonymous but your review may still be made public.

**Do you want your identity to be public for this peer review?**  For information about this choice, including consent withdrawal, please see our Privacy Policy

Reviewer #1: No

Reviewer #2: No

Reviewer #3: No

**********

[NOTE: If reviewer comments were submitted as an attachment file, they will be attached to this email and accessible via the submission site. Please log into your account, locate the manuscript record, and check for the action link "View Attachments". If this link does not appear, there are no attachment files.]

To ensure your figures meet our technical requirements, please review our figure guidelines: https://journals.plos.org/plosone/s/figures

You may also use PLOS’s free figure tool, NAAS, to help you prepare publication quality figures: https://journals.plos.org/plosone/s/figures#loc-tools-for-figure-preparation.

NAAS will assess whether your figures meet our technical requirements by comparing each figure against our figure specifications.

Attachment Submitted filename: Comments.docx

# Author comment: Author response to Decision Letter 1

**DOI:** 10.1371/journal.pone.0357264.r002

28 Jul 2026

Thank you very much for your busy schedule to review our manuscript. See the uploaded pdf file for peer-to-peer reply.

Attachment Submitted filename: Response-scis.pdf

# Peer review: Decision Letter 1

**DOI:** 10.1371/journal.pone.0357264.r003

**Authors:** Xingyu Wang

16 Aug 2026

Composite Learning Based Finite Time Nonsingular Terminal Sliding Mode Path Tracking Control for Intelligent Vehicles

PONE-D-26-32626R1

Dear Dr. Wu,

We’re pleased to inform you that your manuscript has been judged scientifically suitable for publication and will be formally accepted for publication once it meets all outstanding technical requirements.

Within one week, you’ll receive an e-mail detailing the required amendments. When these have been addressed, you’ll receive a formal acceptance letter and your manuscript will be scheduled for publication.

An invoice will be generated when your article is formally accepted. Please note, if your institution has a publishing partnership with PLOS and your article meets the relevant criteria, all or part of your publication costs will be covered. Please make sure your user information is up-to-date by logging into Editorial Manager at Editorial Manager® and clicking the ‘Update My Information' link at the top of the page. For questions related to billing, please contact billing support.

If your institution or institutions have a press office, please notify them about your upcoming paper to help maximize its impact. If they’ll be preparing press materials, please inform our press team as soon as possible -- no later than 48 hours after receiving the formal acceptance. Your manuscript will remain under strict press embargo until 2 pm Eastern Time on the date of publication. For more information, please contact onepress@plos.org.

Kind regards,

Xingyu Wang

Academic Editor

PLOS One

Additional Editor Comments (optional):

Based on the reviewers' comments, it is recommended that the manuscript be accepted.

Reviewers' comments:

Reviewer's Responses to Questions

**Comments to the Author**

Reviewer #1: All comments have been addressed

Reviewer #2: All comments have been addressed

**********

2. Is the manuscript technically sound, and do the data support the conclusions?

Reviewer #1: Yes

Reviewer #2: Yes

**********

3. Has the statistical analysis been performed appropriately and rigorously?

Reviewer #1: Yes

Reviewer #2: Yes

**********

4. Have the authors made all data underlying the findings in their manuscript fully available?

The PLOS Data policy

Reviewer #1: Yes

Reviewer #2: Yes

**********

5. Is the manuscript presented in an intelligible fashion and written in standard English?

Reviewer #1: Yes

Reviewer #2: Yes

**********

Reviewer #1: The author has fully addressed all issues raised during the previous round of peer review. The newly added experiments and analyses further strengthen the research findings, and the quality of the revised manuscript has improved significantly. I have no further comments and recommend accepting the manuscript as submitted.

Reviewer #2: (No Response)

**********

what does this mean?). If published, this will include your full peer review and any attached files.

If you choose “no”, your identity will remain anonymous but your review may still be made public.

**Do you want your identity to be public for this peer review?**  For information about this choice, including consent withdrawal, please see our Privacy Policy

Reviewer #1: No

Reviewer #2: No

**********

# Editor report: Acceptance letter

**DOI:** 10.1371/journal.pone.0357264.r004

**Authors:** Xingyu Wang

PONE-D-26-32626R1

PLOS One

Dear Dr. Wu,

I'm pleased to inform you that your manuscript has been deemed suitable for publication in PLOS One. Congratulations! Your manuscript is now being handed over to our production team.

At this stage, our production department will prepare your paper for publication. This includes ensuring the following:

* All references, tables, and figures are properly cited

* All relevant supporting information is included in the manuscript submission,

* There are no issues that prevent the paper from being properly typeset

You will receive further instructions from the production team, including instructions on how to review your proof when it is ready. Please keep in mind that we are working through a large volume of accepted articles, so please give us a few days to review your paper and let you know the next and final steps.

Lastly, if your institution or institutions have a press office, please let them know about your upcoming paper now to help maximize its impact. If they'll be preparing press materials, please inform our press team within the next 48 hours. Your manuscript will remain under strict press embargo until 2 pm Eastern Time on the date of publication. For more information, please contact onepress@plos.org.

You will receive an invoice from PLOS for your publication fee after your manuscript has reached the completed accept phase. If you receive an email requesting payment before acceptance or for any other service, this may be a phishing scheme. Learn how to identify phishing emails and protect your accounts at https://explore.plos.org/phishing.

If we can help with anything else, please email us at customercare@plos.org.

Thank you for submitting your work to PLOS One and supporting open access.

Kind regards,

PLOS ONE Editorial Office Staff

on behalf of

Dr. Xingyu Wang

Academic Editor

PLOS One
